# Genome-wide cline analysis identifies new locus contributing to a barrier to gene flow across an *Antirrhinum* hybrid zone

**DOI:** 10.1371/journal.pgen.1012173

**Published:** 2026-07-13

**Authors:** David L. Field, Sean Stankowski, Taylor Reiter, Jitka Polechova, Desmond Bradley, Daniel M. Richardson, Annabel Whibley, Arka Pal, Daria Shipilina, Louis Boell, Melinda Pickup, Yongbiao Xue, Enrico Coen, Nicholas Barton

**Affiliations:** 1 Applied BioSciences, Macquarie University, Sydney, New South Wales, Australia; 2 School of Science, Edith Cowan University, Joondalup, Western Australia, Australia; 3 Department of Genetics, Evolution and Environment, University College London, London, United Kingdom; 4 Institute of Science and Technology Austria, Klosterneuburg, Austria; 5 University of Vienna, Department of Mathematics, Vienna, Austria; 6 John Innes Centre, Norwich Bioscience Institutes, Norwich, United Kingdom; 7 Division of Systematic and Evolutionary Botany, Department of Botany and Biodiversity Research, University of Vienna, Vienna, Austria; 8 Institute of Genetics and Developmental Biology; Chinese Academy of Sciences, Beijing, China; CNRS UMR5554, FRANCE

## Abstract

Identification of the genomic regions that contribute to reproductive isolation and how they interact is a major goal of evolutionary genetics. Much effort has focused on locating candidate genes and potential barrier loci by scanning genomes for regions of excess differentiation (*F*_*ST*_). An alternative, and perhaps more robust approach, is to scan for genomic regions exhibiting steep clines in allele frequency across a hybrid zone. We develop a computationally efficient method for approximating cline parameters for large number of loci, and apply it to genomic data from across a hybrid zone between flower colour varieties of *Antirrhinum majus* (*A. m. m* var. *pseudomajus* and *A. m. m* var. *striatum*). Most steep clines are clustered in seven genomic regions, only four of which were present from *F*_*ST*_ scans between all pair-wise comparisons. Six of these regions carry previously identified loci that influence flower colour in the hybrid zone. The seventh region harbours a novel locus*, RUBIA*, modifying magenta intensity. Clines at *RUBIA* approached fixation on the magenta side of the hybrid zone, whilst remaining polymorphic on the yellow side. This polymorphism on the yellow side may reflect a smaller phenotypic effect of *RUBIA* in yellow compared to magenta genetic backgrounds. Our findings illustrate how whole-genome cline scans in hybrid zones can robustly detect genomic regions contributing to phenotypic differences and highlight how different reproductive barrier loci interact across the genome.

## Author summary

Understanding how new species form requires identifying the genetic differences that limit gene flow between populations. A common approach is to search genomes for regions showing strong genetic differentiation between pairs discrete samples, but this can miss important loci because speciation occurs in continuous space. Here we analyse how allele frequencies change across a natural hybrid zone between two flower colour forms of the snapdragon *Antirrhinum majus*. We developed a computational method that efficiently estimates spatial allele frequency gradients (i.e., ‘clines’) for large genomic datasets. Most loci with steep clines, a hallmark of loci under natural selection, cluster in seven genomic regions. Six of these contain genes already known to influence flower colour differences between the two forms. The seventh region contains a previously undescribed locus, *RUBIA*, which modifies the intensity of magenta pigmentation. Interestingly, this locus shows different genetic patterns on each side of the hybrid zone, suggesting that its effects depend on genetic background. These results show how genome-wide cline analyses can identify genetic regions contributing to phenotypic differences and reproductive isolation in the presence of gene flow.

## Introduction

A fundamental question in evolutionary biology is how divergent populations arise and are maintained in the face of gene flow [1,2]. The separation of species ultimately requires the evolution of barriers to gene exchange that are strong enough to balance the homogenizing effects of gene flow. Much effort has focused on identifying the genetic barriers to gene flow and the traits underlying reproductive isolation [1]. The heterogenous landscape of phylogenetic relationships [e.g., 3] and genome-wide scans of population differentiation and divergence (*F*_*ST*_ and *D*_xy_) have been widely used strategies for locating barrier loci along genomes [2]. The *F*_*ST*_ scan approach assumes that regions of excess divergence (genomic islands) coincide with barrier loci whereas regions of low divergence reflect homogenization through recurrent gene flow [4,5]. However, patterns of differentiation are often complex and genomic islands may be produced by a number of processes unrelated to differential gene flow [2,6–8]. Although useful insights have been developed to account for confounding effects of background selection and recombination [e.g., 3, 9], these signals still often tell us little about the ongoing balance between gene flow and selection required to demonstrate local barriers along the genome.

Genome-wide scans for regions exhibiting geographic clines in allele frequency across hybrid zones provide a promising way to find genomic regions containing barriers to gene flow. In hybrid zones, genomes continually mix to produce new gene combinations which selection acts upon in nature. If these are less fit, either inherently or because they are in an unfavorable environment, then a stable equilibrium may be reached between gene flow and selection [10,11]. This leaves a signature of geographic clines in allele frequency, which can be quantified for each locus along the genome. Theory predicts that divergently selected alleles are expected to resist introgression and maintain steep clines, whereas neutral or advantageous alleles will exchange freely across the hybrid zone and eventually flatten out. A key strength of geographic cline analysis is that local equilibrium is reached quickly for selected (*t* = 1/*s*) and neutral loci (*t* = x^2^/$\sigma^{\text{2}}$for distance x) [11,12]. Therefore, given enough generations since secondary contact, loci under divergent selection can be located along the genome because they display steeper spatial gradients in allele frequency compared to neutral loci.

There is a long history of fitting geographic clines to small numbers of loci to estimate selection in hybrid zones [10,13], with increasing numbers of loci available as sequencing technologies improved [e.g., 14–16]. Although a few studies have begun scaling up to many thousands of loci, most still use specific loci of interest or sparse reduced representation genomic data [16,17]. A major obstacle to true genome-scale studies is that cline fitting procedures are based on Maximum Likelihood Estimation (MLE) [e.g., 18, 19]. For large numbers of loci MLE analysis represents a considerable computational burden, and there is scope to develop a faster method that is complementary to MLE. Such a method would allow for rapid scanning of genome wide cline parameters, which would be valuable for identifying barrier loci and genes responsible for divergent phenotypic traits.

Genome-wide cline analysis also raises new statistical challenges. Once cline properties are estimated genome wide, it is unclear how they will be distributed across genomes in relation to known positions of selected loci. Furthermore, it remains unclear whether selected loci can be reliably picked out against the stochasticity that we expect across large numbers of loci. Recent attempts to simulate genome wide geographic clines have been based on simulating selected and neutral loci to provide expectations for observed cline properties [e.g., 17]. However, the distribution of genome wide cline properties will depend on several parameters that may be difficult to measure in nature, including: the time since secondary contact, neighbourhood size, genome wide barriers, epistasis and local recombination rate. Thus, interpreting patterns of genome wide clines is still challenging, knowing at least some true positives (i.e., genes with known phenotypic effects under divergent selection) allows inferences to be validated and provides an important reference to compare against the genomic background.

**Fig 1:**
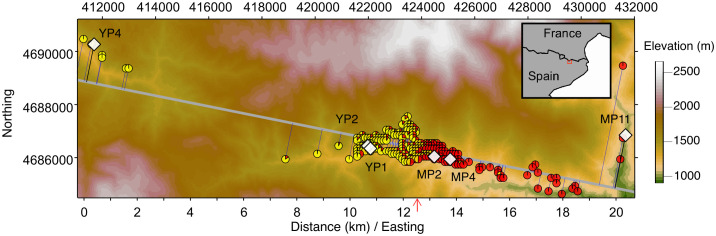
Sampling locations and allele frequencies for *Antirrhinum majus majus* hybrid zone that occurs from varieties *A. m. m.* var. *striatum* (yellow) and *A. m. m.* var. *pseudomajus* (magenta) in the Spanish Pyrenees. Sampling locations of six poolSeq whole genomes (white diamonds) and allele frequencies at *ROS* from denser sampling of individuals where each pie diagram shows the proportion of the *ROS*^*ps*^ allele from *pseudomajus* (red) compared to the *ros*^*st*^ allele from *striatum* (yellow) alleles at the *ROS* locus within a 200 metre diameter (see materials and methods). Red arrow indicates the approximate centre of the phenotype cline. A break of ~4 kilometers in the distribution of *Antirrhinum* plants coincides with a mountain pass (~3–7km along transect).

In this study, we introduce *FastClines*— a new method for approximating geographic cline parameters without numerical optimization. We use *FastClines* to locate a novel barrier locus in a hybrid zone between varieties of snapdragon *Antirrhinum majus* subspecies *majus*. This subspecies has been a model plant since the dawn of genetics [20]. Here, we focus on a hybrid zone between two varieties of *A. majus* subspecies *majus*. *Antirrhinum majus majus* var. *pseudomajus* is predominately magenta, in contrast with *A. m. m.* var. *striatum,* which is predominantly yellow (Fig 1). These phenotypes are determined by the interaction of a few large effect loci, that regulate different components of the flavanol biosynthetic pathway and influence the pattern of pigmentation of anthocyanin (magenta) and aurone (yellow). The magenta colour is primarily controlled by two tightly linked MYB-like transcription factors on chromosome 6, *ROSEA* (*ROS*) [21,22] and *ELUTA* (*EL*) [23]. Yellow colour is controlled by *SULFUREA* (*SULF*) on chromosome 4 [24], *FLAVIA* (*FLA*) and *AURINA* (*AUN*) on chromosome 2 [25], and *CREMOSA* (*CRE*) on chromosome 1 [26]. This set of flower colour genes, all of which have been shown to likely act as barriers to gene flow, makes snapdragons a useful yardstick to compare clines at barrier loci against the rest of the genome.

The two colour varieties of *Antirrhinum majus* subspecies *majus* likely diverged over many millennia, through drift and selection and came into contact recently following glacial retreat in northern Spain. Previous studies found no evidence for post-zygotic barriers [27], yet steep clines in colour phenotype and allele frequencies at *ROS* [22], *EL* [23] and *SULF* [24], are consistent with selection acting on genes responsible for magenta and yellow pigmentation. In some cases, these regions are punctuated by narrow genomic islands of differentiation [23]. Whether steep clines are restricted only to regions with known genes that influence flower colour, cluster near genomic islands, or are dispersed throughout the genome remains unclear. Knowing how clines are distributed and their properties in relation to these features, would be useful for understanding how well different approaches identify barriers and additional genes responsible for colour variation.

Using six whole genome pools spanning the hybrid zone, we ask whether genome-wide geographic clines are steeper at loci known to influence flower colour in the hybrid zone. We also ask whether these clines are clustered together along the genome, associated with genomic islands of differentiation or with known colour loci. With whole genome pools at different distances across a transect through the hybrid zone, we expect differences between the most distant pools to reflect ancient divergence. In contrast, sharp divergence between adjacent samples may reflect selection and barriers to gene flow, maintaining divergence and steep clines at particular positions along the genome. Using a novel method for cline parameter estimation, we show that most genomic regions with steep clines are tightly clustered in genomic regions containing previously identified flower colour loci. Clines are also tightly clustered in only a few regions of the genome. By interrogating a new region enriched for clinal SNPs, we use genotypes and colour phenotype associations together with RNAseq, to identify a previously undescribed colour locus. This new gene, *RUBIA* (*RUB*) modifies the intensity of magenta colouration of the flower. Clines at this locus are asymmetric, with the cline going close to fixation on the magenta side of the hybrid zone yet polymorphic on the yellow side. This asymmetry is consistent with *RUB* having a lesser phenotypic effect in a predominantly yellow background and thus is likely under weaker selection on the yellow side of the hybrid zone. By examining genome wide properties of geographic clines, our study provides a novel contribution to locating barrier loci in relation to genomic divergence and how multiple genes interact to generate phenotypic variation under selection in nature.

## Results

### Genome wide pools along the hybrid zone highlight few fixed differences

To investigate genome-wide geographic cline parameters and measures of nucleotide diversity (π), differentiation (*F*_*ST*_) and divergence (*D*_xy_), we used poolSeq to obtain whole-genome allele-frequency data from six sample locations spanning the transition in flower colour across the hybrid zone. The six sampling locations—YP4 (most western), YP2, YP1, MP2, MP4, MP11 (most eastern)—were arrayed roughly along a 1-D transect spanning 24 km of the valley floor where snapdragons are present [see 23], with tighter spacing of locations near the centre of the phenotypic cline to capture the allele frequency change either side of the transition (Fig 1; locations see S1 Table). YP4, YP2 and YP1 are located on the yellow side, while MP2, MP4, MP11 are located on the magenta side. One of the sample locations, YP4, is separated from the rest of the main transect by a mountain range that extends above the altitudinal range typically inhabited by snapdragons. The area of unsuitable habitat is around 4km wide, and likely represents a substantial barrier to dispersal for the species (Fig 1).

The sequence data covered 476.63 Mbp (93.6%) of the ~ 509 Mbp *Antirrhinum majus* reference genome v3.5 [28,29]. A sliding window approach (10kbp windows) provided a suitable span to identifying differences in diversity and divergence (see Tavares et al., 2018). After filtering (low sequencing depth, singletons and non-biallelic SNPs), a median of 79.7% sites remained within the 10,000 bp sliding windows. This produced 46,100 windows (93.7% of genome), with a minimum of 10% window coverage. Average depth within windows ranged from 22.5 for pool MP2 to 53.3 for pool YP2. Along the genome, we identified 21 million variant sites across the hybrid zone.

Allele frequencies were generally similar between the two variants of *A. m. majus*, with only 3,847 (0.018% of variant sites) exhibiting allele frequency differences (Δp) greater than 0.9 between the most geographically distant pools (YP4 and MP11) and 2,319 sites (0.011% of variant sites) exhibiting fixed differences (Δp = 1). These fixed differences were found across the genome, although chromosome 2 contained the majority with 1,995 (86%), followed by chromosome 6 with 188 (5.1%). The remaining chromosomes exhibited much lower numbers of fixed differences (between 2 on Chr 7 and 73 on Chr 4).

### Approximation of cline parameters with *FastClines*

**Fig 2:**
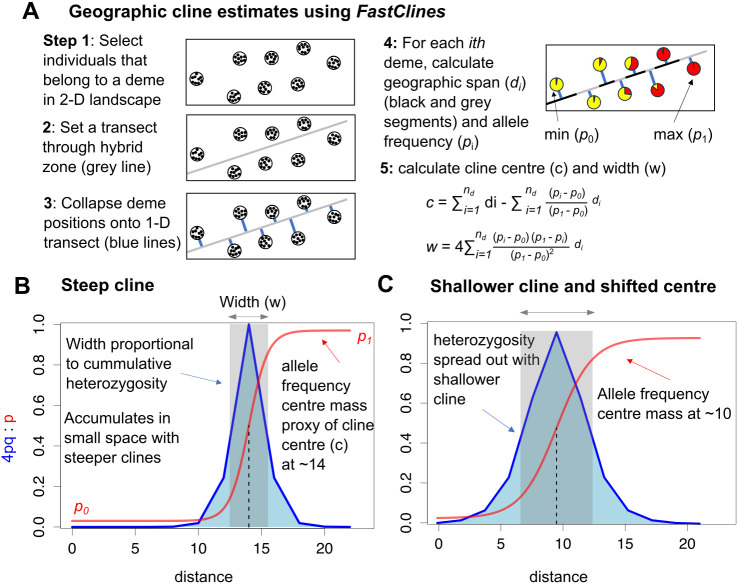
Summary of geographic clines estimation using *FastClines.* **(A)** Steps involved in estimating cline properties (centre and width) using *FastClines* with genotype data from natural populations across a hybrid zone. **(B)** cumulative heterozygosity (*4pq*) and allele frequencies (*p*) for a theoretical steep cline (width = 3, centre = 14) and the effects on the total heterozygosity across the transect (blue shaded area), **(C)** a theoretical shallower cline with a shifted centre (width = 6, centre = 10) displaying a broader spread of heterozygosity.

**Fig 3:**
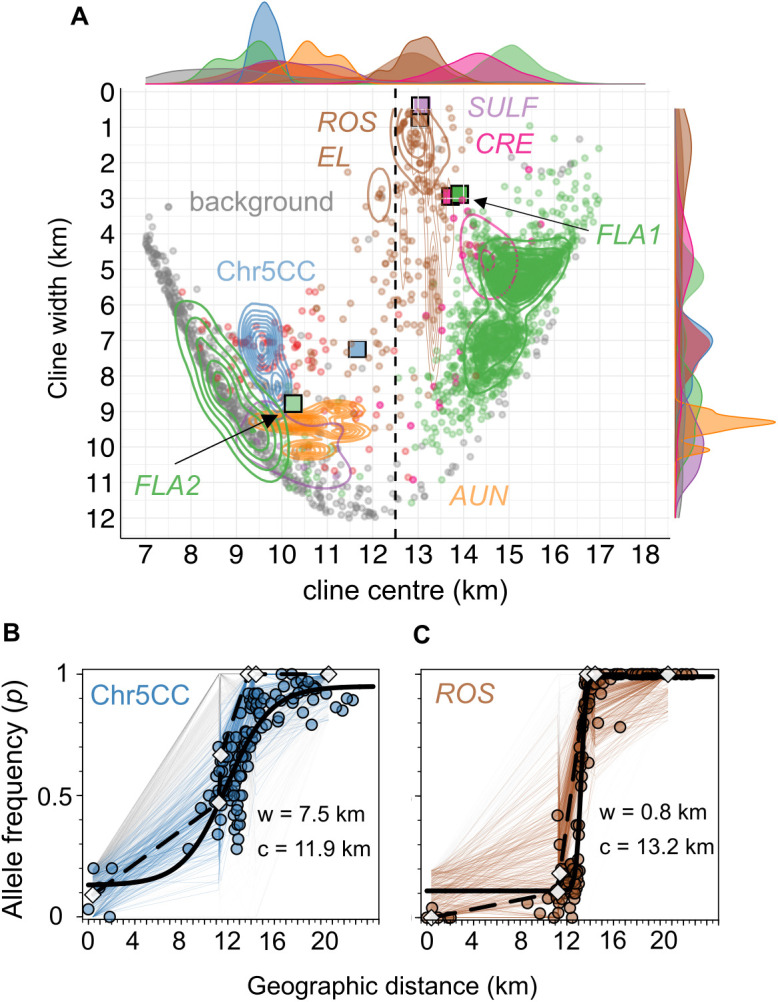
Geographic cline properties at the *Antirrhinum majus majus* hybrid zone with *FastClines* and sparse poolSeq locales compared with Maximum Likelihood Estimates (MLE) estimates and denser locale sampling SNP genotyping. **(A)** *FastClines* estimates of cline width and centre using six poolSeq locales poolSeq (circles) and Maximum Likelihood Estimates (MLE) using SNP genotyping (squares). For poolSeq data using six sampling locations, loci closely linked (<100kb) to colour genes (*ROS & EL* = brown, *CRE* = pink, Chr5CC/RUB = blue, *FLA1* = darker green, *FLA2* = light green, *SULF* = purple, *AUN* = orange) and background loci not closely linked to colour loci (grey circles). FLA1 refers to the cluster of loci on one side of the gene having centre positions > 13km, and FLA2 refers to the cluster of loci on the other side of the gene with centre positions < 13km. Contours indicate density of cline property estimates. The MLE cline parameter estimates at the four representative loci from the same regions using dense geographic sampling (>100 sampling locations). Marginal curves indicate density of cline properties for *FastClines* (the same colours as described above). **(B)** Chromosome 5 allele frequencies from poolSeq at a locus within *RUB* (white diamond and dashed black line) for loci closely linked to Chr5CC/*RUB* (blue lines, < 100kb), background loci (grey lines, > 100kb), and SNP genotyping for same locus within Chr5CC*/RUB* (blue circles) with MLE cline fit (solid black curve). **(C)** Chromosome 6 allele frequencies from poolSeq at a locus within *ROS* (white diamond and dashed black line) for loci closely linked to *ROS1* (brown lines, < 100kb), background loci (grey lines, > 100kb), and SNP genotyping for same locus within Chr5CC*/RUB* (brown circles) with MLE cline fit (solid black curve).

To facilitate the estimation of geographic cline parameters for whole-genome data, we developed a new method for the rapid approximation of cline parameters. The challenge with the most commonly used MLE method is that it is computationally demanding for whole genome data. A simple surrogate is to estimate cline width via total heterozygosity and cline centre via the median mass of allele frequencies along a 1-dimensional transect [30]. We extend this approach in a method we call *FastClines* to account for non-diagnostic loci and uneven positioning of sampling locations along a cline (Fig 2, see Materials and Methods for in-depth description and benchmarking against MLE fitting). We use *FastClines* to estimate parameters to estimate cline centres and widths for 3,847 highly divergent loci (Δp ≥ 0.90 between most distant pools YP4 and MP11). Cline width estimates ranged from ~12.5km to 0.8km, narrowing as the cline centres moved either side of the hybrid zone (< 8km and >15km) (Fig 3). This narrowing of cline widths towards the edges is consistent with simulations of sub-sampling along a cline (S1 Fig). More specifically, sub-sampling of six sampling locations along a chain of demes produces this effect which diminishes with 12 or 24 locales sampled (see S1 Fig). Therefore, in the current *Antirrhinum* data set, we cannot accurately compare the steepness of clines that are shifted far to the left or right and restrict subsequent analyses to clines centred between 9km and 15km (see Fig 3). Despite this artefact, comparisons of simulations of steeper and shallower clines showed quite distinct distributions of width estimates, even with sparse local subsampling of six or eight locales (S1 Fig). Cline centers and widths are more difficult to distinguish when differences are small and when loci become less differentiated on either side of the cline (S1 and S2 Figs). Increasing variance in allele frequencies around the cline and decreasing sequencing depth also make clines more difficult to distinguish.

### Major effect flower colour loci are associated with steep clines

**Fig 4:**
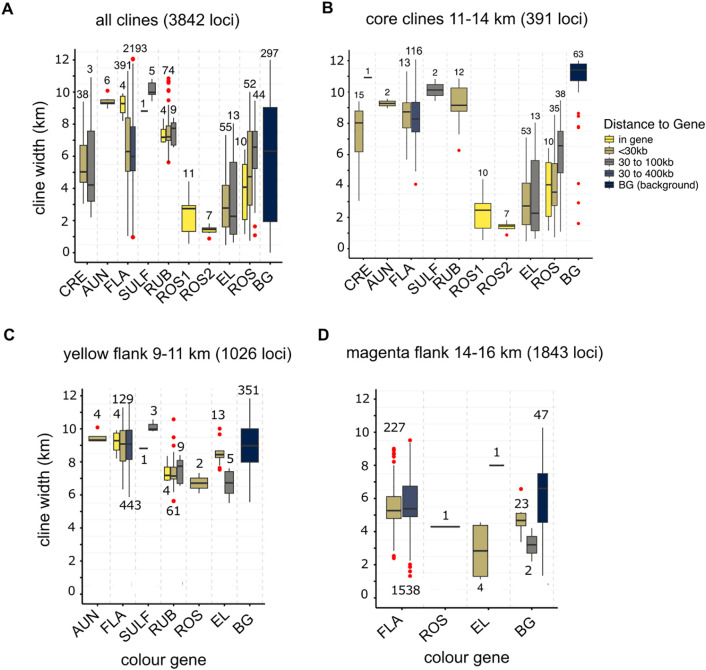
Summary of cline numbers and cline widths at different distances for colour loci for (A) all clines, (B) clines centred in the core region close to phenotypic transition, (C) clines centred towards the yellow flank of hybrid zone, (D) clines centred towards the magenta flank of the hybrid zone. Clinal loci grouped by proximity to colour loci including within the coding sequence of the gene (inGene), tightly linked <than 30kb, either 30 to 400 kb for *FLA* gene due to low recombination region, 30 to 100 kb (for all other colour loci) and background loci (>400kb near *FLA* region and >100kb elsewhere). *ROS1* and *ROS2* are loci within MYB genes, whereas *ROS* and *EL* are tightly linked (<30kb) yet outside the coding regions of MYBs. Note that boxplots are only show for loci when they had clinal loci centred within each of the given geographic ranges.

We next mapped the genomic location of all genes known to influence flower colour to compare cline properties. For 391 loci with cline centres nearest to the centre of phenotypic cline (between 11 and 14 km), the steepest clines were at loci closely linked to *ROS* and *EL*, the two major effect loci important for regulating magenta pigmentation (Fig 3). For the variant sites around and within *ROS* and *EL*, clines were steepest within coding sequences for the genes, with mean cline width of 2.2km (±1.2km SD) within ROS1, 1.3km (±0.3km 255 SD) and 3.5km (±2.3km SD) within EL (Fig 4A). In this genomic region, variant site clines tended to become wider the further they were from the coding sequences of these two genes. For example, 3.9km mean width for loci <30kb from *EL*, increased to 5.8km for loci 30–100kb away (Fig 4A).

The ability of *FastClines* to detect other major colour loci depended on their cline centre estimates, which showed a range of positions either side of the main phenotypic transition, with a bias toward the eastern (Magenta) side (n = 1834 vs n = 391 near phenotypic centre). The discordance in cline centre among loci was strongly associated with genomic location, with 1765 of the 1843 loci (95%) with cline centres between 14.5 to 15.km (~2–3 km East of phenotypic centre at 12.5km) occurring on chromosome 2 immediately downstream of the *FLA* gene (see *FLA1* for downstream sites on Fig 3A). In contrast, clinal loci within or upstream of the coding sequence of *FLA* showed cline centres < 13 km (~1.5 km West of phenotype centre at 12.5km; see *FLA2* for upstream sites on Figs 3A; see S5). The majority of ‘background’ loci which were greater than 300kb from known colour loci exhibited cline centres shifted into the yellow side (grey points; Fig 3A). These loci showed a typical pattern of high frequencies of the common allele from the magenta flanks in five of the six pools, followed by a rapid drop in allele frequency in the YP4 pool located on the other side of the pass (~4–7km on transect). This pattern of allele frequency change was evident for loci not closely linked to colour genes (>300kb *FLA* on Chr 2 see S6 Fig; > 100kb from *CRE* on Chr 1 see S7 Fig, > 100kb from *SULF* on Chr 4 see S8 Fig; > 100kb from Chr5CC see S9 Fig; > 100kb from *ROS*/*EL* on Chr 6 see S10 Fig) and for most ‘background’ loci on chromosomes which do not possess any known colour loci (Chr 3, Chr 7 and Chr 8 see S11 Fig).

Next we focus on loci with clines positioned closer to the phenotype transition between 8–16km, as these are most likely to be causally associated with variation colour. Given the tendency for width estimates to narrow as cline position shifts to the edge of the transect, together with the sharp allele frequency step for loci positioned over the mountain pass (~3–7km along transect), this ensures we are dealing with true monotonic changes in allele frequencies expected of geographic clines shaped by selection within the hybrid zone. Of the colour loci, *ROS* and *EL* showed the narrowest clines (Figs 3; 4), while *FLA* and *CRE* showed wider clines that were still distinguishable from the genomic background. The *AUN* genomic region was associated with a substantial number, although these were not particularly steep. At the *SULF* colour locus, this region was not well covered by poolSeq data due to a known deletion in the *A. m. m. striatum* haplotype, although some KASP markers have been developed flanking this deletion and show steep clines similar to *ROS* (Fig 3A; see Bradley et al., 2017).

We also found a narrow, previously uncharacterized genomic region on chromosome 5, which we refer to as Chr5CC (Chromosome 5 Cline Cluster), which showed a large cluster of clines positioned between 8–11 km. Clines within Chr5CC had an average width of 7.52km (±1.04km SD), substantially narrower than 10.24km (±0.91km SD), the average width of other clines with 8–11km centre estimates.

We next compare cline width and centre estimates at a subset of six loci from *FastClines* (six sampling locations) to Maximum Likelihood Estimates (MLE) cline fits for much denser geographic sampling (>100 sampling locations) using KASP SNP genotyping. The MLE cline fits used a polymorphic sigmoid model (see materials and methods; 5-parameters; centre, width, p0, p1, *F*_*ST*_) and cline properties (width and centre) were compared at loci linked to colour genes *ROS*, *EL*, *SULF*, and recently identified genes *FLA*, *CRE*, and Chr5CC. Cline width for MLE fits was broadly consistent with the major grouping of cline width estimates from *FastClines* at *ROS*, *EL*, *CRE*, *FLA* and Chr5CC (circle symbols *FastClines* fits vs. square symbols for MLE cline fits in Fig 3; S3 Table). The exception was the SULF gene interval on Chr 4 which contained KASP SNPs with steeper clines in comparison to the widths estimated for the majority of poolSeq sites using *FastClines*. However, this was due to the loci genotyped with KASP being excluded from the *SULF* region in the poolSeq due to low Δp and coverage. Cline centre estimates from *FastClines* had a wider range than those from MLE cline fitting. MLE cline fitting showed a much tighter grouping of cline centre estimates around the phenotypic centre (Fig 3). However, the step change in cline centre positions either side of the FLA gene were consistent between both cline fitting approaches (FLA1 locus and FLA2 locus on Fig 3).

### Geographic clines are genomically widespread but cluster around colour loci

**Fig 5:**
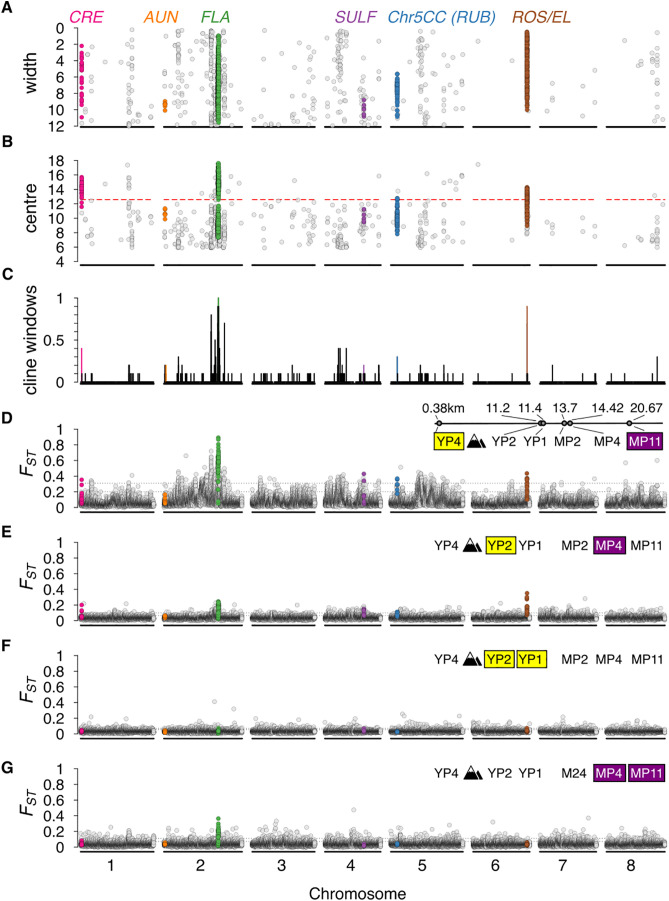
Genome scans between whole genome pools of *A. m. m.* var. *striatum* (yellow) and *A. m. m* var. *pseudomajus* (magenta). Panels show **(A)** cline width with *FastClines*, **(B)** cline centre with *FastClines* **(C)** cline frequency in windows (from *FastClines*) in 10kb windows, **(D)** *F*_*ST*_ pools YP4 x MP11 in 10kb windows (different colours and over mountain pass), **(E)** *F*_*ST*_ pools YP2 x MP4 (different colours and no mountain pass), **(F)** *F*_*ST*_ pools YP2 x YP1 (same colours and no mountain pass), **(G)** *F*_*ST*_ pools MP4 x MP11 (same colours and no mountain pass). Dashed vertical lines indicate phenotype cline centre (red), and 99^th^% and 95^th^% quantiles for *F*_*ST*_ panels (black dashed). Insert in panel (D) indicates relative positions of the six pools along the transect (in km). Window *F*_*ST*_ within 100kb of colour loci are coloured separately for CRE (pink), AUN (orange), FLA (green), SULF (purple), RUB (blue), ROS and EL (brown).

We next explored the genomic distribution of clinal loci and their cline parameters along the genome. Cline widths ranged from steep to wide within all chromosomes (Fig 5A), whereas cline centres tended to be clustered within chromosomes, with substantial variation observed among chromosomes (Fig 5B). For example, the majority of background loci across all chromosomes and some loci linked to colour loci (*AUN*, *SULF*) exhibited cline centres shifted towards the yellow flank whereas other loci (*ROS*, *CRE*) were centred towards the phenotypic transition or slightly towards the magenta flank. To understand how divergent loci with clines were distributed across the genome, we divided each chromosome into 100kb and 10 kb blocks (non-overlapping for permutations, see below) and counted the number of loci with allele frequency differences (Δp) greater than 0.9 between pools 1 (YP4) and 6 (MP11) that also exhibited clines in each block. Only 3.7% of 100 kb blocks contained at least one clinal SNP. Furthermore, considering only 100 kb blocks with at least one cline, the mean percentage of smaller 10kb windows (within each 100kb block) with at least one clinal loci was 19.3%, indicating clines are often clustered within the genome (Fig 5C). The main exceptions included one region of chromosome 2, with a ~ 500kb block of the genome with 90–100% of 10kb windows within the block containing clinal loci. The other exception was on chromosome 6, with all 10kb windows in one 100kb block containing clinal loci (Fig 5C).

Differentiated loci with cline centres between 8 and 16 km (i.e., excluding loci with step changes in allele frequency over mountain pass) were significantly more clustered than expected by chance on chromosomes 2 and 6, but not significantly clustered on the remaining chromosomes (Fig 5A; S4 Table). This indicates that some chromosomes exhibit tight concentrations of clines in localized areas of the genome, whereas others exhibit scattered clines along the entire chromosome (Fig 5). Moreover, clustering tended to occur in close proximity to the six known genes involved in regulating flower colour. The majority, n = 2918 (76.2%) of divergent loci with clines were located within 300kb of known colour genes (Fig 5). Dense clusters of clines are found in small genomic regions tagged *ROS*, *EL*, and *SULF*, and recently discovered *CREMOSA (CRE)* [26], *FLA* [25] and a gene located at Chr5CC (see below). Colour loci were located on the chromosome (Chr) block with the highest frequency of windows with clines on Chr 1 (CRE) Chr 2 [FLAVIA; 25], and Chr 6 (*ROSEA*/*ELUTA*), except for *SULF* on Chr 4 which was located in the 4^th^ ranked block (Fig 5C).

### Comparison between *F*_*ST*_ and cline scans for identifying barrier loci

**Fig 6:**
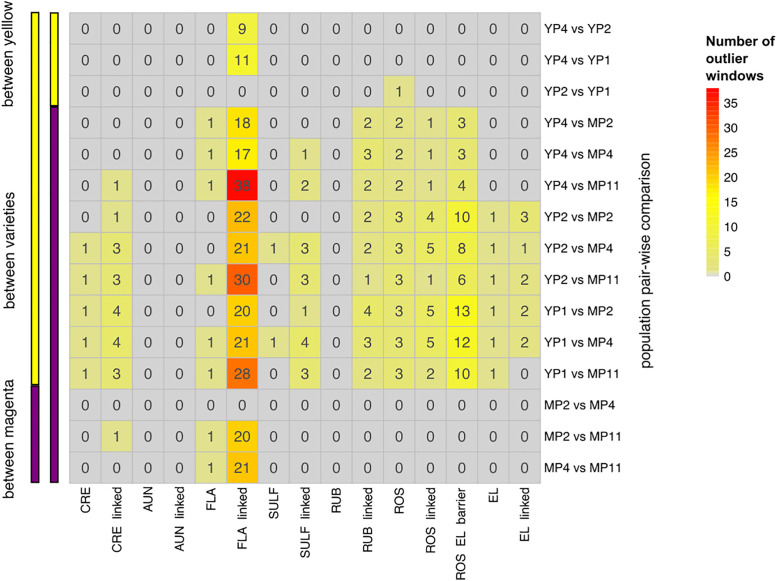
All pair-wise population comparisons of *F*_*ST*_ and the number of 10kb windows in colour genes and closely linked regions along the genome. All pair-wise population pairs shown in rows, with the main colour of the varieties in each pair indicated (left colour bars) highlighting comparisons between yellow and magenta or within variety outliers. Columns indicated the number of windows in the 99^th^ % quantile for *F*_*ST*_ at each of the colour genes and also regions closely linked (<100kb). For the *FLA* gene, closely linked regions are larger (<400kb) due to this being an area of low recombination in the genome. Numbers in each cell indicate how many 10kb windows were detected in the 99^th^ % quantile. Legend displays the colour match for number of outlier windows.

To understand how *F*_*ST*_ associates with colour loci and geographic clines, we first examined the number of 10kb *F*_*ST*_ outlier windows, defined as loci with *F*_*ST*_ values in the upper 99^th^% quantile, that intersect with colour genes. In our study, *F*_*ST*_ was calculated between each pair of pools, and estimates are expected to vary among pairs, reflecting both the arbitrary location of the 6 pools and geographic distance between them. In contrast to geographic cline analysis, which is informed by allele frequency variation over all of the pools, elevated *F*_*ST*_ was less consistent in recovering known and recently identified colour loci. The nine pair-wise Fst comparisons of yellow and magenta pools revealed striking discordance among the *F*_*ST*_ landscapes. Only the *ROS* gene intersected with *F*_*ST*_ outliers in all pair-wise comparisons for at least one 10kb window (Fig 6). The *CRE* gene and *FLA* displayed windows in *F*_*ST*_ outliers in five and six of the pair-wise comparisons, respectively. This was followed by *SULF* present in only two of nine, and both Chr5CC and *AUN* were not present in any pair-wise comparisons (Fig 6). Some windows of excess *F*_*ST*_ were identified even between pools of the same flower colour variety (e.g., *FLA* linked YP4 vs YP2 and MP2 vs. MP11; Fig 6). Relaxing the criteria for for identifying *F*_*ST*_ outliers from the upper 99^th^% quantile to the 95^th^% quantile increased the number of outlier windows in each colour gene region (S14 Fig), yet some comparisons between colour varieties still showed no *F*_*ST*_ outlier windows associated with known colour loci. Permutation tests revealed an enrichment of clinal loci within *F*_*ST*_ outlier windows for most pair-wise comparisons. However, for some pair-wise comparisons many *F*_*ST*_ outlier windows contain no cline outliers. For example, for the most distant populations, YP4 and MP11, only 69% of outlier *F*_*ST*_ windows have clines (S4 Table). This percentage of *F*_*ST*_ outlier windows with clines diminished as closer pools of yellow and magenta are compared (e.g., YP3 and MP4 and Δp>0.9, 27% of *F*_*ST*_ outlier windows has clinal loci; S4 Table). Compared to all metrics, *F*_*ST*_ was consistently elevated for windows containing clinal loci compared to windows without clinal loci (S5 Table).

### *RUBIA* locus controls magenta intensity

We next interrogated a set of steep clines from the Chr5CC genomic region which had cline centres to the left of the core geographic region (around 9–10km) (Fig 3). This region was the top ranked cluster of clines on chromosome 5, with 87 divergent loci with geographic clines within a tight genomic region spanning 70kb (Figs 5, S9). As no genes associated with colour phenotypes had been previously identified in Chr5CC, we tested for genotype-phenotype associations.

**Fig 7:**
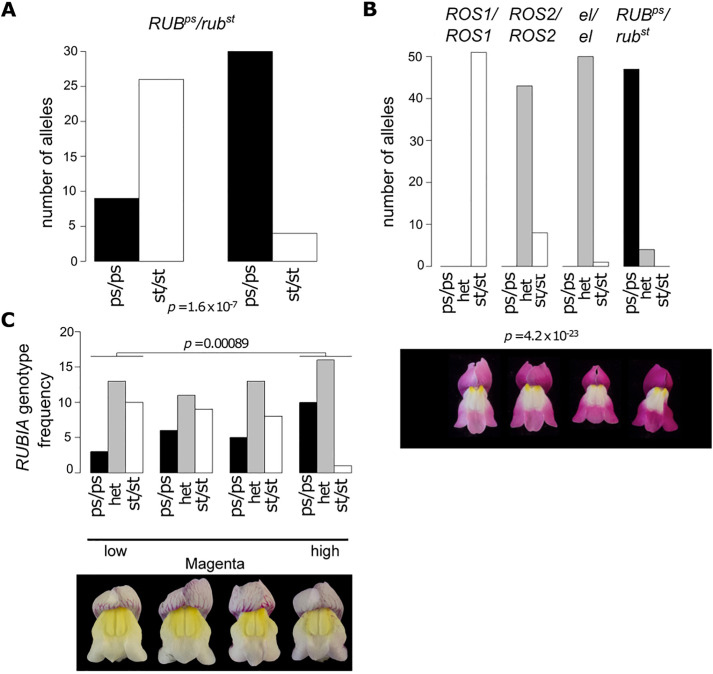
(A). Flower colour ranking results for an *F*_*2*_ hybrid population segregating for Chr5CC (subsequently named *Rubia*) and their genotypes at colour loci *ROS*, *EL*, and *SULF*, grouped by shared genetic backgrounds. Counts of the *A. m. m. pseudomajus* (*RUB*^*ps*^) and *A. m. m. striatum* (*rub*^*st*^) homozygotes, summed across all genotypic groups, are shown in black and white. **(B)** Flower colour ranking results for an *F*_*4*_ hybrid population segregating for Chr5CC. Photographs were ranked according to magenta intensity, yielding four quartiles of increasing magenta. Counts of the *A. m. m. pseudomajus* and *A. m. m. striatum* homozygotes are shown in black and white, with heterozygotes (het) shown in grey. The genetic background of the *F*_*4*_ family is shown above the figure. Representative flower photographs are shown below each quartile. **(C)** Flower colour ranking for an F4 population that was *ros*^*st*^ *El*^*st*^*/ros*^*st*^ *El*^*st*^ and segregating for *RUB* alleles. As in **(B)**, the flowers were ranked for magenta and genotypes analysed in quartiles shown with representative flowers of each quartile shown below. Significance test (p values) from chi-squared test in each panel.

To determine whether Chr5CC carried a previously unidentified locus affecting flower colour, we analysed populations derived from crosses between *A. m. m. var. striatum* and *A. m. m var. pseudomajus*. Flower photographs were grouped according to *ROS*, *EL*, and *SULF* genotypes. For each group, we carried out three independent rankings of flowers according to magenta intensity, yielding two bins (Fig 7A). Ranked flowers were also genotyped for SNPs from Chr5CC. Pooling SNP frequencies across genotypic classes showed that the frequency of *A. m. m var. pseudomajus* SNPs for Chr5CC was significantly enriched in the high magenta bin, and depleted in the low magenta bin (*χ*^*2*^ test, *p* = 1.6 x 10^-7;^ see S4 Text). These results suggest that Chr5CC harbours a magenta flower colour locus, hereafter named *RUBIA* (*RUB*).

To further test this hypothesis, we genotyped *F*_*4*_ populations that were homozygous at *ROS EL* and *SULF*, but segregating for Chr5CC SNPs, and repeated the ranking process. To infer genetic dominance relationships between *A. m. m var. pseudomajus* and *A. m. m var. striatum* SNPs, we split the rank into four quartiles. Ranking according to magenta intensity showed a depletion of *A. m. m. var. pseudomajus* Chr5CC SNPs in the low magenta quartile, and a depletion of *A. m. m. var. striatum* Chr5CC SNPs in the high magenta quartile (*χ*^*2*^ test, *p* = 4.2 x 10^-23^). This suggests that the *A. m. m var. pseudomajus RUB* allele, denoted *RUB*^*ps*^, increases magenta (Fig 7B). The two middle quartiles were populated almost exclusively by heterozygotes showing intermediate magenta intensity, suggesting that *RUB*^*ps*^ and *rub*^*st*^ are alleles are semidominant (see photos S5 Text).

To determine whether *RUB* alleles modified pigment intensity in a background carrying striatum alleles at *ROS, EL* and *SULF,* we analysed a population homozygous for *ros*^*st*^ *EL*^*st*^ and *sulf*^*st*^ segregating for *RUB* alleles. We ranked the flowers for spread of yellow or magenta and genotyped them for *RUB*. Comparison of the upper and lower quartiles revealed no association with yellow but a significant association of *RUB*^*ps*^ allele with high magenta and *rub*^*st*^ with low magenta (*p* = 0.00089) (Fig 7C). Though significant, the effect on magenta was more subtle in this background than in the *pseudomajus ROS*^*ps*^ *el*^*ps*^ *SULF*^*ps*^ background (compare Fig 7B with 7C). Although subtle to the human eye, the effect of a magenta tint in normally yellow regions of the flower may be to reduce colour contrast for pollinators.

**Fig 8:**
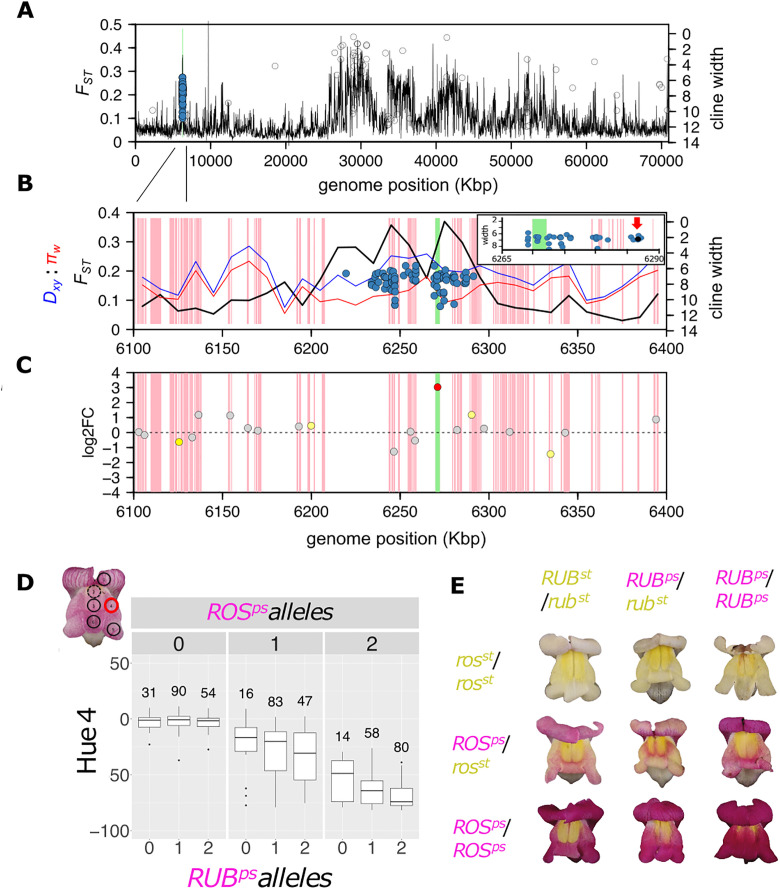
Cline cluster on chromosome 5 in relation to *RUB* locus. **(A)** Genome scan of *F*_*ST*_ (black line), *D*_*xy*_ (blue line) π_w_ (red line) along chromosome 5 for pools YP4 vs MP11, **(B)** Genome scan of *F*_*ST*_*, D*_*xy*_, π_w_ (YP4 vs MP11) and cline widths (blue circles) from poolSeq for the 300kb region on chromosome 5 (20kb within inset) with the largest cluster of clines on the chromosome (Chr5CC). *D*_*xy*_, π_w_ multiplied by 10. Annotated genes (pink regions), the location of Flavanol Synthase (*FLS*) (green region) and location of KASP SNP marker for *RUB* (red arrow in inset), **(C)** differential gene expression (DGE) from RNAseq for floral tissue of three *A. m. m* var. *pseudomajus* (magenta) vs three *A. m. m* var. *striatum* (yellow) plants indicating transcripts with no significant DGE (grey), weakly significant (yellow) and strongly significant (red). **(D)** colour phenotype scores for Hue in location 4 on flowers for the three *RUB* genotypes (0,1,2) at each of the three *ROS* genotypes (panels, 0,1,2) where numbers represent three genotypes and copy number of *A. m. m* var. *pseudomajus* alleles, **(E)** example of phenotypes with median Hue 4 values for haplotypes at *ROS* and *RUB* from hybrid zone.

This result predicts that the *RUB* genotype should correlate with magenta flower colour in plants sampled from the hybrid zone. To test this prediction, we used KASP SNP genotypes at *RUB* together with quantitative colour phenotyping for plants along the transect at the hybrid zone. To test for associations at the *RUB* locus, we used linear regressions between colour (HSV space; Hue, Saturation and Value in six floral regions) and the SNP markers closely linked to *RUB* and *ROS1.* We also included interaction effects between *RUB* and *ROS1* which improved model fits (*F* = 12.9, *p* = 0.00035). The overall model explained a significant amount of variation for Hue (Adj *R*^2^ = 0.49) with a strong effect of *ROS1* (*p* = 1.8 x 10^-09^) but not *RUB* (*p* > 0.05). However, a significant interaction effect between *RUB* and *ROS1* on Hue was detected across central regions of the flower (e.g., Hue 4, *F* = 151.5, *p* = 2.2 x 10^-16^, Fig 8D) (for all floral regions see S7 Table, for all Hue, Saturation and Value metrics see S16 Fig). These results suggest an epistatic gene interaction, whereby the *RUB*^*ps*^ allele from *A. m. m* var. *pseudomajus* enhances the intensity of magenta in these floral regions on high magenta backgrounds (*ROS*^*ps*^/*ROS*^*ps*^ and *ROS*^*ps*^/*ROS*^*st*^) but not on low magenta backgrounds (*ros*^*st*^*/ros*^*st*^) (Fig 8D and 8E). However, it is possible that an effect in the low magenta background would have been missed given the subtle signal detected in the crosses (Fig 7C). Also, we cannot rule out the possibility that a recombinant *RUB* allele that is functionally similar to that in *A. m. m.* var *striatum* but that carries *pseudomajus* markers, has introgressed into *A. m. m.* var *striatum,* raising the apparent frequency of *pseudomajus* alleles on the left flank.

At the centre of the chromosome 5 region carrying *RUBIA* was a gene encoding flavanol synthase (*FLS*) a key enzyme involved in the Anthocyanin colour pathway (Fig 8B). The SNP marker used for MLE clines and in genotype-phenotype associations was ~ 150kbp upstream of *FLS* with eight annotated genes identified within this interval (Fig 8B inset). The *FLS* region also exhibited slightly elevated *F*_*ST,*_ with some of the closely linked windows showing higher *F*_*ST*_ (depending on which yellow and magenta pool compared; Fig 6). Elevated *F*_*ST*_ *was* not strongly driven by low diversity (π) or excess divergence (*D*_*xy*_) (Fig 8B).

Next, we aimed to confirm whether any genes in this region displayed differential gene expression linked to phenotypic differences. Using RNAseq data from corolla (petal) tissues from two sets of yellow and magenta plants sampled from seed in the flanks of the hybrid zone, transcripts were compared for differential gene expression across the genome. Sorting of the transcripts by the degree of differential expression revealed that the sequences in tight linkage with the *FLS* gene displayed the highest differential gene expression levels on the entire chromosome (Fig 8C). This finding is consistent with *FLS* being responsible for tissue specific differences between the subspecies, in this case colour pigments as the only trait that clearly differs between the two groups. Here, expression of *FLS* is high in *A. m. m* var. *striatum* (yellow) and low in *A. m. m* var. *pseudomajus*.

## Discussion

### An efficient alternative for surveying geographic clines across genomes

Our new approach for estimating genome wide geographic clines has enabled a rapid genome wide perspective on cline properties. In comparison to Maximum Likelihood Estimate (MLE) cline fitting, *FastClines* has a significant advantage in that it is very fast and does not require numerical optimization using a simulated annealing procedure (e.g., [19]). Another advantage of the method is the ability to obtain reasonable estimates of cline properties with as few as six sampling localities along a transect through a hybrid zone. Although this method provides an efficient alternative to MLE cline fitting, it also has some limitations that must be kept in mind. First, for computational speed, this method defines the cline centre as the median mass of allele frequency and the cline width via the total heterozygosity. However, these estimates could be different to the steepest point and the inverse of the steepest gradient from a sigmoid geographic cline, for example, when ‘stepped’ geographic clines have asymmetric shapes [12]. Thus, MLE fitting and model selection remain the gold standard where the aim is to estimate biological parameters, such as the selection strength or barrier strength.

Although estimates of cline parameters can be obtained with a few unevenly spaced sampling locations, this will likely introduce biases in the positions of cline centres and widths. However, simulations show that this can be overcome by increasing the number of sampling locations. The accuracy of parameters inferred by *FastClines* also increases as the number of samples within each location and the depth of sequencing increases. While still challenging for many systems, high-resolution spatial population genomic datasets are becoming easier to achieve as sequencing costs continue to fall. Even where large datasets are available, comparison of *FastClines* results against MLE cline fitting at a representative set of loci is recommended for validating parameter estimates.

### Clines detect known geographic and genetic barriers

We demonstrate how a genome wide perspective on geographic clines can be a useful approach for identifying areas of the geographic and genomic landscapes that are associated with barriers to gene flow. First, our analysis revealed a concertation of clinal outliers, characterized by step-changes in allele frequency between pools YP4 and YP2. We believe that this area represents a substantial geographic barrier to the dispersal of *Antirrhinum*, as it is associated with a mountain range that rises well above the typical altitudinal limit of snapdragons. Regular surveys of this area, which is associated with heavy winter snowfall and alpine vegetation, have failed to find stable populations of *Antirrhinum*. Together with the significantly different *F*_*ST*_ landscape when comparing YP4 with all other pools in Planoles valley [23], it appears that this yellow population has experienced little genetic connectivity with populations of other side of the range. A similar pattern of genome-wide differentiation to that observed between YP4 and the other pools has also been observed at a second location, near the village of Avellanet, located 50 km to the west [31]. The distribution of snapdragons at Avellanet is much patchier than at Planoles, owing to drier, more exposed climatic conditions. Although we have never observed contact of the magenta and yellow varieties at Avellanet, where they are separated by around 1 km, hybrid individuals have been observed in the intervening area in some years suggesting past contact.

If the geographic breaks at Planoles and Avellanet represent geographic barriers to dispersal to snapdragons, then these contrasts may provide us with glimpse of what levels of genome-wide differentiation were like prior to extensive secondary gene flow in the valley at Planoles. However, there also alternative explanations. First, the two yellow populations driving the pattern (YP4 and AveS^y^) may simply be small isolates that have experienced strong drift, rather than being broadly representative of historical levels of differentiation between the colour varieties. It is also possible that these discontinuities have become trapped in areas of low population density at both locations following secondary contact, as is predicted by tension zone theory [11]. Under this scenario, pollinator-mediated selection at Planoles may have pulled the colour clines away from the multilocus barrier. Although we think that this scenario is less likely, further work is needed to understand how history, environmental variation, and pollinator mediated selection have interacted to shape variation across the species range.

Within the valley of Planoles where colour varies, we observed sharp clines at closely linked to the previously identified colour genes. These were generally narrower than the genomic background, which is consistent with the premise that these variants of *Antirrhinum majus* are primarily distinguished by small numbers of large effect genes. The majority of clines were clustered in tight physical linkage near these major effect genes controlling flower colour. The overall low frequency of fixed differences and tight clusters of clinal loci in narrow genomic regions is also consistent with ongoing gene flow and homogenization across most of the genome [23] with only a small number of barrier loci remaining differentiated in the face of gene flow. This supports theory predicting that divergently selected alleles resist introgression and maintain steep clines, whereas neutral or advantageous alleles will exchange freely across the hybrid zone and eventually flatten out. Theory also predicts that equilibrium should be reached quickly for selected loci (*t* = 1/*s*) [11,12]. Considering the age of the hybrid zone is at least 100 generations old [23], the cline widths for even the most weakly selected colour loci should have reached equilibrium.

The total number of clinal loci and how they are distributed along genomes remains a challenging property to survey even for whole genomes. For the *Antirrhinum* hybrid zone, we intentionally placed three whole genome pools to the left and three to the right of the flower colour transition. This approach likely contributed to *FastClines* estimates identifying each of the major flower colour loci and identification of a new locus. Yet we still have very different power to detect clines if they are centred close to the floral phenotype transition, compared to clines that are displaced geographically elsewhere. The second major challenge is how to interpret differences in cline density along the genome. For example, we found large differences in the number of clinal loci around the *FLA* locus compared to the next largest cluster of clines around the *ROS* locus. Given local recombination rates have been estimated as significantly lower at *FLA* compared to *ROS* [25], this suggests the local recombination landscape in which selected loci are embedded is important for interpreting cline density. For a barrier locus, lower recombination rates increase the extent of indirect selection on neutral loci, slowing the exchange of alleles through recombination between neutral and barrier loci across the genome. However, even with comparable recombination rates, the number of individual tightly linked barrier loci will influence how many clines are detected. Theory predicts the effects of indirect selection around a single site to be more localised than for a region that contains two or more tightly linked barrier loci. This is because recombination will decay admixture LD less effectively in the multilocus barrier region. Thus, indirect selection will lead to a stronger barrier in the multilocus case, affecting more of the genome. Therefore, expectations on the numbers of clines along the genome is complex and by itself not a good indication of the importance of the area as a barrier. Interpreting cline density will require consideration of initial divergence (Δp) local recombination landscape and the genetic architecture of barriers.

Differences in the geographic position of clines at independent barrier loci also provide important insight into the role of gene interactions. With both anthocyanin and aurone pigmentation interacting to constitute the parental phenotypes (magenta vs yellow), theory predicts these loci will be synergistically coupled (concordant cline centres) upon secondary contact due to the influx of parental gene combinations and linkage disequilibrium [11]. Although there is some evidence of different cline centres, the range of cline centres was likely overestimated with *FastClines* due to low numbers of sampling locations producing coarser estimates from this approach. Spatially dense sampling and testing of alternative cline models indicate that the clines at these colour loci are mostly coincident yet display stepped clines with asymmetrical tails on the left and right sides [32]. Assuming a sigmoid cline model when clines show asymmetrical patterns of introgression is known to incorrectly push cline centre estimates apart [33]. This is one limitation of the *FastClines* method as it assumes that clines have sigmoid rather than stepped shapes, suggesting some of the spread of cline centres may be an artifact. However, one case where the difference in centres appears to be real is at the loci either side of *FLA*, which is a recently described gene that interacts with *SULF* to determine yellow pigmentation [25]. Closer inspection of cline centres walking along the chromosome near the *FLA* gene revealed a step change in cline centre position directly over the gene region. The MLE cline fitting confirmed a different cline centre estimate at representative loci either side of this gene. This step change in centre estimates coincides with the breakpoint of a recombinant haplotype at the *FLA* locus which has a different phenotype [25]. Despite some of the caveats of this approach, these results highlight the value of *FastClines* and the potential for detecting course step changes in cline properties to flag potentially important genomic areas related to phenotypic differences.

### Clines narrow down the RUBIA locus that modifies magenta intensity

The *FastClines* also approach helped locate a new gene *RUB* which *F*_*ST*_ and other diversity metrics did not as readily detect. This *RUB* locus is tightly linked to *FLS*, with RNAseq showing expression of *FLS* is high in *A. m. m* var. *striatum* (yellow) and low in *A. m. m* var. *pseudomajus*. One possible explanation is the *A. m. m. var. striatum* variants at FLS diverts common substrates into flavanols, so reducing the flux into anthocyanins [34,35]. Although the overall level of differential gene expression is not as strong as seen at other loci such as *FLA* [25], this may reflect that this region controls a phenotypic difference of smaller effect on pigmentation.

Cline fitting of a polymorphic sigmoid cline using a MLE simulated annealing approach revealed a steep cline fixed (best model, *p* = 0.96) on the *A. m. m* var. *pseudomajus* (magenta) side of the hybrid zone yet the locus remains still polymorphic on the yellow side of the hybrid zone (*p* ~ 0.4) (Fig 3) (Surendranadh et al. 2025). Although the allele is close to asymptote on the far yellow side, this part of the transect is in another valley and not well connected to the rest of the hybrid zone [23]. Therefore, the *RUB* locus on the yellow side of the hybrid zone exposed to gene flow is largely polymorphic in contrast to the magenta side (i.e., excluding areas over the mountain pass). This could be due to the *RUB* allele from *A. m. m var. pseudomajus* having limited phenotypic effect on predominantly *A. m. m var. striatum* (yellow) parental genotypes and thus being unseen by selection. Future efforts to test the strength of selection of alternative alleles on either side of the hybrid zone will be important in revealing if the epistatic interaction between *RUB* on different *ROS* backgrounds translates to fitness epistasis.

## Conclusions

Our study highlights several future challenges for research in understanding genome-wide distributions of clinal loci. Firstly, the covariance of cline parameters due to tight linkage in *Antirrhinum* highlights the need for future theoretical approaches that account for the non-independence among loci. Unlike classical hybrid zone studies using small numbers of markers, the move to whole genomes necessarily results in loci in high LD many loci will not be statistically independent. This is also an issue for other genomic cline methods, which are based on detecting outliers from the neutral background loci, which are wrongly presumed independent. One approach to dealing with this problem is to move beyond the analysis of single loci to haplotype blocks [36]. Secondly, we still lack a theoretical understanding of the expected distribution of selected loci across genome-wide data and how the rate of false positives depends on the time since secondary contact, drift, local recombination rate and the strength of selection. The much higher frequency of clinal loci around the *FLA* gene (region of low recombination), compared with *ROS* and *EL* (high recombination), highlights the importance of the recombination landscape and why the density of clines in a region is not a good indication of the importance of the region as a barrier. This issue is similar to the elevated *F*_*ST*_ associated with regions of the genome with lower rates of recombination and nucleotide diversity [37]. Regardless of the evolutionary forces responsible for the signal, more clines may be expected in areas with high sequence divergence, low diversity and lower rates of recombination and these features need to be considered when interpreting the density of clines along chromosomes. Determining how far genetic barriers persist at neutral sites around a selected locus that factors in these genomic features will also provide insight into the processes structuring genome wide divergence. This will enable the further dissection of the role of stochastic, historical and contemporary forces in driving patterns of divergence and clines across genomes.

## Materials and methods

### *Antirrhinum* hybrid zone sampling

In order to conduct genome scans and estimate geographic clines with *FastClines*, we subsampled individuals from six sampling locations along a 1-dimensional transect at the hybrid zone near Planoles, Spain (Fig 2). The six sampling localities were arrayed along a 1-D transect spanning ~24 km [following 24], with tighter spacing of localities near the centre of the phenotypic cline to capture the steep transition. To ensure spatial coverage of the flower colour cline, three subpopulations were selected in the predominately yellow regions west of the centre of the cline and three in magenta dominated regions to the east (see Fig 1 and S1 Table for locations). In the outermost populations (YP4 and MP11) only yellow and only magenta individuals are present, respectively. However, in the remaining (YP2, YP1, MP1, MP4) hybrids and both parental phenotypes are present (Fig 1). Altitude gradually increases up the valley going West, above ~1600 metres in altitude, *Antirrhinum* is absent. Thus, a break of ~4 kilometres in the distribution of *Antirrhinum* plants coincides with a mountain pass (~3–7km along transect). The outermost yellow population (YP4) is situated west of this pass, while the other five population samples are east of the mountain pass (Fig 1).

Within the bounding polygon of the georeferences of all available tissues, six points were chosen with appropriate spacing, and such that 50 individuals could be subsampled within 50 meters of each point. In this larger project, over 30,000 plants were located to within 3.4 metres with a GPS (Trimble GeoXT datalogger), leaf tissue collected for DNA extraction and one flower taken for phenotyping [see details in 38]. Following Whibley et al., [22], individuals were categorized into six phenotype/genotype classes on the basis of anthocyanin and aurone pigmentation across the flowers.

### Whole genome sequencing and KASP SNP genotying

We sequenced the six pools and used whole genome sequencing (n = 50 individuals in each pool) carried out using Illumina HiSeq. The DNA extraction methods, sequencing and bioinformatic pipelines and filtering are described in detail previously [23], with the exception that the original data has been re-mapped to *Antirrhinum* reference genome v3.0 [28,29].

The larger sample of individuals (n = 30,000) were also SNP genotyped with Kompetitive Allele Specific PCR (KASP) [following 39] as part of a separate pedigree project. Here, we used this KASP SNP genotype dataset for genotype-phenotype associations (see below) and to fit clines and verify *FastCline*s estimates from poolSeq. Candidate loci for the KASP marker design were identified from the poolSeq data including loci for (i) each gene known to influence flower colour (*ROS*, *EL*, *SULF*, *FLA, CRE*), (ii) major cline clusters identified (using *FastClines*) on each chromosome (including new colour loci *RUB*)(see S2 Table for marker details). All genotyping and scoring was carried out by LGC genomics. The SNP KASP genotyping data and poolSeq population pool information is available on Dryad data repository [39].

### Genome wide estimation of geographic clines properties using *FastClines*

Estimating geographic cline parameters for whole-genome data is not straightforward, especially when alleles are not fixed for alternative alleles in the outer most populations ($\Delta p_{\text{1},\text{6}}\neq \text{1}.\text{0})\;$when many parameters need to be fitted for thousands of loci across whole genomes. One approach is the cline approximation method described by Polechova & Barton [30], which is not computationally demanding and provides a reasonable approximation to detailed cline model fitting. They showed that the width of a single locus logistic cline can be approximated for diagnostic loci as the integral of heterozygote frequencies over space as $w=\text{4}\int_{-\infty }^{\infty }pq\;dx,\;$ which equals the inverse of the maximum gradient $w_{max}$. For discrete demes, this is essentially twice the sum in the frequency of heterozygotes across all demes,


$$\begin{array}{c} w=\text{4}\sum_{i}^{n_{d}}p_{i}q_{i} \end{array}$$

where, *n*_*d*_ is the number of demes, *p*_*i*_ and *q*_*i*_ the allele frequencies in the *i*th deme, and width is measured in deme spacing. Similarly, the centre of the cline can be estimated as the sum of allele frequencies adjusted by half a deme,


$$\begin{array}{c} =n_{d}-\sum_{i}^{n_{d}}p_{i}+\text{0}.\text{5} \end{array}$$

However, with real data loci may not be fixed for alternative alleles in one or both parental populations. Moreover, this model assumes equal spacing among demes (i.e., continuous sampling locations).

We developed a new method we call *FastClines*, which extends this approach to non-diagnostic loci that also accounts for differences in deme spacing (size) across a hybrid zone (see Fig 1 for example). For diagnostic or non-diagnostic loci, we denote the allele frequencies in the two parental populations (or outer most sampling localities, or demes when continuous) as *p*_0_ and *p*_1_ and assume that they are known. We then restrict the integral to lie within the interval of the allele frequency differences between the parental populations for each deme as $\left(p_{i}-p_{\text{0}}\right)\left(p_{\text{1}}-p_{i}\right)$. The cline width can then be approximated by,


$$\begin{array}{c} w=\text{4}\sum_{i=\text{1}}^{n_{d}}\frac{\left(p_{i}-p_{\text{0}}\right)\left(p_{\text{1}}-p_{i}\right)}{{\left(p_{\text{1}}-p_{\text{0}}\right)}^{\text{2}}}d_{i} \end{array}$$

where *d*_*i*_ is the span of the sampling location, and equal to half the Euclidean distance between the midpoint of the samples in each locality. For the outermost localities we extend this outwards to the same distance. This term *d*_*i*_ provides the appropriate scaling to account for irregular spacing of localities (see S1 Text for example).

Similarly, cline centre is approximated by,

$c=\sum_{i=\text{1}}^{n_{d}}d_{i}-\sum_{i=\text{1}}^{n_{d}}\frac{\left(p_{i}-p_{\text{0}}\right)}{\left(p_{\text{1}}-p_{\text{0}}\right)}d_{i}$

where the first term orientates the centre with respect to *p*_0_.

This cumulative heterozygosity (i.e., 4∫pqdx) provides a consistent measure for any cline shape, yet its exact correspondence to cline width, as measured by the inverse of maximum slope, depends on the form of selection [30]. Specifically, 4∫pq dx equals the inverse of the maximum slope when selection results from heterozygote disadvantage. However, when environmental selection shifts abruptly from +s to -s, the inverse of the maximum slope equals 3∫pq dx [30].

We use this *FastClines* method on the *Antirrhinum* data, using the pooled allele frequencies in the six sampling locations across the hybrid zone. Here the localities were irregularly spaced apart and we scaled according to the midpoint distance between the locality mid-point [*d*_*i*_ = (6000,5000,1500,1400,3500,6000); S1 Table] along a transect which was set from the Maximum Likelihood Estimation (MLE) cline fitting (see below). We include only loci with strong allele frequency differences between the outer pools Δp≥ 0.90. To account for rare allele frequency reversals generating negative cline widths (an artefact from limited spatial resolution; S1 Text), we also implemented an adjusted parental allele frequency (for *p*_*0*_ and *p*_*1*_) by using the minimum and maximum allele frequencies on the left and right side of the centre. More specifically, we define an adjusted parental allele frequency as the minimum allele frequency for the parental allele frequency on the left flank as, *p*_0 adj =_ Min(p_1,_ p_i_) and on the right flank parental allele frequency as, *p*_1 adj =_ Min(p_1,_ p_i_). All estimates were calculated in a custom Python script *FastClines* for cline approximations for whole genome data (https://github.com/dfield007/fastClines).

To demonstrate the speed of this new approach, we ran some benchmark tests to compare *FastClines* to MLE cline fitting (see below), running both approaches on a MacBook Pro (2023 model) with 12 core Apple M2 Max chip with 64GB RAM. Running *FastClines* on the *Antirrhinum* poolSeq data with 6 localities used in this manuscript, when searching through whole genome sync files, filtering and calculating cline width and centre, it took ~0.0004 seconds per site (locus), and ~7 minutes for 1 Million sites. In comparison, using our MLE approach (https://github.com/dfield007/genome_wide_clines) used in the manuscript takes ~25 seconds per locus (for modest number of iterations = 20000 and burn-in = 5000), therefore if we had more KASP loci available (to make more comparable to our poolSeq data), this would be ~ 34 hours for 5,000 loci and ~6800 hours (=283 days) for 1 million SNP loci.

### Maximum likelihood cline fitting with simulated annealing

We fitted geographic clines to SNP genotypes using MLE fitting to compare with *FastClines* estimates from poolSeq. We settled on 200m sampling location radius, this scale minimized deficits from Hardy-Weinberg Equilibrium (HWE) whilst ensuring sufficient sample size within localities (mean = 40, sd = 20, range 10–200) and yielded ~35,000 individuals across ~400 localities across the hybrid zone (i.e., numbers vary slightly across loci). Only one marker within *ROS* displayed significant departures from HWE with a deficit of heterozygotes (*F* > 0) even at small spatial scales (<50 meters). Therefore, we expect based on theory that this feature may generate narrower cline widths than expected.

The clines were characterised for each colour locus, using a modified version of a custom R script (https://github.com/dfield007/slowClines) described previously [23,24]. This script uses fits a symmetric polymorphic sigmoid cline with five parameters:


$$\begin{array}{c} \hat{p}=\frac{p_{\text{0}}+\left(p_{\text{1}}-p_{\text{0}}\right)}{\text{1}+\text{exp}\left(-\text{4}\left(\frac{x-c}{w}\right)\right)} \end{array}$$

Where c = cline centre, w = cline width (1/gradient), $p_{\text{0}}$ = allele frequency at the asymptote in the west (*A.m. m. var striatum* parental allele frequency) and $p_{\text{1}}$ = allele frequency at the asymptote in the east (*A.m. m. var pseudomajus* parental allele frequency) and $F_{ST}=var(p)/\overset{―}{p}\left(\text{1}-\overset{―}{p}\right)$. For the $F_{ST}$ parameter, we fitted a beta-binomial error term to account for the variance in allele frequencies across sampling locations and to control for population structure along the cline and provide a better model fit. Prior to commencing cline fitting, the larger data set was randomly thinned within each locality to reduce the computational time, and filtered to remove localities with less than 10 individuals, resulting in 10,000 individual genotypes across ~120 localities.

We use a metropolis-hastings (simulated annealing) algorithm to sample the likelihood surface of the cline fit. We begin the algorithm with randomly chosen parameter values and the log likelihood *logL* is computed at each iteration. When the next iteration *logL”* has a greater log likelihood than the previous likelihood *logL’* (i.e. *logL”* > *logL’*), the new parameters are accepted. If the next iteration is lower (*logL”* < *logL’*), we accept with a probability *logL”*/*logL.’* To ensure ample exploration of the likelihood surface, the jump size for the next set of parameters are adjusted by a factor of 1.05 when accepted (accept scale) and by (1/1.05) when parameters are rejected (reject scale). After some tests of different accept and rejection scales, we found these values achieved efficient mixing and exploration of the likelihood surface with an acceptance rate ~0.5. This algorithm was run for 50,000 iterations with a burn-in = 2000. From this we find the most likely cline parameters, maximum logL and assume the likelihood surface follows a chi-square distribution to find -2 logL max 95% credible regions. We visually inspected the joint likelihood surface for each run. Each run was repeated with randomly chosen starting parameters to ensure reproducibility. Different cline transect orientations were tested (S3 and S4 Figs). More detailed cline fitting of alternative cline shapes and asymmetries combined with estimates of selection are being address elsewhere.

### Simulations of cline estimates from *FastClines*

We used simulations to examine the power of the *FastClines* approximation method to distinguish coarse shifts in the centre and width of clines. Rather than forward simulating the dynamics of hybrid zones through generations following dispersal and selection acting on loci [e.g., 17], this simulation assumes smooth clines with known properties are already in place at a particular fixed time point. The position and width of clines is expected to vary from the true parameters due to the demographic process (e.g., drift that affects allele frequencies, non-random mating) and sampling effects (number of sampling locations, sampling error, variation in sequencing depth and sequencing error). We modeled each of these factors using a similar setup to the whole genome data for *Antirrhinum* and explored other arrangements with more sampled localities.

The setup follows a 1-dimensional landscape with a symmetric polymorphic sigmoid cline with five parameters (see cline model above). For simplicity, the total transect length matched the *Antirrhinum* hybrid zone (sampling locality positions from 0 to 24km). The simulation begins by generating a matrix of allele frequencies in sampling localities along columns (locations along the cline transect) with replicate sampling along rows. For each locality, observed allele frequencies are drawn from a beta distribution where alpha = (1/ *F*_ST_)-1). In each locality, the expected allele frequency is based on the cline parameters yet the observed values fluctuate, first due to drift following a Beta distribution,


$$\begin{array}{c} p_{sim}=Beta\left[\left(\frac{\text{1}}{F_{ST}}-\text{1}\right)\hat{p},\left(\frac{\text{1}}{F_{ST}}-\text{1}\right)\left(\text{1}-\hat{p}\right)\right] \end{array}$$

where *F*_ST_ = 0.03 (following estimates at neutral loci for *Antirrhinum*)*.* Next, a specified number of individuals to represent sampling from field sites as is each locality were drawn for each of the possible homozygote diploid genotypes (*g*_*kk*_, *g*_*jj*_) and heterozygote (*g*_*kj*_). The diploid genotypes were randomly drawn from the observed allele frequencies ($\hat{p}$) following a binomial and departures from random mating expectations at Hardy-Weinberg equilibrium (HW) modeled by varying the *F* parameter below for each of three genotypes as,


$$\begin{array}{c} P(g_{kk})={\left(\text{1}-\hat{p}\right)}^{\text{2}}+\hat{p}\left(\text{1}-\hat{p}\right)F \end{array}$$

$$\begin{array}{c} P(g_{kj})=\text{2}\hat{p}\left(\text{1}-\hat{p}\right)(\text{1}-F) \end{array}$$

$$\begin{array}{c} P(g_{jj})={\left(\hat{p}\right)}^{\text{2}}+\hat{p}\left(\text{1}-\hat{p}\right)F \end{array}$$

To simulate variation in sequencing depth, we used a normal distribution with a mean and variance calculated from the poolSeq data. The method draws sequencing reads (with replacement) from the alleles contained in the diploid genotypes in each locality. We used a normal distribution with a mean and variance following the observed values in the *Antirrhinum* aligned whole genome data with sequencing errors introduced at 1 x 10^-4^. For each set of parameter values we ran replicate simulations (*n* = 10,000) to examine the variation in cline width and centre estimate.

We first explored parameter space with a combination of width and number of sampling locations to test how cline width estimation is improved with density of geographic sampling [w={2,4,6,8}, localities={6, 8, 10, 12}]. We also tested varying both width and centre values [w={2,4,6,8}, centre = {8, 10, 12, 14, 16}]. To examine the effect of departures from HWE and variance in allele frequencies due to structure, we ran simulations for one set of cline parameters (c = 450, w = 150) for a range of *F* = {0,0.5,0.1,0.2} and *F*_ST_ = {0.01, 0.05, 0.1}. Similarly, to examine the effect of increased sequencing depth, we also ran this set of cline parameters for a range of depth (25, 50, 100) with variance similar to observed values $\left(\sigma^{\text{2}}=\text{5}\right)$. Scripts for cline simulations were written in R and are available online (https://github.com/dfield007/fastClines).

### Genomic diversity and divergence

We quantified relative genetic divergence and diversity within and between the six pooled populations within 10kb sliding windows using the methods outlined in Tavares et al., (2018) with a few modifications. These included remapping the data to the *Antirrhinum* reference genome v3.5 and running through the custom *SlidingWindows* script v1.14 (https://github.com/dfield007/slidingWindows). We estimated diversity within (π_*w*_) each of the populations, and total diversity (π_*t*_), relative (*F*_ST_) and absolute differentiation (*D*_*xy*_) between each pair of populations using approaches outlined previously [23].

### Annotation of genes corresponding to gene clusters

To determine whether the identified clines correspond to phenotypic effects, we annotated protein-coding genes from major cline clusters on each chromosome (top-ranked cline proportions; Fig 5c). We first extracted cluster-associated genes from the genome annotation file (reference genome version 3.5). We performed a functional annotation using the eggNOG-mapper v2 [40], combining orthology assignments, functional descriptions, and gene ontology (GO) terms. Simultaneously, the genes were subjected to similarity searches using the BLAST online tool (Basic Local Alignment Search Tool) against the NCBI non-redundant (nr) database. The functional information and gene hits obtained from both eggNOG-mapper and BLAST were manually curated to ensure accuracy and relevance. The location of all known genes that influence flower colour was combined with genes identified around clinal loci. Genes were then categorized into functional groups, including the broad term ‘colour related gene’ to include all of those involved in the flavonol biosynthetic pathway or known to regulate the expression (intensity or distribution) of colour pigments across parts of the flower in *Antirrhinum majus*.

### Clustering in the genome

We used permutation tests to determine if clinal loci were more clustered in the genome than expected by chance. We randomized the labels associated with all SNP positions (i.e., clinal SNP = Y or N) in the genome and calculated the mean distance in bp between all possible pairs of clinal labels. This was repeated 9,999 times, and each time the permuted mean pairwise distance was retained to generate a null distribution. The observed value of the main pairwise distance was calculated for the observed data. We calculated a p-value for the observed value as the number of permuted datasets where the mean pairwise distance was equal to or greater than the observed estimate + 1/number of permutations +1.

### Genotype-phenotype associations at cline cluster chromosome 5

To examine the effect of genomic region on Chr 5 (Chr5CC = *RUBIA*) which gene annotation discovered was in close proximity to Flavanol Synthase (*FLS*) we examine genotype-phenotype associations in controlled crosses and wild plants from the hybrid zone. First, we generated an F2 cross between purebred *A. m. m var. pseudomajus* (magenta flowers) and *A. m. m var striatum* (yellow) from outside the hybrid zone. A sample of 69 individuals were SNP genotyped using KASP to confirm genotypes at known colour loci and the clinal SNPs on Chr 5 (Chr5CC). Flower colour ranking results for an *F*_*2*_ hybrid population segregating for Chr5CC and their genotypes at colour loci *ROS*, *EL*, and *SULF*, grouped by shared genetic backgrounds. Photographs from different groups were then separately ranked according to magenta intensity, yielding a low magenta bin and a high magenta bin. Counts of the *A. m. m. pseudomajus* (ps) and *A. m. m. striatum* (st) Chr5CC homozygotes, summed across all genotypic groups. Secondly, we generated *F*_*3*_ and then *F*_*4*_ crosses and genotyped 204 individuals for each of the colour loci and the marker tagging Chr5CC. Similarly, photographs were ranked according to magenta intensity, yielding four quartiles of increasing magenta. Counts of the *A. m. m. pseudomajus* (ps) and *A. m. m. striatum* (st) Chr5CC homozygotes and heterozygotes (het) were compared. Lastly, to test whether *RUBIA* effects magenta intensity on yellow backgrounds, we genotyped and isolated parental genotypes of *A. m. m. striatum* (*ros*^*st*^ *El*^*st*^*/ros*^*st*^ *El*^*st*^) that were segregating for *RUB* alleles (n = 105 individuals) and similarly ranked flowers for magenta intensity within each multi-locus genotype class. All statistical tests of significance in the counts of alleles in each cross were conducted using a chi-squared test implemented in R (see further details S4 Text).

In addition, we phenotyped and genotyped plants from the hybrid zone to examine the effect of clinal loci on Chr 5 on flower colour. In the hybrid zone, we randomly selected a subset of the six major colour phenotypes: Magenta, Yellow, Pink, Weak Orange, Full Orange and White [following 22] that has been genotyped with KASP. Using photographs of the front of the flower color measurements were taken in ImageJ ([href:http://imagej.nih.gov/ij/]http://imagej.nih.gov/ij/) at six standardized locations on the flower (S15 Fig). For each region of the flower, we calculated the mean and standard deviation of Hue, Saturation (Sat), Intensity (Int) and Greyscale (GS) and transformed these values to HSV colour space (Hue, Saturation and Value). The effects of *ROS1* and *RUB* genotypes on colour phenotypes were assessed with linear regressions, with interaction effects included to test for epistasis (see full details S5 Text). The flower colour phenotyping is available on Dryad data repository [39]

### Differential gene expression

RNA was extracted from corolla tissue from snapdragon flowers for three biological replicates of a representative *A. m. m* var. *pseudomajus* (magenta flowers) and *A. m. m* var. *striatum* (yellow). We used the annotated *Antirrhinum* reference (Accession number GWHBJVT00000000) genome v3.5 ([href:http://bioinfo.sibs.ac.cn/Am/]http://bioinfo.sibs.ac.cn/Am/) and added “decoy-aware” index for the mapping. For each of the samples we next used Salmon to align and quantify transcript abundance to our annotated genome, including flags to increase the stringency of the mappings (--validateMappings) and to learn and apply corrections for GC bias and primer bias (--gcBias --seqBias). We then performed differential gene expression analyses using Wald test hypothesis in DESeq2 [41](implemented in Bioconductor v3.2 in R) with the lfcShrink function to compensate for inflated log2fold changes in genes that have low counts (after filtering out genes with < 10 reads). As a reference point, other known colour genes in the top clusters on Chr 6 (*ROS*/*EL*) and Chr 2 (*FLA*) we found to have the highest outliers for differential gene expression (see full details S6 Text).

## Supporting information

S1 Text *FastClines* setup. (DOCX)

S2 Text Maximum Likelihood Estimation cline fitting. (DOCX)

S3 Text Clinal loci gene identification and enrichment. (DOCX)

S4 Text Colour associations in controlled crosses. (DOCX)

S5 Text Colour associations in hybrid zone plants. (DOCX)

S6 Text RNA seq and differential gene expression analysis. (DOCX)

S1 Table Geographic locations of whole genome pools across *Antirrhinum* hybrid zone. (DOCX)

S2 Table KASP SNP genotype marker details. (DOCX)

S3 Table MLE cline parameters for best fitting model of KASP SNP genotypes. (DOCX)

S4 Table Test for greater clustering of divergent loci showing clines on each LG (Chromosome) than expected by chance. (DOCX)

S5 Table Test for enriched overlap of clinal windows and *F*_*ST*_ outliers. (DOCX)

S6 Table Test for differences in *π, d*_*xy*_ and *F*_*ST*_ between clinal and non-clinal windows between population pairs. (DOCX)

S7 Table Linear regressions of genotype variation at *ROS* and *RUB* loci and quantitative flower colour scores for Hue in HSV colour space. (DOCX)

S1 Fig Simulations of *FastClines* estimates with varying cline widths and number of sampling localities for loci with fixed differences and high sequencing depth. (TIFF)

S2 Fig Simulations of *FastClines* estimates of cline width and centre for loci with AFD = 0.95. (TIFF)

S3 Fig Maximum log Likelihood (maxLL) for each gradient parameter search at each transect directions. (TIFF)

S4 Fig Allele frequencies from KASP SNP genotyping at the *ROS1* locus in 200m sampling locality radius (200m) along the hybrid zone transect. (TIFF)

S5 Fig Allele frequencies for clinal loci around the *FLA* gene on Chromosome 2. (TIFF)

S6 Fig Allele frequencies for clinal loci downstream of *FLA* gene (>300kb) on Chromosome 2. (TIFF)

S7 Fig Allele frequencies for clinal loci around the *CRE* gene on Chromosome 1. (TIFF)

S8 Fig Allele frequencies for clinal loci around the *SULF* gene on Chromosome 4. (TIFF)

S9 Fig Allele frequencies for clinal loci around the *RUB* gene on Chromosome 5. (TIFF)

S10 Fig Allele frequencies for clinal loci around the *ROS*/*EL* genes on Chromosome 6. (TIFF)

S11 Fig Allele frequencies for clinal loci around on Chromosome 3, 7, and 8. (TIFF)

S12 Fig Cline width and centre for different filtering for loci. (TIFF)

S13 Fig Allele frequencies across the transect for 16 loci with negative cline widths. (TIFF)

S14 Fig Number of outlier 10kb windows for 95% quantile for all pair-wise population comparisons of *F*_*ST*_ in colour genes and linked regions along the genome. (TIFF)

S15 Fig An example of the six regions of the front view of an *Antirrhinum* flower used to quantify patterns of floral pigmentation. (TIFF)

S16 Fig Summary of HSV Hue scores of *Antirrhinum* flowers for *ROS* and *RUB* haplotypes from the hybrid zone. (TIFF)

S17 Fig Summary of HSV Saturation scores of *Antirrhinum* flowers for *ROS* and *RUB* haplotypes from the hybrid zone. (DOCX)

S18 Fig Summary of HSV Value scores of *Antirrhinum* flowers for *ROS* and *RUB* haplotypes from the hybrid zone. (DOCX)
