# Supplementary material for: Genome-wide cline analysis identifies new locus contributing to a barrier to gene flow across an Antirrhinum hybrid zone
Source: PLoS Genet. 2026 Jul 13;22(7):e1012173. doi: 10.1371/journal.pgen.1012173 (PMC13387609; doi:10.1371/journal.pgen.1012173)
Supplement: S1 Text — (DOCX) [file pgen.1012173.s001.docx]

## **S1 Text. FastClines setup**

*Deme locations along transect*

We used the whole genome poolSeq data and the transect positioning through the hybrid zone as described in Tavares et al., 2018. The geographic positions of each pool were located along this transect and collapsed to one dimensional geographic distance (in kilometers) along the transect (Fig 1). The total geographic distance along the transect was set by the larger SNP KASP genotype data with demes located from 0 km to 25.176km. For the poolSeq, the deme location was set as the average Easting and Northing of individuals in each pool (Table S1) with positions then collapsed along the 1-dimensional transect to the following positions [YP4 pool 1: 0.382km, YP1 pool2: 11.227, YP2 pool3: 11.404, MP4 pool4: 13.767, MP4 pool5: 14.424, MP1 pool6: 20.670km].

*Deme spans*

In the fastClines method, we need to define the geographic span of each deme ($d_{i}$ and see Fig 2). If the demes are equally spaced across a transect, one approach is to use half the distance either side of the geographic centre of each deme as the span of each deme. However, the patchiness of individual samples in natural populations, including the snapdragon system, often preclude setting equal distances between pools. Therefore, to account for possible influence of deme span selection on cline properties, we used the midpoint edges: where the outer edges (pool 1 and 6) were defined by the dimensions of the KASP sample demes (0 to 25.176km) and all internal edges set as the midpoint distances between each pool along the transect [deme edges: 0, 5.423, 11.316, 12.586, 14.095, 17.548, 25.172km] from which the geographic span of each deme was calculated [pool 1: 5.805km, pool2: 5.893, pool3: 1.270, pool4: 1.500, pool5: 3.453, pool6: 7.251km]. The total span of the demes was 25.172km and the midpoint between pool 3 and 4 (transition across centre of phenotype cline and ROS1 gene) was fixed at 12.586km. This allowed for comparisons of cline centres and widths relative to the known position of the ROSEA locus (~12.5km – 13.5km) and the main phenotypic cline.

**Filtering and cline estimates for *Antirrhinum***

We use this method on the *Antirrhinum* data, taking the pooled allele frequencies as given across the hybrid zone. Here the demes were irregularly spaced apart and we scaled according to the midpoint distance between the demes [*d_i_* = (5800,5890,1200,1500,3450,7200)]*.* Although allele frequencies estimate may benefit from incorporating errors for pooled data, we found a high correlation between allele frequencies estimated from the pools and those estimated from individual genotyping of the same 50 individuals in each pool [1]. We include only loci with strong allele frequency differences between the outer pools $\Delta p_{1,6}$≥ 0.80 and $\Delta p_{1,6}$≥ 0.90. For these loci, allele frequencies were polarized so that they increased from West to East (yellow to magenta, respectively). One limitation of this method is that cline reversals (e.g. *p*_5_ *> p*_6_) for non-diagnostic loci can result in negative cline widths when no adjustment is used for *p*_0_ and *p*_1_ (see below).

Initial analysis using *FastClines* found n = 12,936 clinal loci with a low depth filtering threshold and allele frequency difference between the outer most pools (min depth x = 10 in at least 5 of 6 pools, $\Delta p_{1,6}$≥ 0.80). This was reduced to n = 10,912 and n = 7,271 loci when increasing filtering to a minimum of 15 and 20 depth, respectively (in at least 5 of 6 pools). With the non-adjusted parental allele frequencies (*p*_0_ and *p*_1_), negative cline widths were detected at n = 299 loci of 12,936 (2.3%), n = 249 loci of 10,912 (2.3%) and n = 162 loci of 7,271 (2.2%) loci when filtering for minimum sequencing depths of 10, 15 and 20, respectively [Figure S13(a -e)]. The majority of loci with negative cline widths were positioned with centres at the extreme ends of the transect. For example, considering loci with at least 15 depth (S12 Fig), we found n = 232 (93.2%) were centred to the extreme left (<6000m) or right (>15,000m) of the transect. Filtering to loci with $\Delta p_{1,6}$≥ 0.90 had a stronger impact than filtering for higher depth in reducing the frequency of negative cline widths. For example, at minimum depth 15, increasing to $\Delta p_{1,6}$> 0.9 resulted in 16 loci of 3,826 (0.04%) with negative cline widths compared with 249 loci of 10,912 (2.3%) of loci for $\Delta p_{1,6}$> 0.8. Negative cline widths were observed for loci with reversals in allele frequencies along the transect. For example, when considering loci with $\Delta p_{1,6}$≥ 0.80, the n = 16 negative cline widths positioned near the hybrid zone centre (10,000 – 15,000m) tended to display allele frequency reversals in the outer first pool (S14 Fig).

We constrasted the raw parental allele frequencies, with an adjusted value to account for allele frequency reversals. Here, we define an adjusted allele frequency as the minimum allele frequency for the parental allele frequency on the left flank as, *p*_0 adj_ = Min(p_1,_ p_i_) and on the right flank parental allele frequency as *p*_1 adj_ = Min(p_1,_ p_i_). Using these adjusted parental frequencies removed the majority of negative cline widths. Using *p*_0 adj_ and *p*_1 adj,_ with $\Delta p_{1,6}$> 0.8 negative cline widths were detected at n = 27 loci of 12,936 (2.3%), n = 21 loci of 10,912 (2.3%) and n = 14 loci of 7,271 (2.2%) loci when filtering for minimum sequencing depths of 10, 15 and 20, respectively. When $\Delta p_{1,6}$was increased, there were no negative cline widths for depth of 15 and 20. In summary, these remaining negative clines represent artefacts due to the limited number of pools at lower allele frequency differences. This tends to draw widths downward as centres move to the edge of the last deme. Focusing on loci with fixed differences or greater allele frequency differences ($\Delta p$> 0.9) between populations/species is important in reducing the frequency of negative cline widths.
