# Supplementary material for: Genome-wide cline analysis identifies new locus contributing to a barrier to gene flow across an Antirrhinum hybrid zone
Source: PLoS Genet. 2026 Jul 13;22(7):e1012173. doi: 10.1371/journal.pgen.1012173 (PMC13387609; doi:10.1371/journal.pgen.1012173)
Supplement: S2 Text — (DOCX) [file pgen.1012173.s002.docx]

## **S2 Text. Maximum Likelihood Estimation cline fitting**

Prior to cline fitting of SNP KASP data for the six colour loci, the allele frequencies in demes were collapsed to one-dimension, by using a linear transect through the approximate cline centre (*p* = 0.5 isocline of ROS1). To search for the optimal transect gradient we compared cline width and maximum log Likelihood (*logL*) values with a range of gradients and intercepts centred on the p = 0.5 isocline through the valley (see S4 Fig for example) at each of the loci.

Comparing the optimal transect across loci, there was no common transect direction (gradient) with a best fit across all loci (S3 Fig). We found that a gradient of -0.345 and intercept at 6.9km generated the best compromise with highest likelihood (*logL; maxLL*) or within 2 *logL* of the maximum (maxLL) at two of the three loci (*ROS1* and *FLAVIA* up; S3 Fig). The third locus (*FLAVIA* down) was a considerably poorer cline fit at this transect, however comparing its best fitting transect cline parameters to the common well-fitting transect (at *ROS1* and *FLAVIA* up) showed similar centres and widths regardless of the transect chosen. Therefore, from hereon we report cline parameters for the gradient of -0.345 and intercept at 6.9km (S4 Fig).
