# Supplementary material for: Genome-wide cline analysis identifies new locus contributing to a barrier to gene flow across an Antirrhinum hybrid zone
Source: PLoS Genet. 2026 Jul 13;22(7):e1012173. doi: 10.1371/journal.pgen.1012173 (PMC13387609; doi:10.1371/journal.pgen.1012173)
Supplement: S3 Text — (DOCX) [file pgen.1012173.s003.docx]

## **S3 Text. Clinal loci gene identification and enrichment**

*Clines and gene classifications*

All major cline clusters on each chromosomes (top ranked cline proportions; Fig 4c) were identified from the annotation file corresponding to the reference genome version 3.5. The extracted gene sequences were formatted in FASTA and annotated using two primary bioinformatics tools. First, functional annotation was performed using the eggNOG-mapper v2, an efficient tool for fast functional annotation of novel sequences against the eggNOG database, leveraging orthology assignments, functional descriptions, and gene ontology (GO) terms [2]. The eggNOG-mapper provides comprehensive insights into gene functions, significantly facilitating the understanding of gene roles within the genome. Simultaneously, the genes were subjected to similarity searches using the BLAST online tool (Basic Local Alignment Search Tool) against the NCBI non-redundant (nr) database. The BLAST results provided additional layers of functional evidence by identifying homologous sequences and potential gene functions based on sequence similarity. The functional information and gene hits obtained from both eggNOG-mapper and BLAST were manually curated to ensure accuracy and relevance. This manual curation process involved verifying the automated annotations, assessing the significance of BLAST hits, and reconciling discrepancies between different sources of functional evidence.

The location of all known genes known to influence flower colour was combined with genes identified around clinal loci. Genes were then categorized into functional groups, including the broad term ‘colour related gene’ to include all of those involved in the flavonol biosynthetic pathway or known to regulate the expression (intensity or distribution) of colour pigments across parts of the flower in *Antirrhinum majus*. Some of these genes have been confirmed to influence phenotypic variation between *pseudomajus* and *striatum* (e.g. MYB-related transcription factors *ROSEA* and *ELUTA*). However, others have been identified through genetic screens between wild type and mutant lines in *Antirrhinum majus,* but their importance for differences between these subspecies is unknown.
