# Supplementary material for: Genome-wide cline analysis identifies new locus contributing to a barrier to gene flow across an Antirrhinum hybrid zone
Source: PLoS Genet. 2026 Jul 13;22(7):e1012173. doi: 10.1371/journal.pgen.1012173 (PMC13387609; doi:10.1371/journal.pgen.1012173)

## **S4 Text. Colour associations in controlled crosses**

**F2 – Family J109**

We analysed flower colour variation in an *F_2_* population from a cross between *A. m. striatum* and *A. m. pseudomajus*. Flowers were photographed, and photographs were grouped according to *ROS*, *EL*, and *SULF* genotypes (n = 69 flowers analysed, Fig. 7A).

**Table:** Number of plants photographed from each genotypic class used in the *F_2_* ranking analysis.

| **Group** | **Genotype** | **Number photographed** |
| --- | --- | --- |
| 1 | *ROS* *el*/*ROS* *el* *SULF*/- | 9 |
| 2 | *ROS* *el*/*ROS* *el* *sulf*/*sulf* | 5 |
| 3 | *ROS* *el*/*ros* *EL* *SULF*/- | 23 |
| 4 | *ROS* *el*/*ros* *EL* *sulf*/*sulf* | 10 |
| 5 | *ros* *EL*/*ros* *EL* *SULF*/- | 13 |
| 6 | *ros* *EL*/*ros* *EL* *sulf*/*sulf* | 9 |

For each group, we carried out three independent rankings of flowers according to magenta intensity, yielding two bins (Fig 7A). Ranked flowers were also genotyped for the SNP within Chr5CC (i.e. *RUBIA*). Pooling SNP frequencies across genotypic classes showed that the frequency of *A. m. pseudomajus* SNPs for *RUBIA* was significantly enriched in the high magenta bin, and depleted in the low magenta bin (*χ^2^* test, *p* = 1.6 x 10^-7^). These results suggest that *RUBIA* harbours a magenta flower colour locus.

**F4 – Family V159**

To further test this hypothesis, we genotyped *F_4_* populations that were homozygous at *ROS EL* and *SULF*, and repeated the ranking process (n = 204 analysed flower photos, Fig. 7B). To infer genetic dominance relationships between *A. m. pseudomajus* and *A. m. striatum* SNPs, we split the rank into four quartiles. Ranking according to magenta intensity showed a depletion of *A. m. pseudomajus RUBIA* SNPs in the low magenta quartile, and a depletion of *A. m. striatum RUBIA* SNPs in the high magenta quartile (*χ^2^* test, *p* = 4.2 x 10^-23^). This suggests that *RUBIA* expression increases magenta in *A. m. pseudomajus* flowers. The two middle quartiles were populated almost exclusively by heterozygotes showing intermediate magenta intensity, suggesting that *RUBIA* is semidominant. These results demonstrate that *RUBIA* is a novel magenta affecting locus.

Here is an example of the ranking for 80 examples:


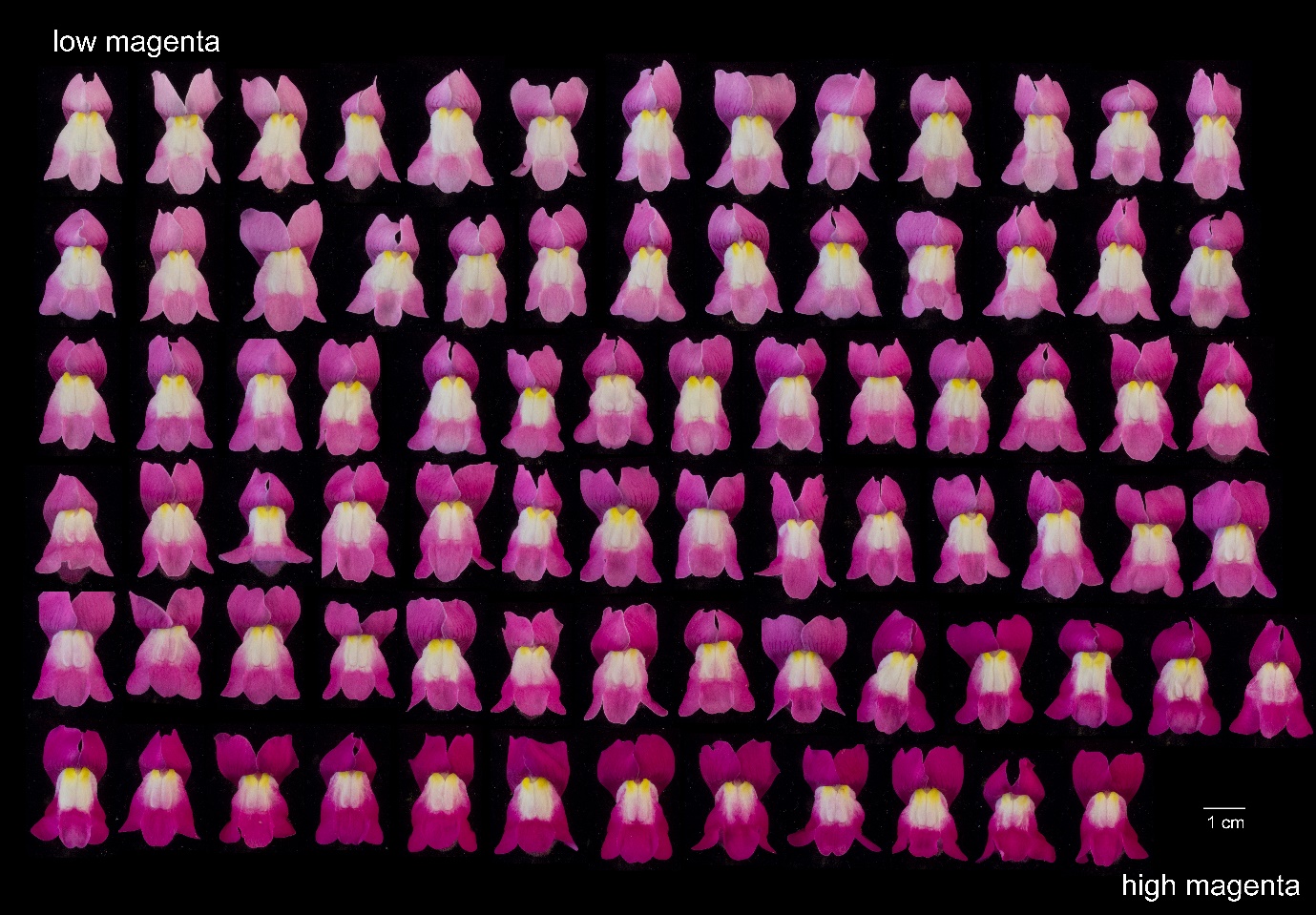


**F4 – Family Y352 (Fig. 7 C)**

*A.m.m.* var. *pseudomaju*s from HZ flank Ventola plant J1428 seed, was sown to give V163-36. *A.m.m.* var. *striatum* from HZ flank La Molina plant J1324 seed, was sown to give V206-40. V163-36 crossed to V206-40 gave F1 plants (Y132 -1 to 5) self-incompatible and so therefore sibs were intercrossed to give F2 family J109. F2 plants were intercrossed to give F3s which were again intercrossed to give F4 family Y352 (*ros^s^ EL^s^/ros^s^ EL^s^; sulf^s^/sulf^s^*) segregating for RUB^s^ and rub^p^, *FLA^s^* and *fla^p^*, *AUN^s^* and *aun^p^,* *cre^s^* and *CRE^p^*. The ranking process was repeated as above (n = 105 analysed flower photos, Fig. 7C).

Here is an example of the ranking:


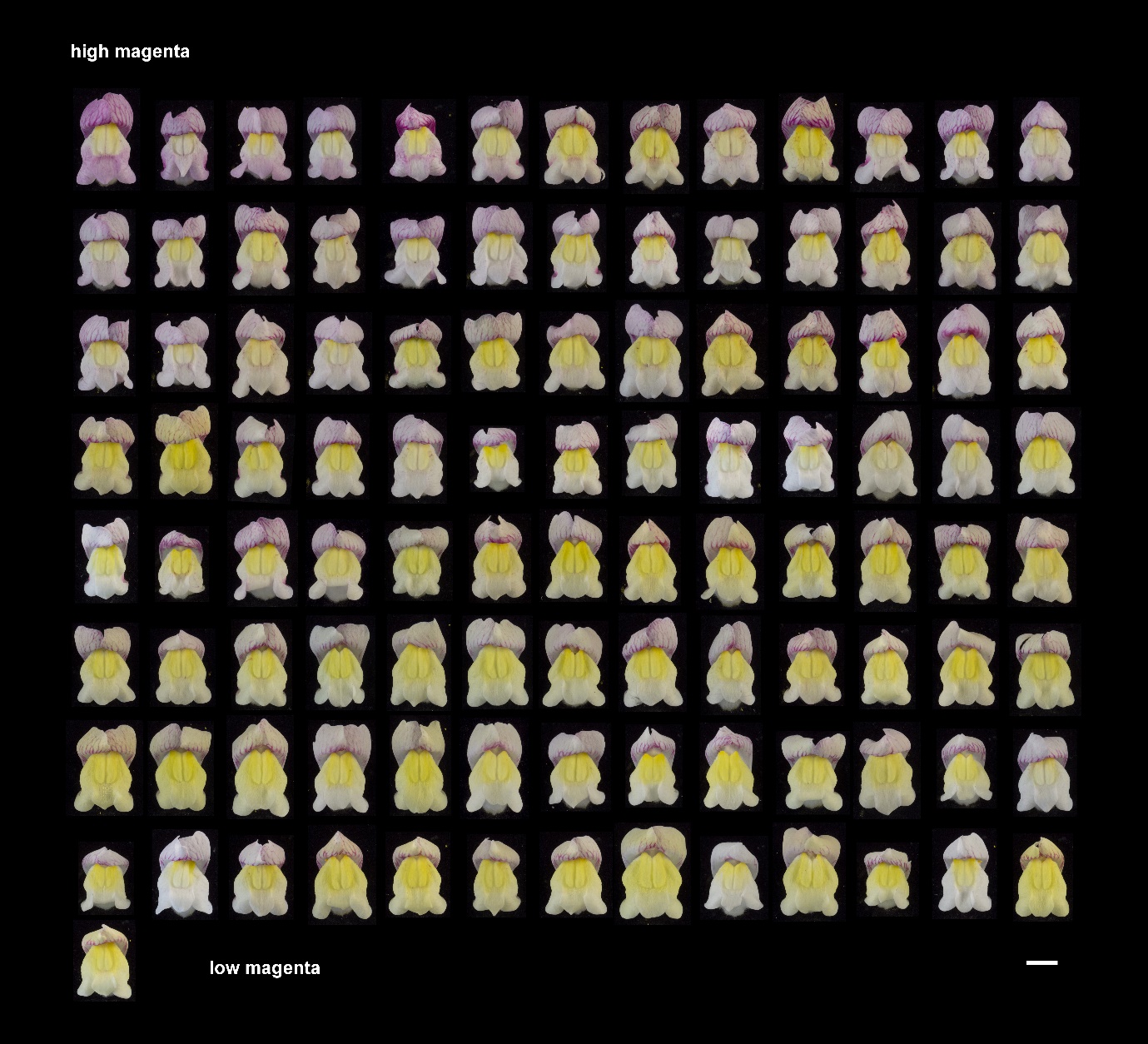

Supplement: S4 Text — (DOCX) [file pgen.1012173.s004.docx]
