# Supplementary material for: Genome-wide cline analysis identifies new locus contributing to a barrier to gene flow across an Antirrhinum hybrid zone
Source: PLoS Genet. 2026 Jul 13;22(7):e1012173. doi: 10.1371/journal.pgen.1012173 (PMC13387609; doi:10.1371/journal.pgen.1012173)
Supplement: S5 Text — (DOCX) [file pgen.1012173.s005.docx]

## **S5 Text. Colour associations in hybrid zone plants**

We phenotyped and genotyped plants from the hybrid zone to examine the effect of clinal loci on flower colour. In the hybrid zone, we randomly selected a subset of the larger KASP genotyped individuals (see above), photographs were taken on a black background with a colour standard. To perform more detailed colour quantification on a subset of plants, we stratified the random samples from the six major colour phenotypes (Magenta, Yellow, Pink, Weak Orange, Full Orange and White; following Whibley et al., (2006)), to ensure similar numbers of the main phenotype classes were available for genotype-phenotype associations.

Flower color measurements were taken in ImageJ (http://imagej.nih.gov/ij/). Images were white balanced using the macro `Chart_White_Balance` (https://imagejdocu.list.lu/plugin/color/chart_white_balance/start) which operated consistently across flowers with the color chart contained in the flower photos. To properly white balance the photos, a line was drawn from the whitest part of the white swath from the upper color chart to the darkest part of the dark swath of the upper color chart. The macro then operated and white-balanced the photo. Measurements were taken from each of six flower measurement location sites. These measurements were taken using a standard circle with an area of 5480 pixels. The circle was placed in six standard locations (S15 Fig) using the plugin “RGB Measure” to obtain individual mean and standard deviation for the Red, Green, and Blue channels.

Hue was calculated using these three colour channels. We first convert all RGB measures to 0-1 scale, then calculate Hue depending on the maximum channel as (i) Red Max: Hue =[(G-B)/(max-min)]x60, (ii) Green Max: Hue= [2+(B-R)/(max-min)]x60, (iii) Blue Max: Hue= [4+[R-G]/(max-min)]x60. As a measure of colourfullness of each region in proportion to its brightness, we calculated Saturation (Sat) as Sat = [(max-min)/255]/[1-(2L-1)], where L= [0.5(max-min)/255] and max and min refer to the maximum and minimum value amongst Red, Green and Blue channels (rescaled between 0 and 1). We also calculated the standard deviation of the Grayscale (sd_GS), using the average of standard deviation values of Red, Green and Blue channels simply as GS_sd = (sd_R+sd_G+sd_B)/3. We next calculated intensity density (Int) averaged over the three channels as Int = (R+G+B)/3. Lastly, we converted the RGB colour scheme to HSV space. This was calculated first for Hue by rescaling to the 0-1 range, keeping Saturation (S) as for RGB and calculating Value (V) as the max intensity of the three channels.

To examine the association between genotypes at *RUBIA* and colour scores we use linear regression. First we linearise Hue scores (to account for 360^0^ wrparound effects of Hue scale), by by calculating the median Hue, and using the linear distance to the median to convert to linear scale. We then fit a linear model which included the effects of each RUB and ROS1 and their interaction effects (RUB + ROS + ROS:RUB) using R (e.g. lm(H_4 ~ RUB+ ROS1 + RUB:ROS1, data = colourData). These models were repeated for each colour region, for Hue, Saturation and Value scores. An ANOVA was used to compare different models, showing that linear regressions with interaction effects fitted better than with no interactions effects (*p* < 0.05).
