# Supplementary material for: Genome-wide cline analysis identifies new locus contributing to a barrier to gene flow across an Antirrhinum hybrid zone
Source: PLoS Genet. 2026 Jul 13;22(7):e1012173. doi: 10.1371/journal.pgen.1012173 (PMC13387609; doi:10.1371/journal.pgen.1012173)
Supplement: S6 Text — (DOCX) [file pgen.1012173.s006.docx]

## **S6 Text. RNA seq and differential gene expression analysis**

RNA was extracted from corolla tissue from snapdragon flowers for three biological replicates of a representative *A. majus ssp pseudomajus* (magenta flowers) and *A. majus ssp striatum* (yellow) [3]. We used the annotated *Antirrhinum* reference genome v3.0 and added "decoy-aware" index for the mapping. For each of the samples we next used Salmon [4] to align and quantify transcript abundance, including flags to increase the stringency of the mappings (--validateMappings) and to learn and apply corrections for GC bias and primer bias (--gcBias --seqBias). We then performed differential gene expression analyses using DESeq2 (Bioconductor)[5] with the lfcShrink function to compensate for inflated log2fold changes in genes that have low counts (after filtering out genes with < 10 reads). As a reference point, we checked known colour genes in the top clusters on Chr 6 (ROS/EL) and Chr 2 (FLA) and found as expected high differential gene expression.
