# Supplementary material for: Genome-wide cline analysis identifies new locus contributing to a barrier to gene flow across an Antirrhinum hybrid zone
Source: PLoS Genet. 2026 Jul 13;22(7):e1012173. doi: 10.1371/journal.pgen.1012173 (PMC13387609; doi:10.1371/journal.pgen.1012173)
Supplement: S1 Table — (DOCX) [file pgen.1012173.s007.docx]

## **S1 Table. Geographic locations of whole genome pools across *Antirrhinum* hybrid zone**

Details of deme locations at *Antirrhinum majus subspecies majus* hybrid zone at Planoles. For the first deme, position zero set from the first deme from the larger KASP SNP genotyping in relation to whole genome pools (poolSeq) on the yellow side with *A. m. m var striatum* (YP4, YP1, YP2) and magenta side *A. m. m var pseudomajus* (MP2, MP4, MP11). Distance along the transect (Fig 1), deme span (*d_i_*), number of samples included in poolSeq, and mean depth and standard deviation (in parentheses).

| **deme/pool** | **Easting** | **Northing** | **distance along transect (km)** | **deme span** | **number of samples** | **mean depth (SD)** |
| --- | --- | --- | --- | --- | --- | --- |
| KASP Start | 411310.104 | 4690519.39 | 0 | - | - |  |
| YP4 | 411660.104 | 4690319.39 | 0.38 | 6000 | 50 | 53.3 (21.7) |
| YP1 | 421960.104 | 4686519.39 | 11.23 | 5000 | 50 | 24.2 (6.8) |
| YP2 | 422110.104 | 4686369.39 | 11.40 | 1500 | 50 | 22.6 (6.3) |
| MP2 | 424460.104 | 4686069.39 | 13.77 | 1400 | 50 | 22.5 (6.2) |
| MP4 | 425110.104 | 4685969.39 | 14.42 | 3500 | 50 | 25.8 (7.2) |
| MP11 | 431660.104 | 4686869.39 | 20.67 | 6000 | 50 | 39.3 (15.4) |
|  |  |  |  |  |  |  |
