# Supplementary material for: Genome-wide cline analysis identifies new locus contributing to a barrier to gene flow across an Antirrhinum hybrid zone
Source: PLoS Genet. 2026 Jul 13;22(7):e1012173. doi: 10.1371/journal.pgen.1012173 (PMC13387609; doi:10.1371/journal.pgen.1012173)
Supplement: S2 Table — (DOCX) [file pgen.1012173.s008.docx]

## **S2 Table. KASP SNP genotype marker details**

Details of the KASP marker design including locus marker name (with LGC), colour loci, the colour pigment locus is associated with, the chromosome (Chr), position on chromosome and the sequence for the oligo design in IUCN code. The biallelic SNP is indicated (e.g. [A/C]).

| **Locus**  **marker name** | **colour loci** | **colour pigment** | **Chr** | **position** | **Oligo** |
| --- | --- | --- | --- | --- | --- |
| s1187_290152 | *CRE* | yellow | 1 | 955185 | GTCTCACATTGTTGACAAAATCCAAGTCGTGACTTGGGAGGAAGAATAAT[A/C]AAAATGGTCATGRTMRAGTCACTTTYCCCTTAATCACCAAAATAGAAAAA |
| s316_93292 | *FLA 1* | yellow | 2 | 53594773 | AGATTTGCTATMTGGTATTGGAGTCGCTGGAAGATAAAGAAAGTACGCCA[A/G]TCGAGCTAAACCTCCAGCTTCCTGGTTATGATGGAAACAAAGAACATGAA |
| s316_257789 | *FLA 2* | yellow | 2 |  | TGTATGTGGCAGCTTCACATTATACACAATTGCATGCAGACGAACCAATA[A/G]CCAGGGGCGTATCYARGAATTTTATCTGGGGAGGGCTAAYCTAATGAATA |
| s91_39699 | *SULF* | yellow | 4 | 38316531 | GGTAATCAAGCACATAAAAYATTAATAACAAGATTAYGAAATYARATCAA[C/T]TGGTCCACACAYATAAATCATAACACAACAATCATAACATAACCAGATTC |
| s261_720757 | *RUB* | magenta | 5 | 6286923 | CAAAGTAYGMCATTTGCACCYATTCATTTGAGAGCTCAACGATCGAATAT[C/T]GATCATAACCTCGATTTGGATCGTGCTCTTTCCCAGTTCCRTCTTGTCTC |
| ros_assembly_543443 | *ROS 1* | magenta | 6 |  | GAAACTMAAAAATTMAAGATAAATTTGCTCGTGTCAATARTAGTGAAAACTAT[A/G]CWTCAATTAGTTATTGAAAATGWAACCTGTCTATTTCTATAAGTGTTTAGCG |
| ros_assembly_715015 | *EL* | magenta | 6 | 53061094 | AGAYGTGAATTCCAATGGYTCTTCACTTCATCATCCRTTCGSCCCGGYAA[A/G]CGTCCCGCAATTAGAGACCACCTAACAGTMAAGAAAGTGAATTAATAYAA |
