# Supplementary material for: Genome-wide cline analysis identifies new locus contributing to a barrier to gene flow across an Antirrhinum hybrid zone
Source: PLoS Genet. 2026 Jul 13;22(7):e1012173. doi: 10.1371/journal.pgen.1012173 (PMC13387609; doi:10.1371/journal.pgen.1012173)
Supplement: S3 Table — (DOCX) [file pgen.1012173.s009.docx]

## **S3 Table. MLE cline parameters for best fitting model of KASP SNP genotypes**

The best fitting cline parameters for polymorphic sigmoid cline fits with simulated annealing. Estimates at each of the colour linked loci (Locus marker name) associated with each Colour loci, the chromosome (Chr), followed by the four main parameters – cline centre, cline width, allele frequencies on the left (west) of transect for *A. m. m. var. striatum* (p0) and the right (east) of transect for *A. m. m. var. pseudomajus* and their 95% confidence intervals (in parentheses).

| **Locus**  **marker name** | **Colour loci** | **Chr** | **centre** | **width** | **p0** | **p1** |
| --- | --- | --- | --- | --- | --- | --- |
| s1187_290152 | *CRE* | 1 | 13.9 (13.7 - 14) | 3.1 (2.7 – 3.4) | 0.05 (0.04 - 0.06) | 0.91 (0.90 - 0.92) |
| s316_93292 | *FLA* 1 | 2 | 13.9 (13.8 – 14.2) | 3.0 (2.6 – 3.6) | 0.03 (0.01 - 0.04) | 0.94 (0.91 - 0.97) |
| s316_257789 | *FLA* 2 | 2 | 10.5 (10.2 – 10.9) | 9.1 (8.5 – 10.1) | 0.11 (0.0 - 0.18) | 0.99 (0.99 - 0.99) |
| s91_39699 | *SULF* | 4 | 13.5 (13.5 – 13.6) | 934 (0.9 – 12.5) | 0.32 (0.32 - 0.32) | 0.93 (0.93 - 0.94) |
| s261_720757 | *RUB* | 5 | 11.9 (11.5 – 12.4) | 7.6 (6.0 – 9.1) | 0.13 (0.06 - 0.23) | 0.95 (0.90 - 0.97) |
| ros_assembly 543443 | *ROS1* | 6 | 13.2 (13.1 – 13.3) | 876 (0.8 – 10.2) | 0.11 (0.09 - 0.12) | 0.99 (0.98 - 0.99) |
