# Supplementary material for: Genome-wide cline analysis identifies new locus contributing to a barrier to gene flow across an Antirrhinum hybrid zone
Source: PLoS Genet. 2026 Jul 13;22(7):e1012173. doi: 10.1371/journal.pgen.1012173 (PMC13387609; doi:10.1371/journal.pgen.1012173)

## **S4 Table. Test for greater clustering of divergent loci showing clines on each LG (Chromosome) than expected by chance.**

LG: linkage group (chromosome). Obs. mean pairwise distance: the mean pairwise distance between clinal windows in Mbp. The mean pairwise distance in Mbp calculated from the 99,999 random permutations. The p-values show the probability of obtaining the observed mean pairwise distance by chance.


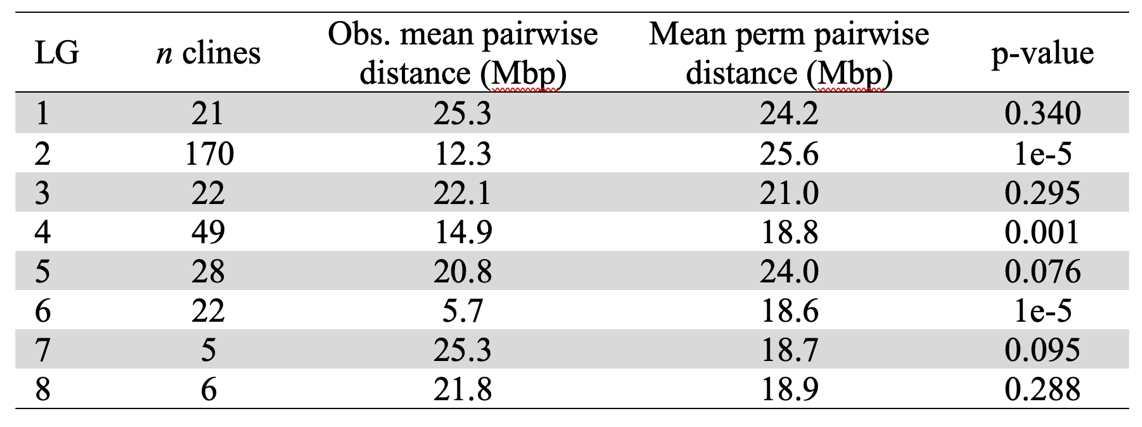

Supplement: S4 Table — (DOCX) [file pgen.1012173.s010.docx]
