# Supplementary material for: Genome-wide cline analysis identifies new locus contributing to a barrier to gene flow across an Antirrhinum hybrid zone
Source: PLoS Genet. 2026 Jul 13;22(7):e1012173. doi: 10.1371/journal.pgen.1012173 (PMC13387609; doi:10.1371/journal.pgen.1012173)

## **S5 Table. Test for enriched overlap of clinal windows and *F_ST_* outliers.**

Pops: the populations that *F_ST_* was calculated between. ∆P: the allele frequency cut-off used in the *fastclines* analysis. n clines: the number of clines detected in the *fastcline*s analysis. Observed overlaps: the number of clinal windows that were also *F_ST_* outliers. Mean perm overlaps: the mean number of overlaps, calculated from the 99,999 random permutations. The p-values show the probability of obtaining the observed number of overlaps by chance


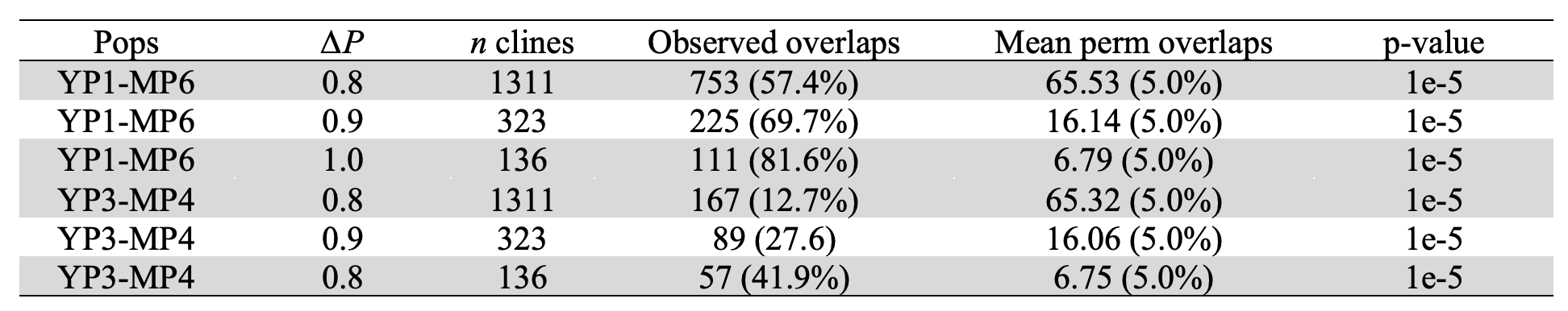

Supplement: S5 Table — (DOCX) [file pgen.1012173.s011.docx]
