# Supplementary material for: Genome-wide cline analysis identifies new locus contributing to a barrier to gene flow across an Antirrhinum hybrid zone
Source: PLoS Genet. 2026 Jul 13;22(7):e1012173. doi: 10.1371/journal.pgen.1012173 (PMC13387609; doi:10.1371/journal.pgen.1012173)

## **S6 Table. Test for differences in *π, d_xy_* and *F_ST_* between clinal and non-clinal windows between population pairs.**

DP: depth cut-off for including a site in the dataset. Pop1 and Pop2: IDs of the populations being compared. ∆P: the allele frequency cut-off used in the *fastclines* analysis*.* The absolute value of the observed difference between the mean clinal and non-clinal values loci are given. The p-values show the probability of obtaining the observed difference by chance, determined using a permutation test (99,999 random permutations).


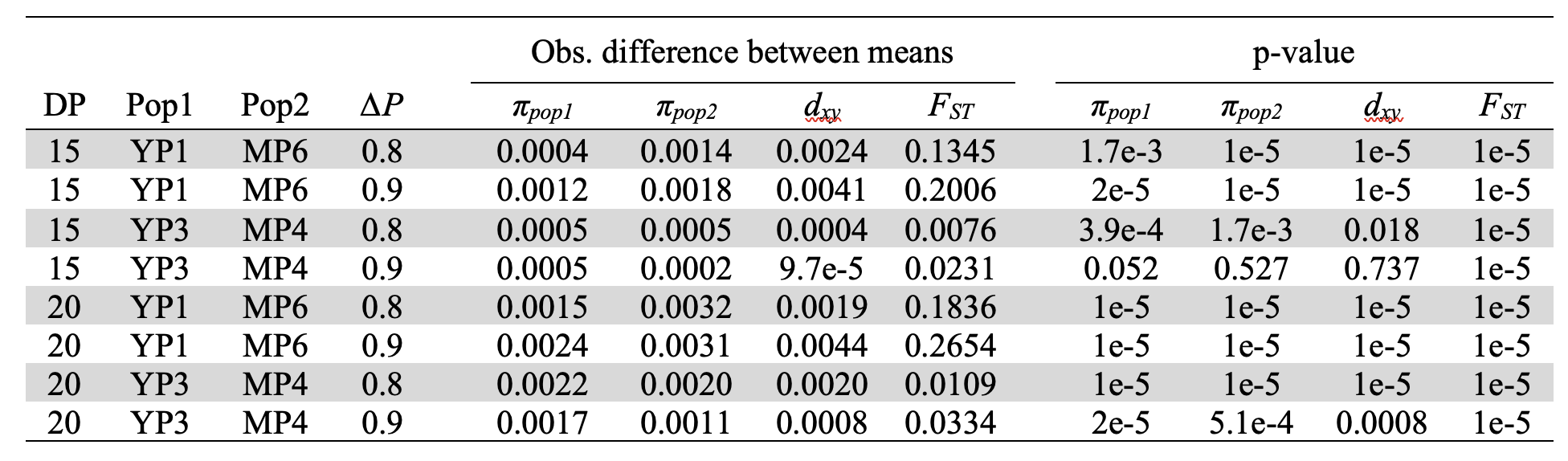

Supplement: S6 Table — (DOCX) [file pgen.1012173.s012.docx]
