# Supplementary material for: Genome-wide cline analysis identifies new locus contributing to a barrier to gene flow across an Antirrhinum hybrid zone
Source: PLoS Genet. 2026 Jul 13;22(7):e1012173. doi: 10.1371/journal.pgen.1012173 (PMC13387609; doi:10.1371/journal.pgen.1012173)
Supplement: S7 Table — (DOCX) [file pgen.1012173.s013.docx]

## **S7 Table. Linear regressions of genotype variation at *ROS* and *RUB* loci and quantitative flower colour scores for Hue in HSV colour space.**

Overall model (intercept), individual loci (RUB, ROS1) and their interaction (RUB:ROS1) effects, standard error, p-value (p < 0.05 in bold) and Model (the six floral regions in Fig S15)

| **term** | **estimate** | **std.error** | **statistic** | **p.value** | **Model** |
| --- | --- | --- | --- | --- | --- |
| (Intercept) | 47.95976 | 14.39679 | 3.331281 | **0.000933** | Hue 1 |
| RUB | 29.91842 | 10.79177 | 2.772336 | **0.005787** | Hue 1 |
| ROS1 | 153.1438 | 12.55363 | 12.19916 | **6.39E-30** | Hue 1 |
| RUB:ROS1 | -15.8073 | 8.584783 | -1.84132 | 0.066206 | Hue 1 |
| (Intercept) | 44.86354 | 11.70712 | 3.832158 | **0.000144** | Hue 2 |
| RUB | -4.38909 | 8.775609 | -0.50015 | 0.617207 | Hue 2 |
| ROS1 | 5.966213 | 10.20831 | 0.584447 | 0.559201 | Hue 2 |
| RUB:ROS1 | 13.37783 | 6.980937 | 1.916337 | 0.05593 | Hue 2 |
| (Intercept) | 45.07245 | 15.9481 | 2.826197 | **0.004912** | Hue 3 |
| RUB | -5.62737 | 11.95462 | -0.47073 | 0.638054 | Hue 3 |
| ROS1 | 44.90034 | 13.90633 | 3.22877 | **0.00133** | Hue 3 |
| RUB:ROS1 | 28.94835 | 9.509822 | 3.044047 | **0.002465** | Hue 3 |
| (Intercept) | 37.36295 | 14.20502 | 2.630264 | **0.008812** | Hue 4 |
| RUB | -5.66573 | 10.64802 | -0.53209 | 0.594914 | Hue 4 |
| ROS1 | 75.99549 | 12.38641 | 6.135391 | **1.81E-09** | Hue 4 |
| RUB:ROS1 | 30.53757 | 8.470431 | 3.605197 | **0.000345** | Hue 4 |
| (Intercept) | 23.83616 | 11.92909 | 1.998154 | **0.046277** | Hue 5 |
| RUB | 2.262911 | 8.941996 | 0.253066 | 0.800328 | Hue 5 |
| ROS1 | 133.4256 | 10.40186 | 12.82709 | **1.7E-32** | Hue 5 |
| RUB:ROS1 | 7.261483 | 7.113296 | 1.020832 | 0.30786 | Hue 5 |
| (Intercept) | 23.73608 | 12.04642 | 1.970386 | **0.049382** | Hue 6 |
| RUB | 3.214847 | 9.029942 | 0.356021 | 0.721985 | Hue 6 |
| ROS1 | 132.4046 | 10.50416 | 12.60496 | **1.41E-31** | Hue 6 |
| RUB:ROS1 | 7.46519 | 7.183257 | 1.039249 | 0.299225 | Hue 6 |
