# Supplementary figures and images for: Genome-wide cline analysis identifies new locus contributing to a barrier to gene flow across an Antirrhinum hybrid zone
Source: PLoS Genet. 2026 Jul 13;22(7):e1012173. doi: 10.1371/journal.pgen.1012173 (PMC13387609; doi:10.1371/journal.pgen.1012173)

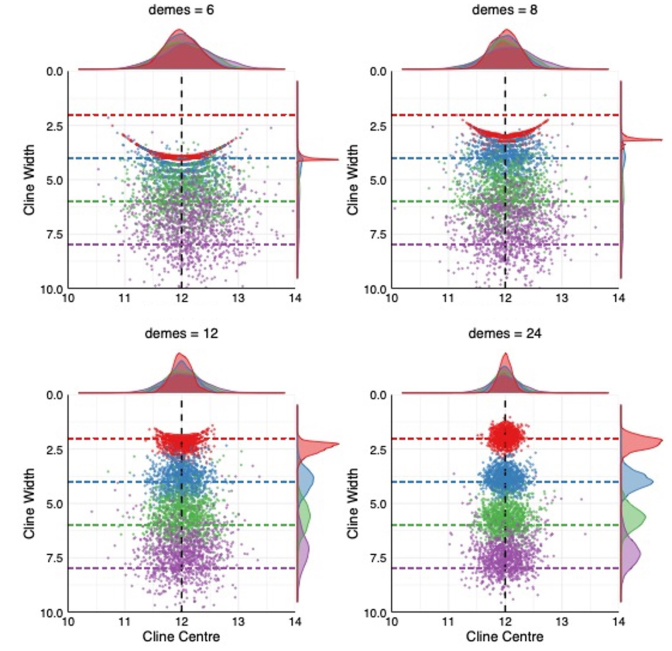

Supplement: S1 Fig — (TIFF) [file pgen.1012173.s014.tiff]

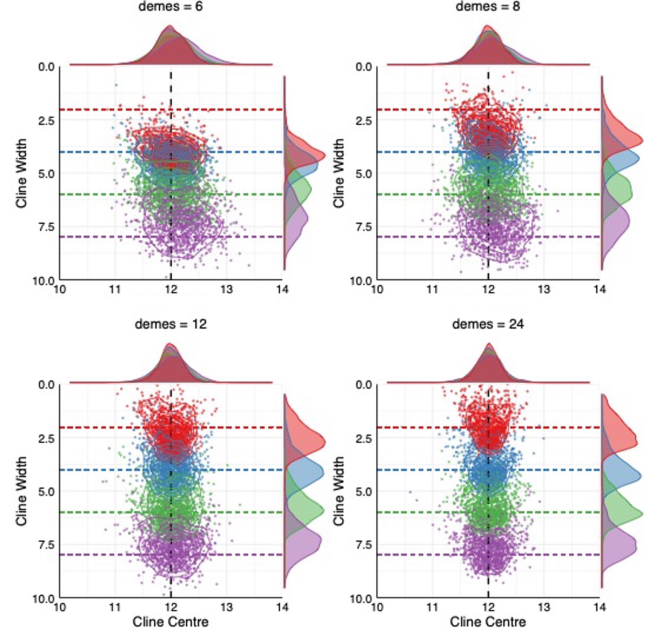

Supplement: S2 Fig — (TIFF) [file pgen.1012173.s015.tiff]

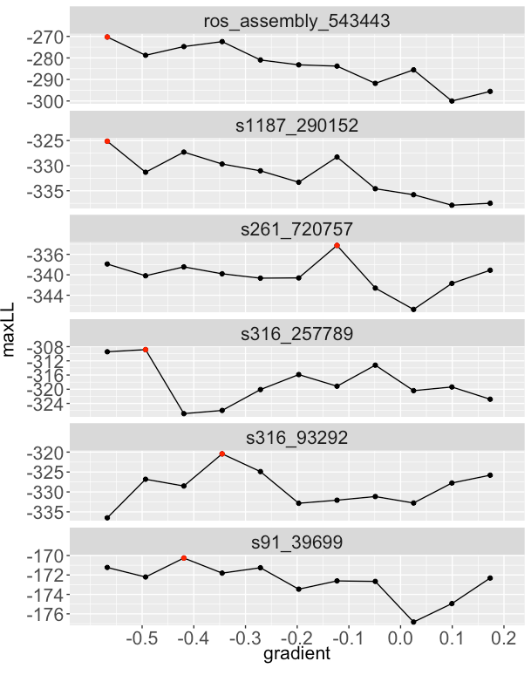

Supplement: S3 Fig — (TIFF) [file pgen.1012173.s016.tiff]

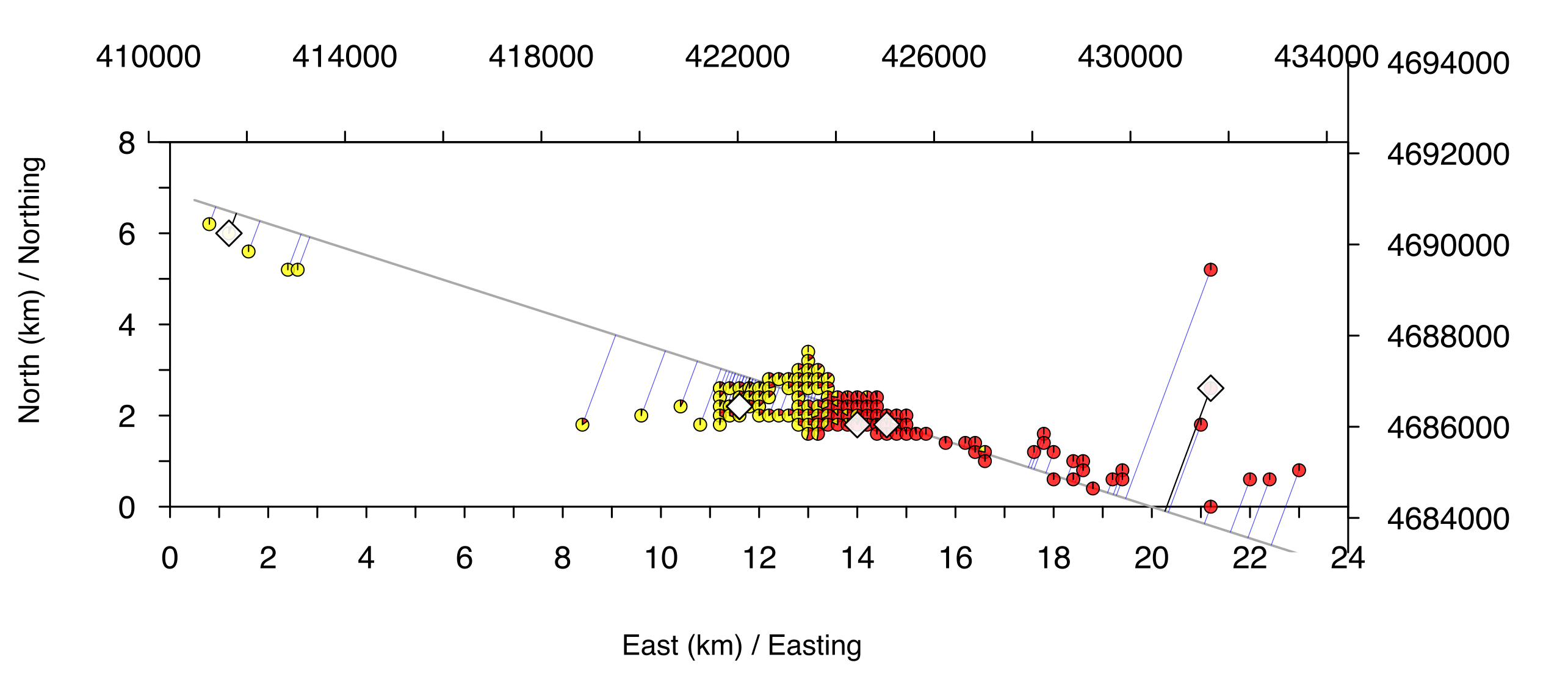

Supplement: S4 Fig — (TIFF) [file pgen.1012173.s017.tiff]

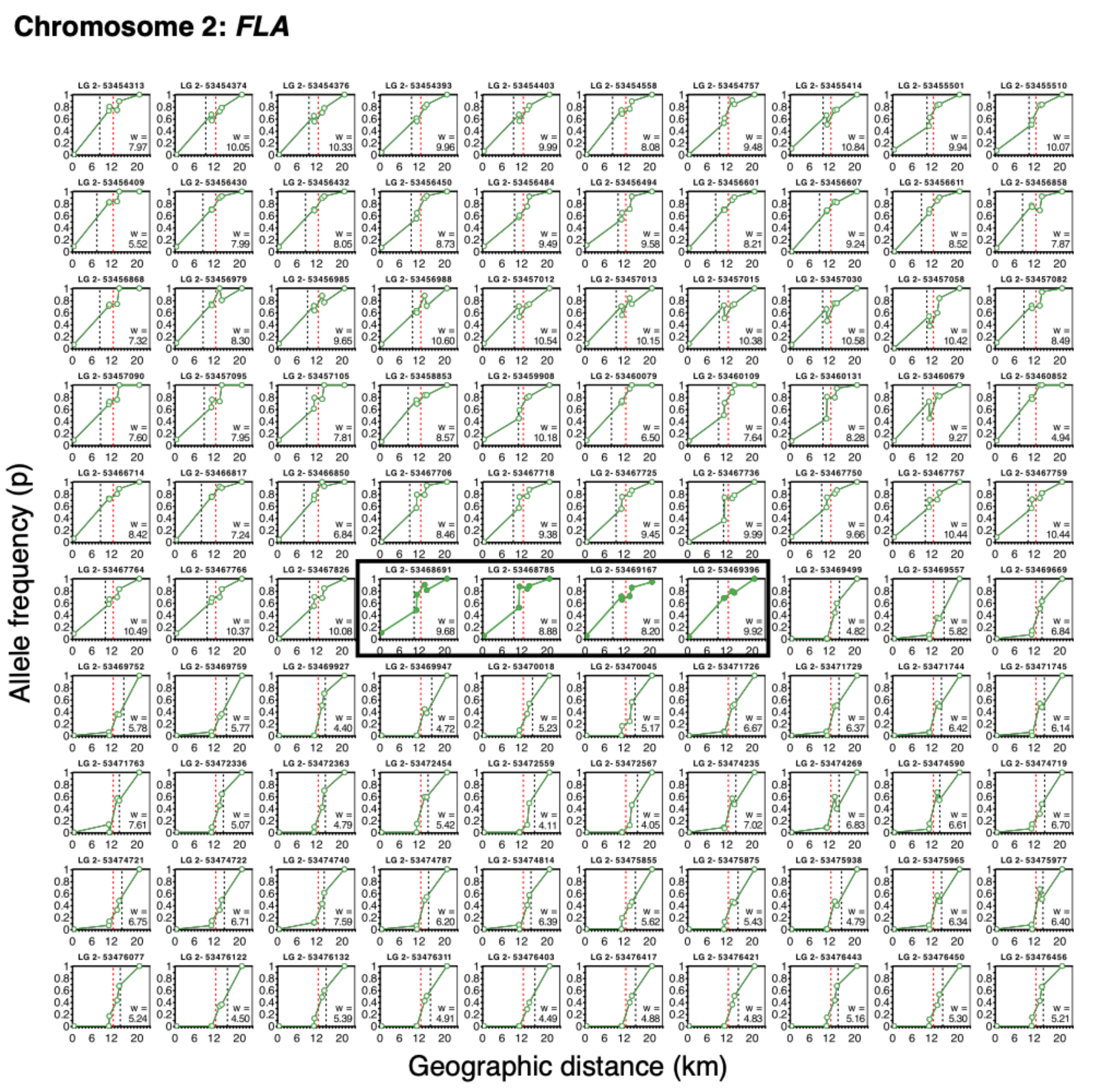

Supplement: S5 Fig — (TIFF) [file pgen.1012173.s018.tiff]

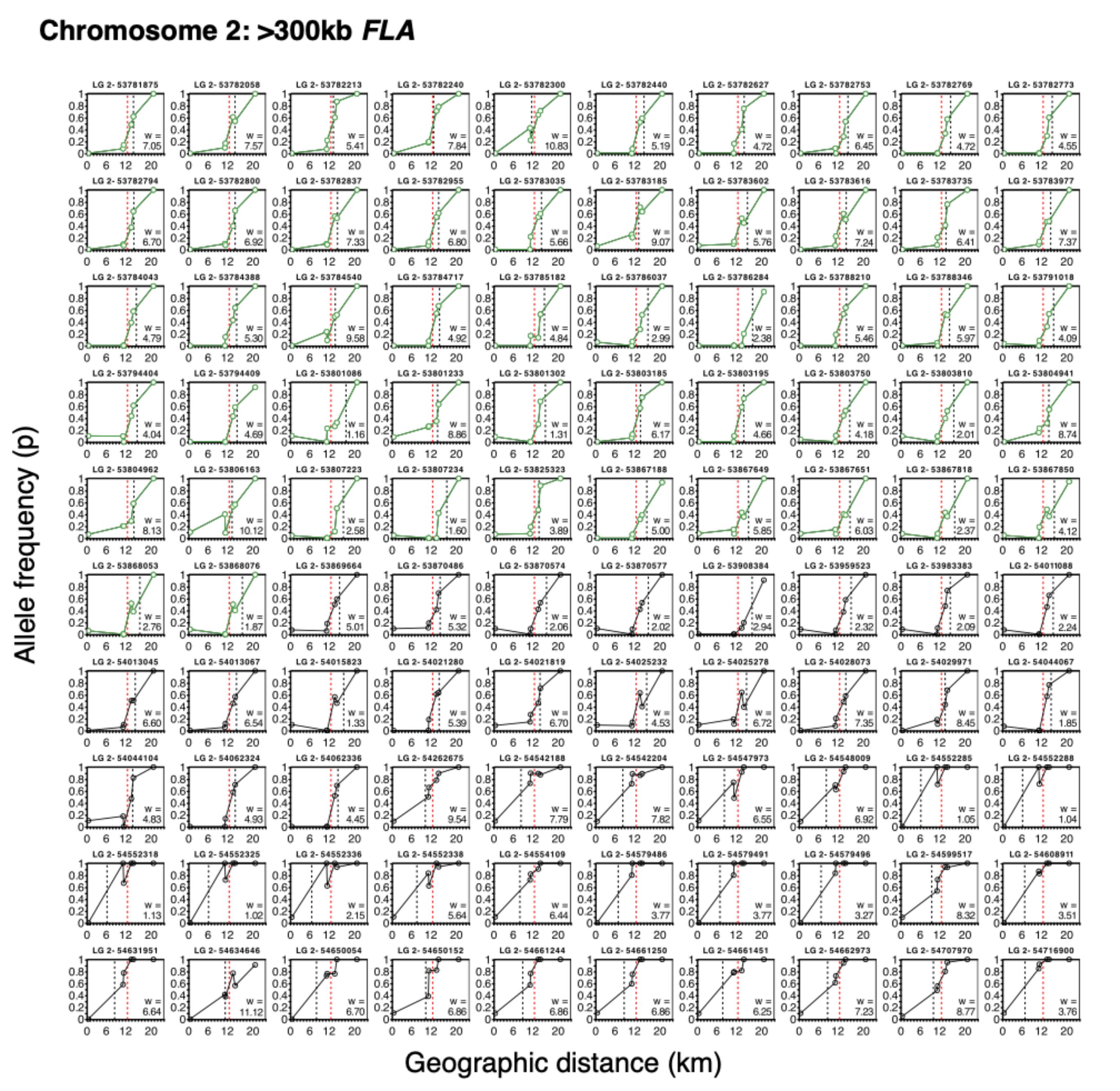

Supplement: S6 Fig — (TIFF) [file pgen.1012173.s019.tiff]

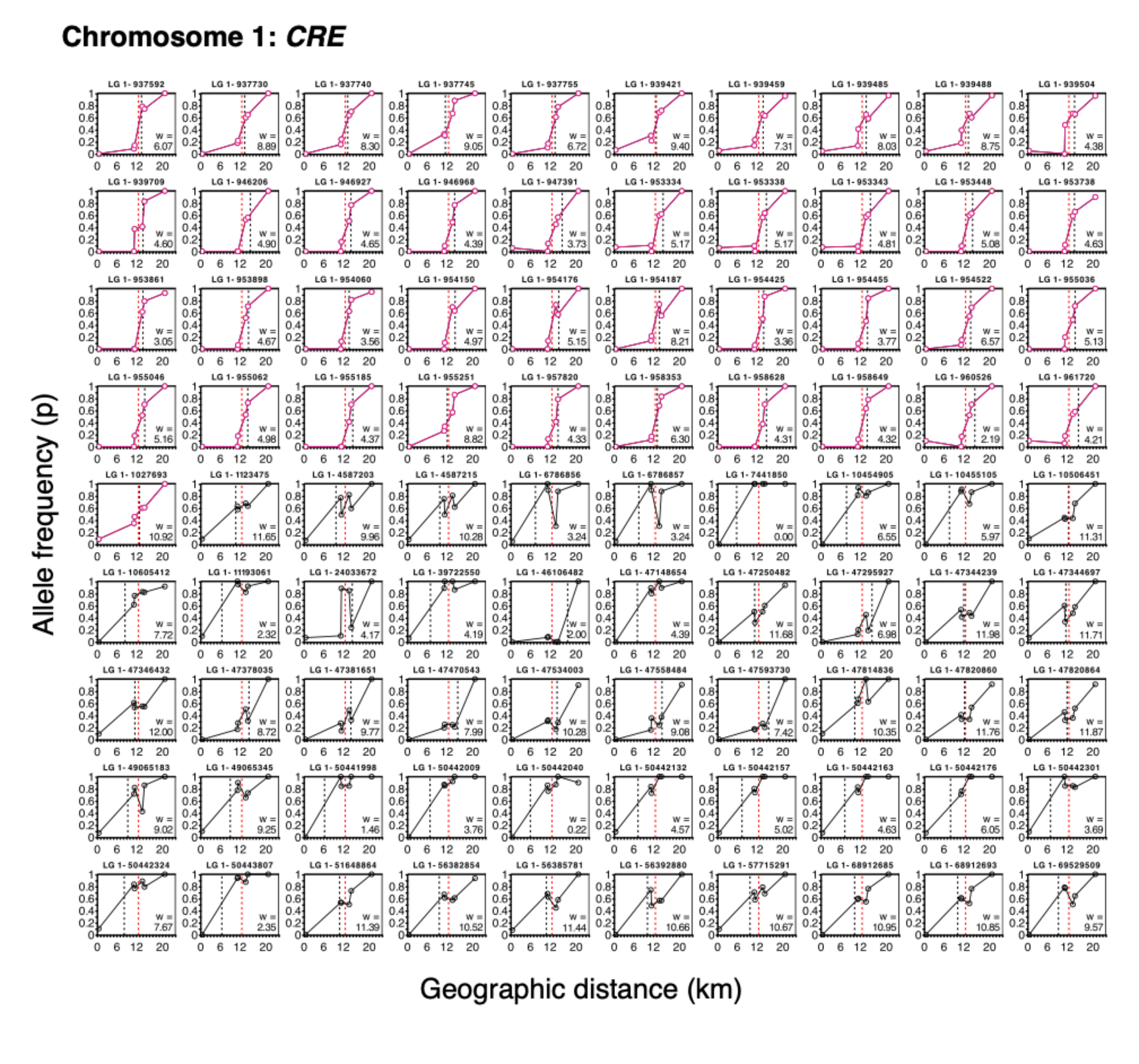

Supplement: S7 Fig — (TIFF) [file pgen.1012173.s020.tiff]

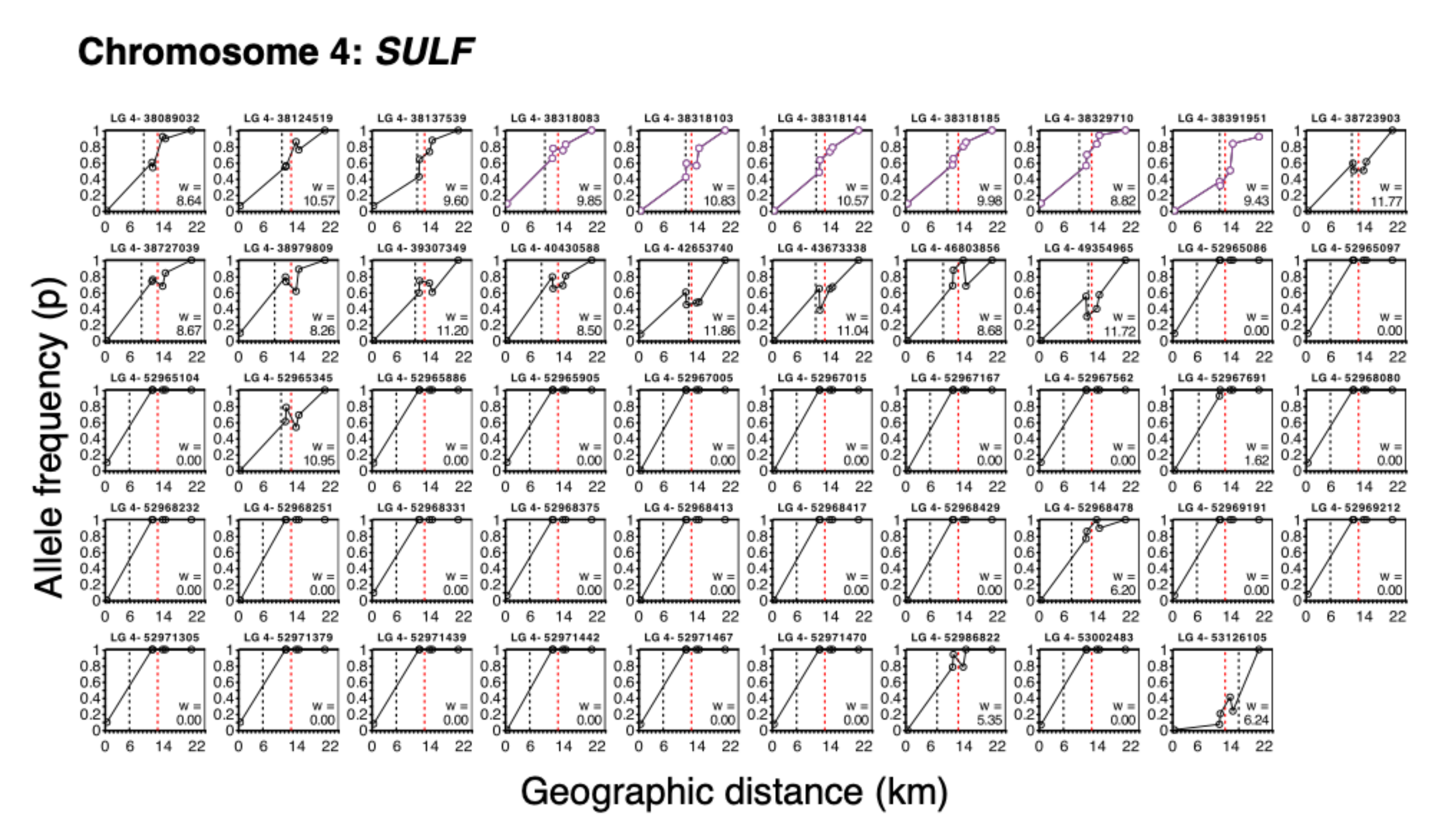

Supplement: S8 Fig — (TIFF) [file pgen.1012173.s021.tiff]

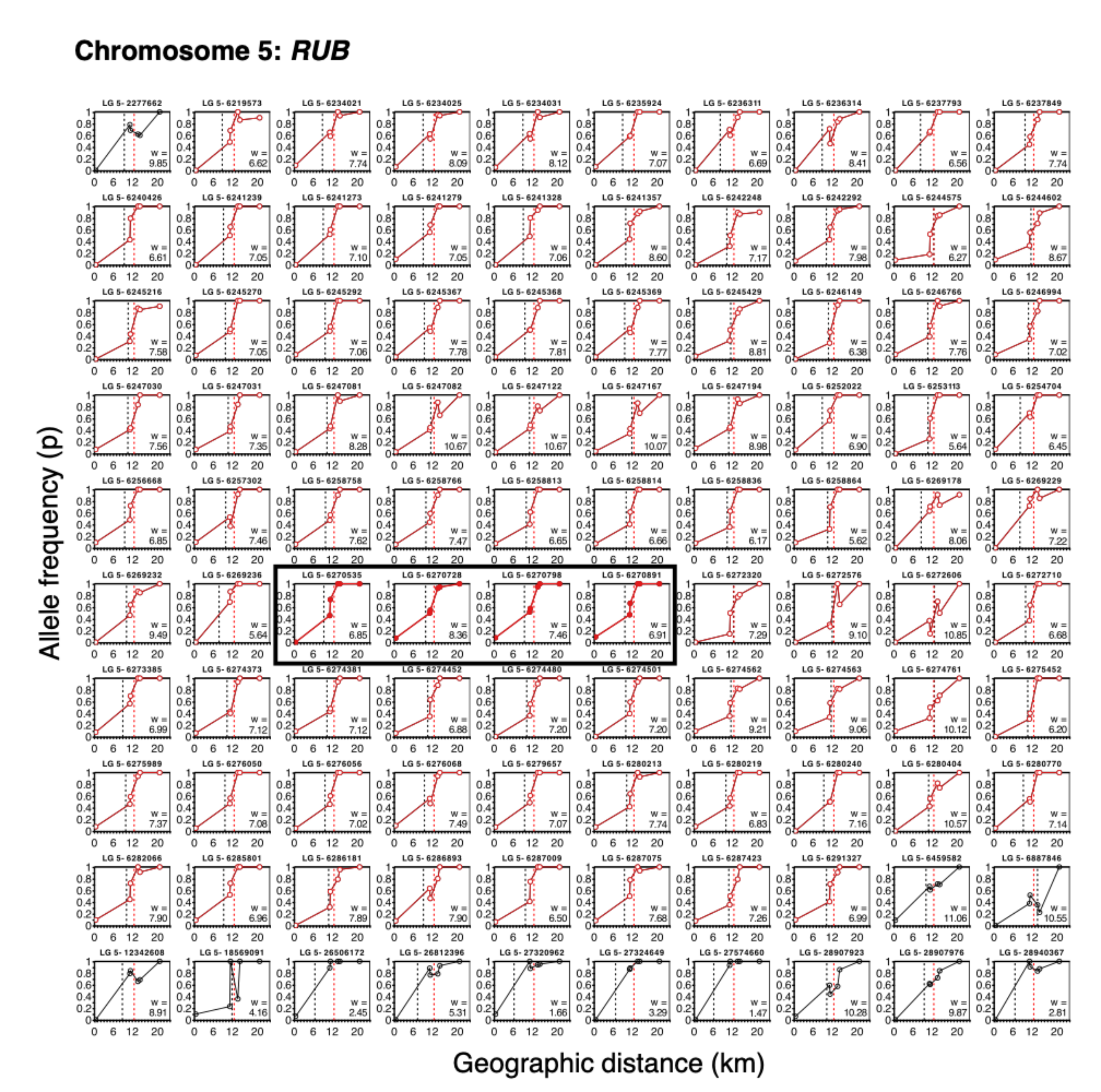

Supplement: S9 Fig — (TIFF) [file pgen.1012173.s022.tiff]

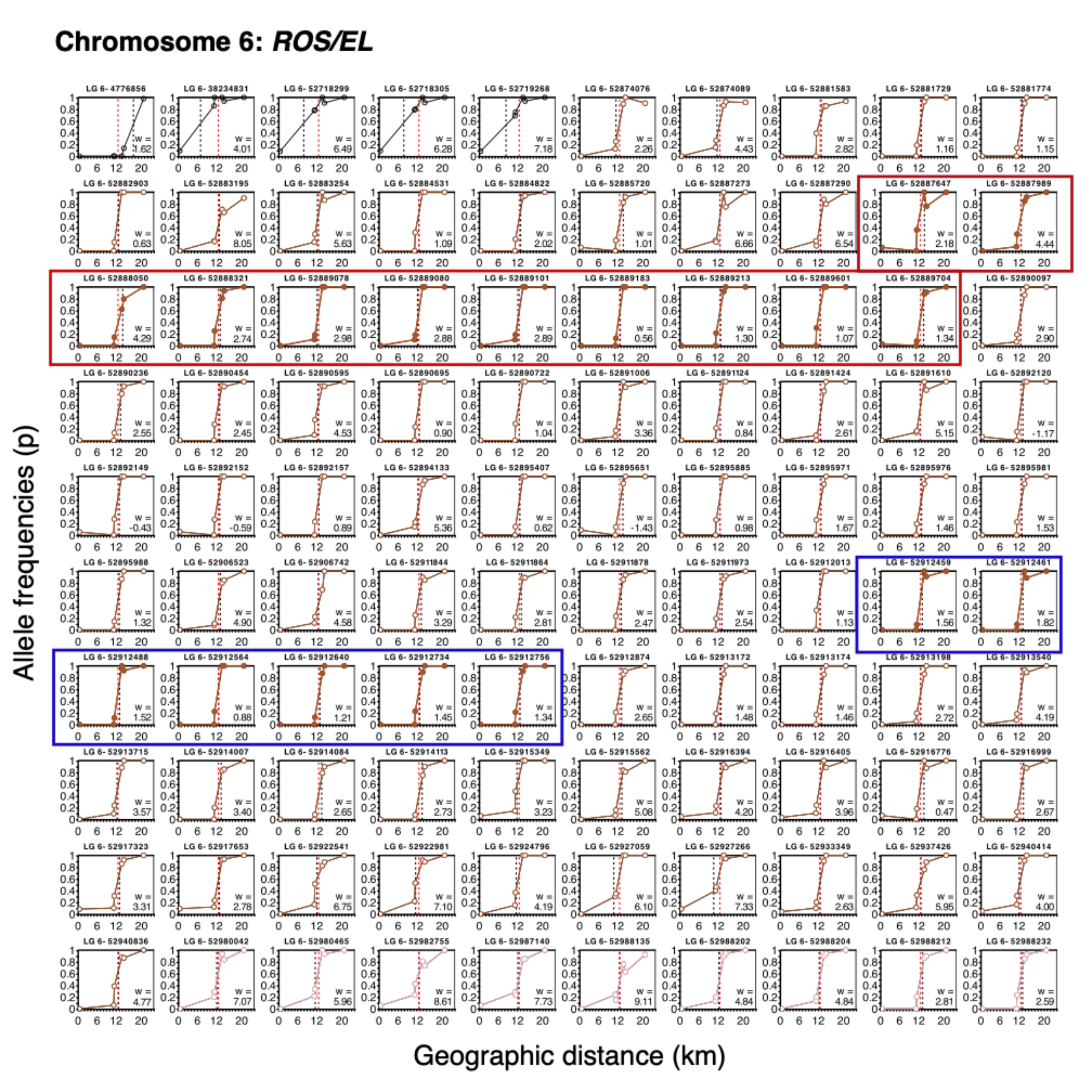

Supplement: S10 Fig — (TIFF) [file pgen.1012173.s023.tiff]

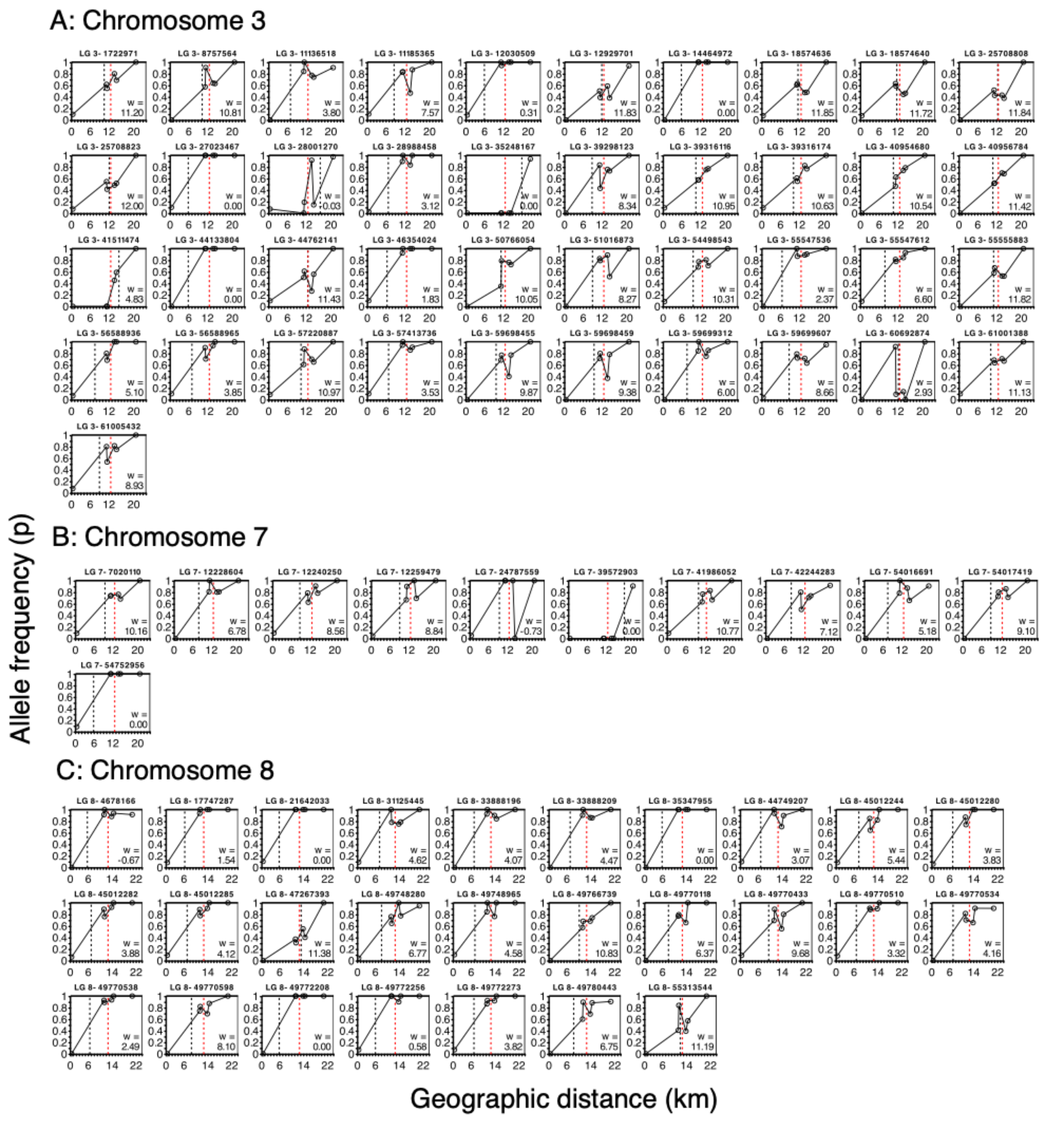

Supplement: S11 Fig — (TIFF) [file pgen.1012173.s024.tiff]

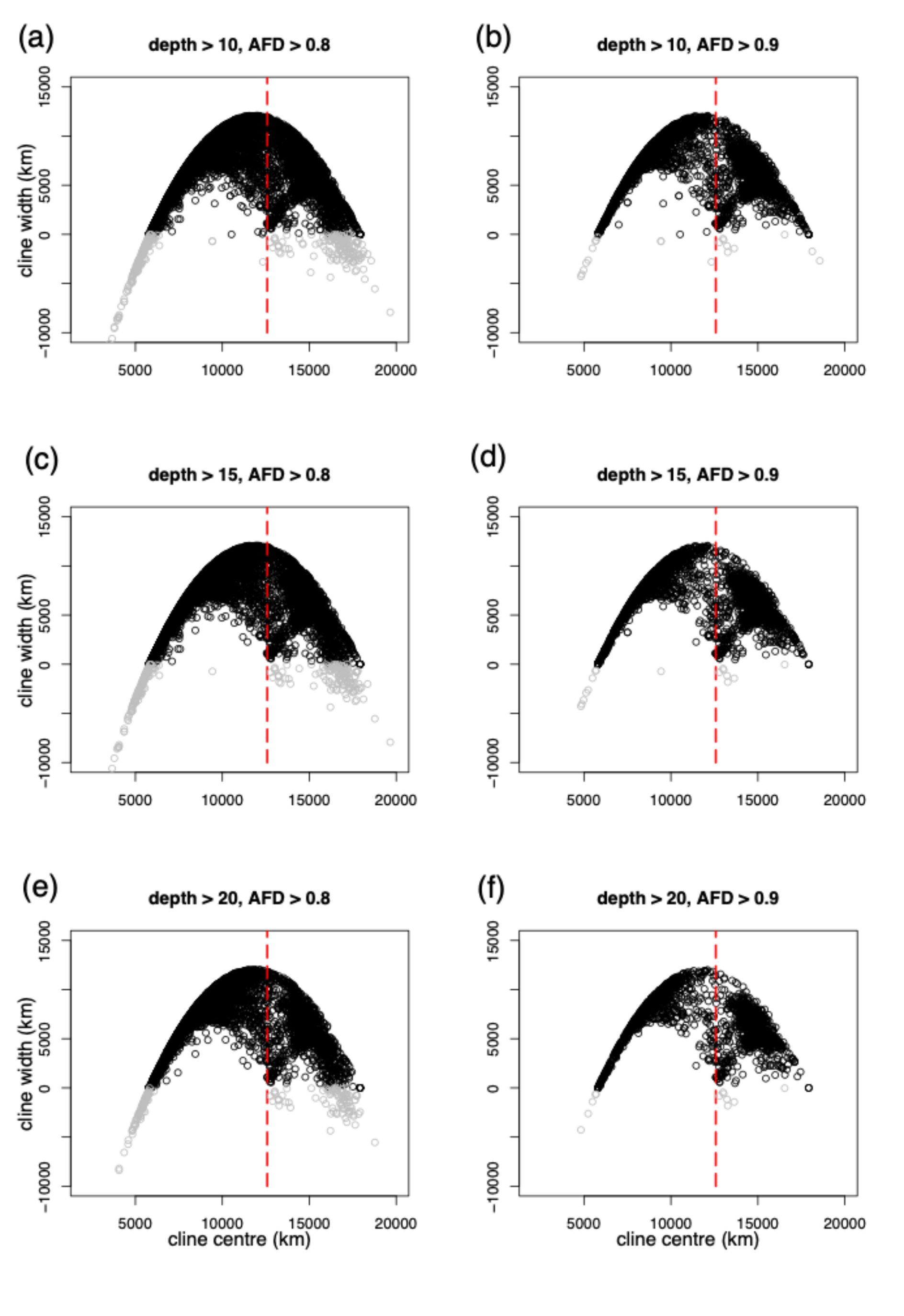

Supplement: S12 Fig — (TIFF) [file pgen.1012173.s025.tiff]

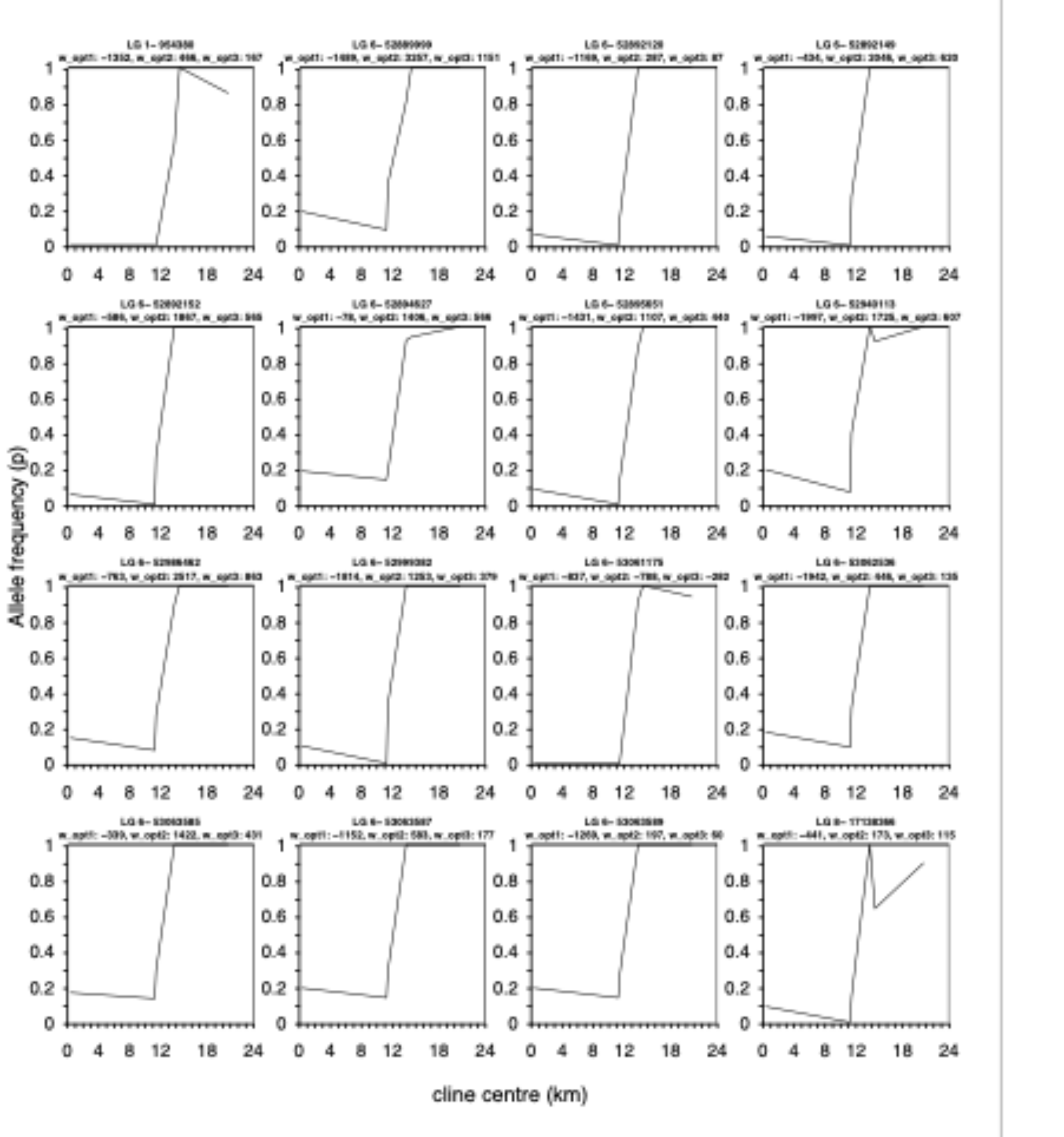

Supplement: S13 Fig — (TIFF) [file pgen.1012173.s026.tiff]

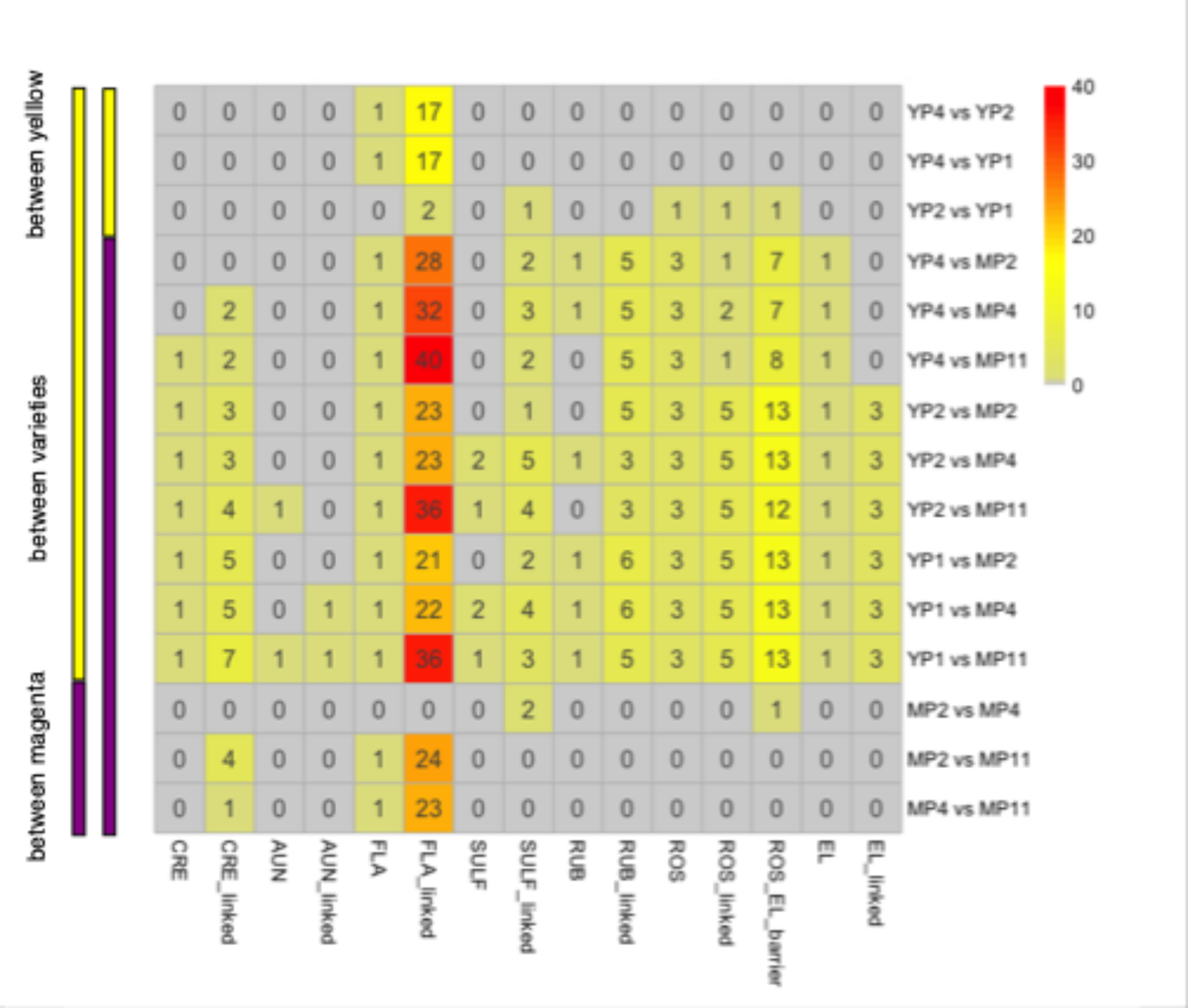

Supplement: S14 Fig — (TIFF) [file pgen.1012173.s027.tiff]

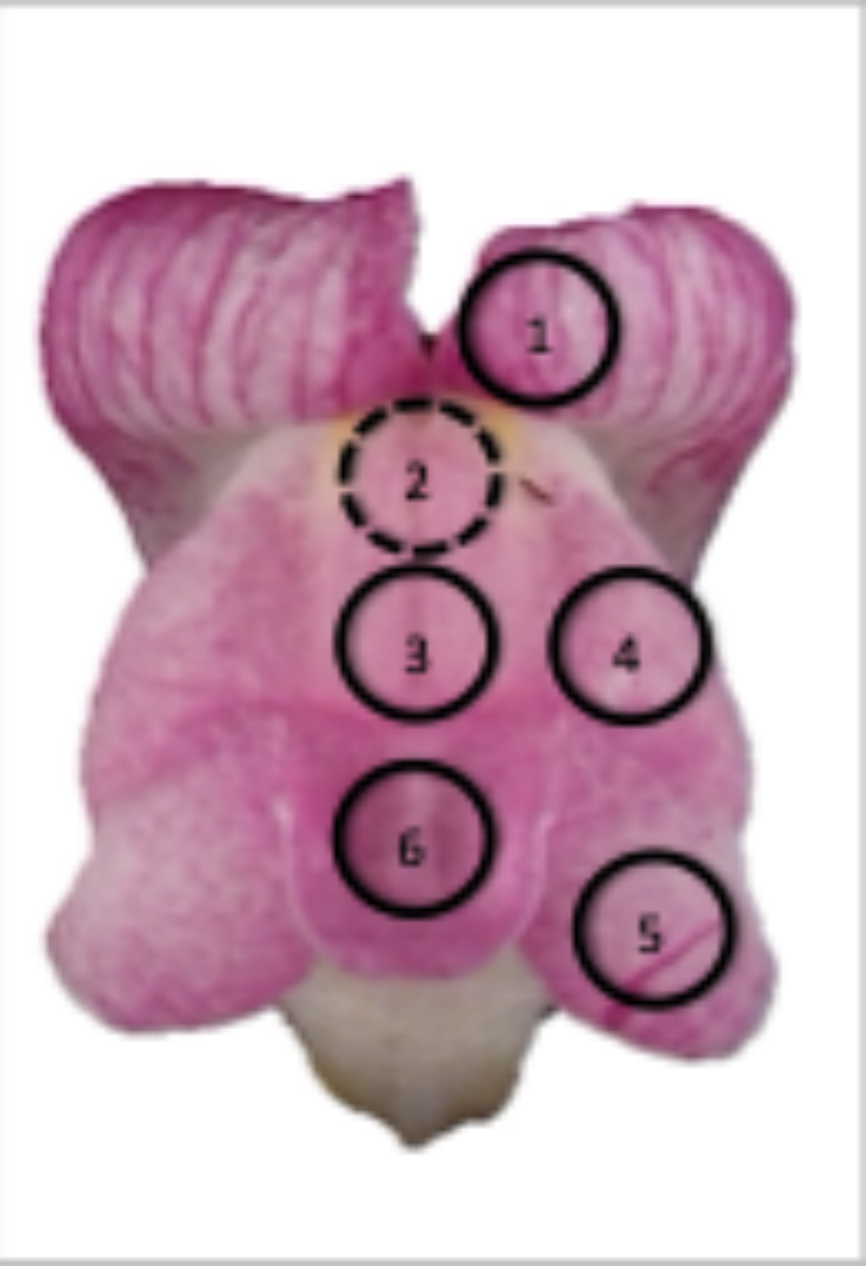

Supplement: S15 Fig — (TIFF) [file pgen.1012173.s028.tiff]

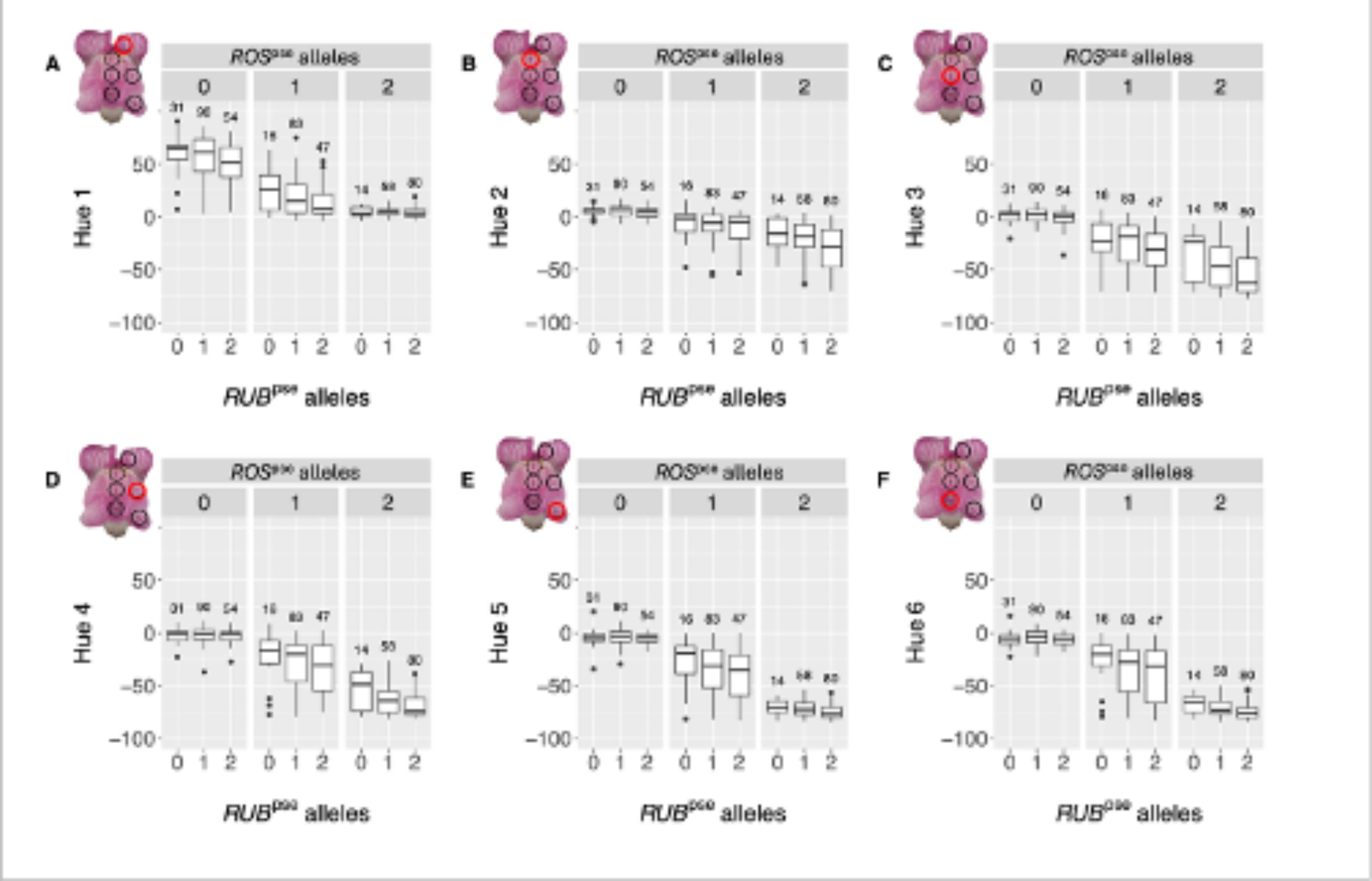

Supplement: S16 Fig — (TIFF) [file pgen.1012173.s029.tiff]
