# Supplementary material for: Genome-wide cline analysis identifies new locus contributing to a barrier to gene flow across an Antirrhinum hybrid zone
Source: PLoS Genet. 2026 Jul 13;22(7):e1012173. doi: 10.1371/journal.pgen.1012173 (PMC13387609; doi:10.1371/journal.pgen.1012173)
Supplement: S17 Fig — (DOCX) [file pgen.1012173.s030.docx]

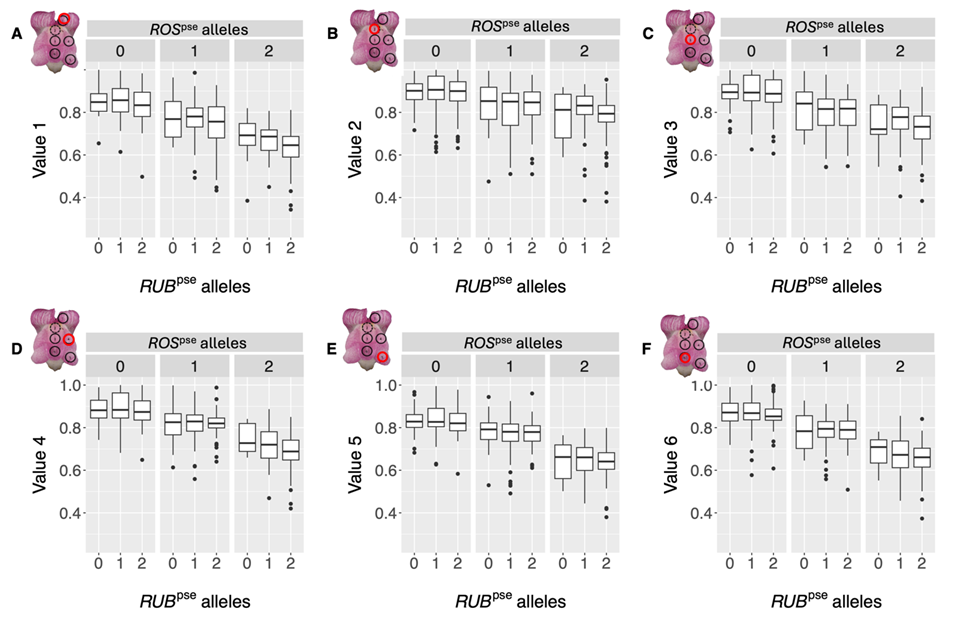


**S17 Fig. Summary of HSV Saturation scores of Antirrhinum flowers for ROS and RUB haplotypes from the hybrid zone.**

**Saturation (in HSV colour space) in six regions of the flower (a – f) for 473 plants from the hybrid zone. For each panel, plants are grouped haplotypes, first via facets which group plants by the number of copies of ROS alleles from A. m. m. pseudomajus (ROSpse) and secondly by the number of RUB alleles from A. m. m. pseudomajus (RUBpse) along x-axis. The numbers above each box indicate sample size. Insets of flower images indicate the focus region of the Saturation measurements with a red circle.**
