# Supplementary material for: Genome-wide cline analysis identifies new locus contributing to a barrier to gene flow across an Antirrhinum hybrid zone
Source: PLoS Genet. 2026 Jul 13;22(7):e1012173. doi: 10.1371/journal.pgen.1012173 (PMC13387609; doi:10.1371/journal.pgen.1012173)
Supplement: S18 Fig — (DOCX) [file pgen.1012173.s031.docx]

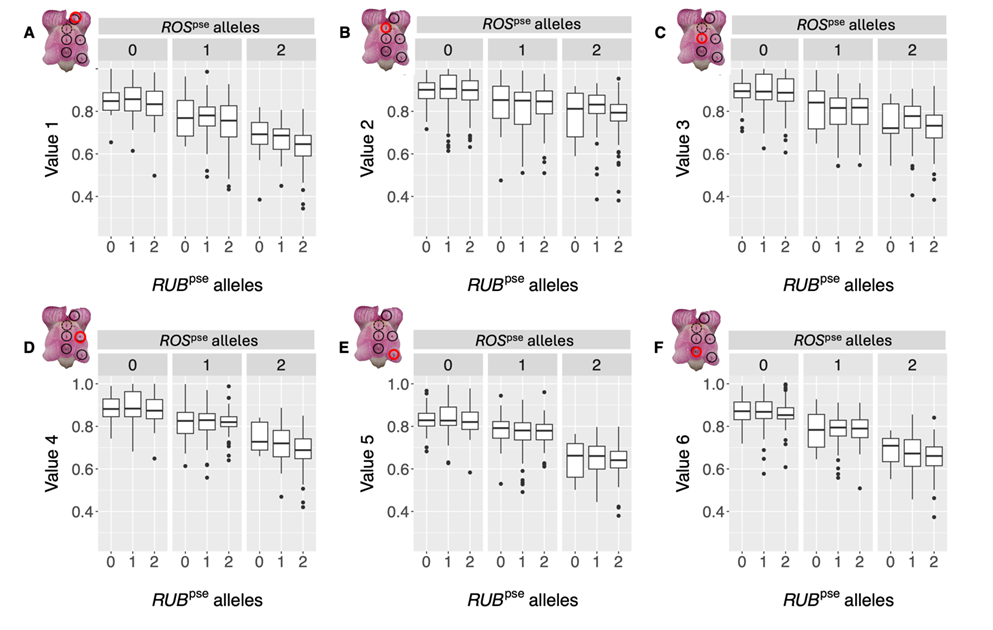


## **S18 Fig. Summary of HSV Value scores of *Antirrhinum* flowers for *ROS* and *RUB* haplotypes from the hybrid zone.**

**Saturation (in HSV colour space) in six regions of the flower (a – f) for 473 plants from the hybrid zone. For each panel, plants are grouped haplotypes, first via facets which group plants by the number of copies of *ROS* alleles from *A. m. m. pseudomajus* (*ROS^pse^*) and secondly by the number of *RUB* alleles from *A. m. m. pseudomajus* (*RUB^pse^*) along x-axis. The numbers above each box indicate sample size. Insets of flower images indicate the focus region of the Saturation measurements with a red circle**.
