# Supplementary material for: Genome-wide cline analysis identifies new locus contributing to a barrier to gene flow across an Antirrhinum hybrid zone
Source: PLoS Genet. 2026 Jul 13;22(7):e1012173. doi: 10.1371/journal.pgen.1012173 (PMC13387609; doi:10.1371/journal.pgen.1012173)
Supplement: Attachment — Submitted filename: Field_WGS_PloSGen_Response_to_reviewers.docx [file pgen.1012173.s033.docx]

PGENETICS-D-25-00935
Genome-wide cline analysis identifies new locus contributing to a barrier to gene flow across an Antirrhinum hybrid zone
PLOS Genetics

Dear Dr. Field,

Thank you for submitting your manuscript to PLOS Genetics. After careful consideration, we feel that it has merit but does not fully meet PLOS Genetics's publication criteria as it currently stands. Therefore, we invite you to submit a revised version of the manuscript that addresses the points raised during the review process.

Please submit your revised manuscript within by Jan 22 2026 11:59PM. If you will need more time than this to complete your revisions, please reply to this message or contact the journal office at [plosgenetics@plos.org](mailto:plosgenetics@plos.org). When you're ready to submit your revision, log on to [https://www.editorialmanager.com/pgenetics/](https://url.au.m.mimecastprotect.com/s/GK33C81Vq2C6k4mGGcnf8FyrBTh?domain=editorialmanager.com) and select the 'Submissions Needing Revision' folder to locate your manuscript file.

Please include the following items when submitting your revised manuscript:

* A letter that responds to each point raised by the editor and reviewer(s). You should upload this letter as a separate file labeled 'Response to Reviewers'. This file does not need to include responses to formatting updates and technical items listed in the 'Journal Requirements' section below.
* A marked-up copy of your manuscript that highlights changes made to the original version. You should upload this as a separate file labeled 'Revised Manuscript with Track Changes'.
* An unmarked version of your revised paper without tracked changes. You should upload this as a separate file labeled 'Manuscript'.

If you would like to make changes to your financial disclosure, competing interests statement, or data availability statement, please make these updates within the submission form at the time of resubmission. Guidelines for resubmitting your figure files are available below the reviewer comments at the end of this letter.

We look forward to receiving your revised manuscript.

Kind regards,

Nicolas Bierne
Academic Editor
PLOS Genetics

Justin Fay
Section Editor
PLOS Genetics

Aimée Dudley
Editor-in-Chief
PLOS Genetics

Anne Goriely
Editor-in-Chief
PLOS Genetics

**Additional Editor Comments:**

Dear Dr Field,
I am pleased to inform you that I have obtained three reviews of your article, 'Genome-wide cline analysis identifies new locus contributing to a barrier to gene flow across an Antirrhinum hybrid zone', which has been transferred from PloS Biology to PloS Genetics. Two of the reviewers are the same as in the previous review round and one is new. Despite the delay in obtaining all the reports, you will see that only a short list of minor issues needs to be addressed to ensure this manuscript is accepted. I am delighted that this study on the genomic analysis of geographic clines is to be published in PLOS Genetics. I agree with reviewers 1 and 2 that you should discuss the differences between YP4 and YP2 in more detail, as well as the effect of a barrier to dispersal at the mountain, and you should also engage in a more thorough cross-discussion with Pal et al.'s article in Molecular Ecology. It seems to me that a multilocus barrier could indeed be trapped at the barrier to dispersal at the mountain, with colour loci clines having escaped this barrier and positioned themselves between YP1 and MP2. However, having read the two articles, I get the impression that you favour the hypothesis of local introgression (genome swamping) in YP1 and YP2. Consequently, the differences between YP2 and YP4 would be solely due to geographical isolation rather than genetic barriers. All of this needs to be clarified and better discussed. This would also shift the focus of the discussion away from the method and the detection of colour loci (which have been detected regardless of the approach used) towards discussing the potential of genome-wide cline analysis to interpret genetic barriers (I mean that if a multilocus genetic barrier is caught on a mountain, we can't expect to fit clines too well, and that the approach works for rather soft barriers and in areas without too many obstacles to dispersal).
Thank you for submitting your very interesting study to PloS Genet. I look forward to reading the revised version for upcoming publication.
Best regards,
Nicolas Bierne

We appreciate these comments and for the time taken in handling this manuscript. We have addressed all of the reviewer comments, and have also reworked parts of the discussion to lay out and discuss the alternative explanations for the discontinuities in the geographic distributions in light of the findings of Pal et al. We hope that you find these changes satisfactory.


**Journal Requirements:**

1) Please provide an Author Summary. This should appear in your manuscript between the Abstract (if applicable) and the Introduction, and should be 150-200 words long. The aim should be to make your findings accessible to a wide audience that includes both scientists and non-scientists. Sample summaries can be found on our website under Submission Guidelines:
[https://journals.plos.org/plosgenetics/s/submission-guidelines#loc-parts-of-a-submission](https://url.au.m.mimecastprotect.com/s/eUuCC91W8rCkO7J00hEhjFqO3Xz?domain=journals.plos.org)

Author summary now included

2) Some material included in your submission may be copyrighted. According to PLOSu2019s copyright policy, authors who use figures or other material (e.g., graphics, clipart, maps) from another author or copyright holder must demonstrate or obtain permission to publish this material under the Creative Commons Attribution 4.0 International (CC BY 4.0) License used by PLOS journals. Please closely review the details of PLOSu2019s copyright requirements here: PLOS Licenses and Copyright. If you need to request permissions from a copyright holder, you may use PLOS's Copyright Content Permission form.
Please respond directly to this email and provide any known details concerning your material's license terms and permissions required for reuse, even if you have not yet obtained copyright permissions or are unsure of your material's copyright compatibility. Once you have responded and addressed all other outstanding technical requirements, you may resubmit your manuscript within Editorial Manager. 
Potential Copyright Issues:

- Please confirm (a) that you are the photographer of Figures 7, 8, and Supporting Information file., or (b) provide written permission from the photographer to publish the photo(s) under our CC BY 4.0 license.

Photographer is Desmond Bradley, who is also an author on the manuscript. He provided permission in this written statement via email:

Dear PLOS,

The flower photos in figures 7,8 and Supporting information were taken by me, from plant our lab’s own plant populations  and I am very happy to agree to them being published in your journal as part of the paper by David L. Field et al. “Genome-wide cline analysis identifies new locus contributing to a barrier to gene flow across an Antirrhinum hybrid zone”

With best wishes

Desmond Bradley

3) Please amend your detailed Financial Disclosure statement. This is published with the article. It must therefore be completed in full sentences and contain the exact wording you wish to be published.
- State the initials, alongside each funding source, of each author to receive each grant. For example: "This work was supported by the National Institutes of Health (####### to AM; ###### to CJ) and the National Science Foundation (###### to AM)."
- State what role the funders took in the study. If the funders had no role in your study, please state: "The funders had no role in study design, data collection and analysis, decision to publish, or preparation of the manuscript.".
If you did not receive any funding for this study, please simply state: u201cThe authors received no specific funding for this work.u201d

Funding details included in revised version

**Reviewers' comments:**

Reviewer's Responses to Questions

**Comments to the Authors:
Please note here if the review is uploaded as an attachment.**

Reviewer #1: Overview

I stand by my original review of this work for PLoS Biology:

“This is an excellent study applying new methodology to, and reporting new biology of, the Antirrhinum system. The planning, layout and argumentation are lucid. The FastClines approach elegantly plugs an important computational hole in genomic studies of hybridisation. The follow-up experiments are excellent. The new results regarding a magenta enhancer gene, and a set of displaced clines, are convincing. I have no hesitation in recommending this for publication.

That being said...some classical issues on how to describe concepts in this field are not dealt with well. Further, there is one aspect of the new approach which would benefit from clearer explanation...”

I find the author’s responses to my review and three others calm and well justified. I am shocked that the PLoS Biology decision claimed an equal split in the reviewer’s opinions, given that Reviewer1, point2 (scholarship!) demonstrated Reviewer1’s opinion on this topic is of little or no relevance due to their lack of grasp of what a genome-scale cline fitting study is. As an aside, I also (independently of the authors) checked Reviewer1’s point2 ‘supporting’ references and found them entirely inappropriate, and possibly hallucinatory.

There IS a computational problem with maximum likelihood cline fitting. Software that fits only two cline parameters (eg genomic clines) is already error prone because it is under- parameterized (Baird and Daley). Increasing the number of parameters to 4 to reduce such errors will make such software un-usable at genome scale, because it is already almost unusable with 2 parameters, and convergence times explode with further parameters. In contrast, the authors’ work allows for 4 parameters without an optimization step. That resolves a huge computational issue by a qualitative step forward. Reviewer 4 and I agree on these facts. It is no coincidence that we both have hands-on experience with cline fitting.

The authors responded very positively to my suggestions for further clarification of the manuscript. This involved tiny changes at many places – fiddly work mostly well implemented. There remains work to be done, however, as the agreed changes have not been implemented in a quite large section of the manuscript (roughly L225-435).

We really appreciate Stuart’s efforts in reviewing this MS twice, and we apologize for making him highlight the same issues more than once. We’ve made sure that we have addressed every point in this revision and have found his specific suggestions very useful.

Typos/suggestions

Online Abstract:

“Clines at RUBIA approached towards fixation on magenta side of the hybrid zone, whilst remaining polymorphic on the yellow side” -> “Clines at RUBIA approached towards fixation on THE magenta side of the hybrid zone, whilst remaining polymorphic on the yellow side”

Done

Manuscript:

L211 “…to estimates and centre positions across the transect.” -> L211 “to estimate widths and centre positions across the transect.”

Done

L211-213 “Cline widths ranged from ~12.5km to 0.8km, narrowing as the cline centres moved either side of the hybrid zone (< 8km and >15km) (Fig 3).” ->
“Cline width estimates ranged from ~12.5km to 0.8km, narrowing as the cline centres moved either side of the hybrid zone (< 8km and >15km) (Fig 3).”

[The author’s state these changes are artifactual, ie it is not the width per se which is suggested to change, but rather the FastCline estimate of the width]

Done

L221-225 “Cline estimates become more difficult to distinguish as the underlying cline properties become more similar, loci become less differentiated on either side of the cline (S1 Fig and S2 Fig), whereas decreasing sequencing depth and variance in allele frequencies makes these estimates coarser.”

A contrast ‘whereas’ is drawn between aspects of similar effect. First, to say that more similar clines are more difficult to distinguish is a tautology. Second, less differentiation and decreased sequencing depth both make cline more difficult to distinguish. Finally: local variance in allele frequencies increases with cline width, and so is not independent of the other factors being discussed.

Good points. This section has been completely re-written to improve precision and clarity.

L243-245 “FLA1 refers to the cluster of loci on one side of the gene situated > 13km centre positions, and FLA2 refers to the cluster of loci on the other side of the gene with < 13km centre positions.”
->
“FLA1 refers to the cluster of loci on one side of the gene having centre positions > 13km, and FLA2 refers to the cluster of loci on the other side of the gene with centre positions < 13km.”

Done

L258-259 “For 391 clinal loci centred closer to the core region near the phenotypic centre”
[core geographic region, as previously discussed]

Done

L263-264 “In this region”
[genome region, as previously discussed]

Done

L278-280 “The ability of steep clines using FastClines to detect other major colour loci depended on where the cline centre was located. Cline centre displayed a range of positions either side of the main phenotypic transition. Cline centre estimates were spread on either side of the main phenotypic transition, with a bias toward the eastern (Magenta) side (n=1834 vs n=391 near phenotypic centre).”

[This section is a hangover from the previous round of review. The first sentence does not parse. The remainder is repetitive. I think the authors want to say something like:

“The ability of FastClines to detect other major colour loci depended on their cline centre estimates, which showed a range of positions either side of the main phenotypic transition, with a bias toward the eastern (Magenta) side (n=1834 vs n=391 near phenotypic centre).”]

Much clearer. Done

L300 “Given the tendency for widths to narrow”
->
“Given the tendency for width estimates to narrow” [See passim]

Done

L314 “Similarly, in the core region” -> “Similarly, in the core geographic region”
[core geographic region, as previously discussed]

Done

L308-316 “For example, filtering only for loci with centres located in the yellow flanks of the hybrid zone between 8-11km, the steepest clines in this region were all clustered around a narrow genomic region on chromosome 5, which we term Chr5CC (Chromosome 5 Cline Cluster). Clines within this region displayed an average width of 7.52km (±1.04km SD), substantially narrower than the average of other clines in this region at 10.24km (±0.91km SD)". Similarly, in the core region, the CRE locus involved in yellow pigmentation displayed an average cline width of 7.52km (±1.04km SD), substantially narrower than the other clines in this region 10.24km (±0.91km SD) (Fig 4B).”

The confusion over “regions” highlighted in the previous round of review has not at all been addressed here despite author response “We follow this advice throughout and use the terminology ‘geographic regions’ and ‘genomic regions’ to clarify the differences throughout the new version of the manuscript.” Above, the first sentence describes a focus on those clines with centre estimates falling in a geographic region (8-11km). The first use of “region” refers in fact to this focal set of clines. The next two “regions” refer to genomic region Chr5CC. One sentence later we have “Similarly, in the core region…” but this refers to a geographic region, the “core geographic region” defined (L258-269) as 11-14kms. In between we have this sentence

“Clines within this region displayed an average width of [X], substantially narrower than the average of other clines in this region [Y]”

What is the distinction between the two sets of clines ‘within this region’ whiches their difference in width interesting to the reader? The confusion is heightened because clines are referred to as “in” the core (geographic) region. They are not. Their centre estimates fall in the core geographic region. A cline can be described as “in” a genomic region, if that is where the SNP showing the cline is located. A cline cannot be described as “in” a geographic region because by definition clines span all the geography. I think the authors want to say something like:

“For example, considering only loci with centre estimates located in the yellow flanks of the hybrid zone between 8-11km, the steepest clines were all clustered within a narrow genomic region on chromosome 5, which we term Chr5CC (Chromosome 5 Cline Cluster). Clines within this genomic region displayed an average width of 7.52km (±1.04km SD), substantially narrower than 10.24km (±0.91km SD), the average width of other clines with 8-11km centre estimates. Similarly, in the core geographic region (11-14kms), the CRE locus involved in yellow pigmentation displayed an average cline width of 7.52km (±1.04km SD), substantially narrower than 10.24km (±0.91km SD), the average width of other clines centred in this geographic region (Fig 4B).”

We apologise for not adopting the suggested terminology throughout. This was an oversight during revision. We have now followed the above suggestion and corrected this issue with the duplication of values

However, once the results are clearly labelled (like this, or any other clarification), we see a further problem: The average cline widths are copy-pastes of each other! ie 7.52km vs 10.24km for two different genome regions (Chr5CC vs CRE) and two different cline subsets: those with centres in 8-11km vs centred in 11-14km. This is a key passage of the results. Ignoring requests for clarification here is pure self-harm.

This is just a copy paste error that probably arose during revision. We have deleted this section, and the CRE locus is described earlier in the paragraph.

L317 “doesnt” -> “doesn’t”

Replaced with “does not”

L332-335 “The exception was the SULF gene interval (on Chr 4) which contained loci considerably steeper for the KASP site (with MLE width estimates) compared to the majority of poolSeq sites (with FastClines width estimates).” Loci are not steep. The gene interval contains KASP SNPs with steeper clines.

We have revised this sentence to improve accuracy and clarity

L336 “from SULF region” -> “from the SULF region”

Done
 
L336-341 “In comparison to cline widths, the range of cline centres from FastClines was overestimated compared with MLE cline fits. The latter method of cline fitting showing a much tighter grouping of centres much closer towards the phenotypic centre (Fig 3). However, the step change in cline centre positions either side of the FLA gene were consistent between both cline fitting approaches (FLA1 locus and FLA2 locus on Fig 3).” The first sentence is not relative to cline widths, it is a comparison of centre estimate ranges. I think the authors want to say something like:

“FastClines centre estimates had a wider range than those from MLE cline fitting. MLE cline fitting showed a much tighter grouping of cline centre estimates around the phenotypic centre (Fig 3). However, the step change in cline centre positions either side of the FLA gene were consistent between both cline fitting approaches (FLA1 locus and FLA2 locus on Fig 3).”

Again this is a crucial result for those interested in sources of error when estimating cline properties. Clarity is important.

Agreed, we have revised this section to improve clarity

L345 “Divergent loci (∆ ≥ 0.9)”
[Does the ‘divergence’ of a SNP depend on its allele frequency? I think not. I think what is being referred to is lineage sorting and/or diagnosticity]

We have changed th eworking to “Loci with a large allele frequency difference”

L345-346 “Cline width ranged from steep to shallow”
[This despite author response “We reword to only use the term ‘wide’ rather than shallow throughout manuscript.”]

Appologies for missing this during revision. Done

L345-347 “Cline width ranged from steep to [wide] across all chromosomes (Fig 5A), whereas cline centre tended to display different values across chromosomes (Fig 5B).”

Again ‘whereas’ is being used to highlight a non-existent contrast: The text states ‘widths were variable whereas centres were variable’ (across all chromosomes).

We have revised this highlighting that cline widths varied broadly within all chromosomes whereas variation in the centres tended to be distributed among chromosomes.

L358 “clines are often localized” -> “clines are often clustered”
[They are always localized – their positions are known]

Done, while also clarifying that loci are clustered in the genome

L376-377 “Divergent loci exhibiting clinal properties (centres 8 - 16km, excluding step changes over mountain pass)”

[Explain to the reader why clines centered on the mountain pass were excluded, considering we expect HZs to be centered exactly on such barriers/density troughs… without explanation this decision just looks very weird]

We have now made the reasoning for this much clearer, and have also further contextualised the gap in the distribution in more detail at several locations in the results and discussion.
 
L382-385 “Geographic clines, irrespective of properties, were largely clustered in close proximity to the six known genes involved in regulating flower colour. The majority, n = 2918 (76.2%) of divergent loci with clines were located within 300kb of known colour genes (Fig 5).”

[Again an issue from previous review unresolved. The text makes a distinction between ‘divergent loci’ with versus without clines, but the criteria for this distinction are nowhere described. A flat line is simply a cline of great width. Unless the criterion is clarified, the following text is wierded, for example (L383) “Dense clusters of clines are found in small genomic regions” should be ‘dense clusters of CRITERION=STEEP clines…”].

We have revised this, highlighting what we mean here is highly differentiated loci with clines centred between 8 and 16 km. We have revised the rest of the paragraph to clarify that all loci mentioned in this paragraph meet these criteria.

L394 “To understand how pair-wise relative differentiation (FST) associates with colour loci…”
[Tell the reader what the pairs are. In this study the pairs are tables of gene frequency estimates taken from the sets of arbitrary ‘locales’. The reader should be made aware that different (arbitrary) choices of locale boundaries will give different Fst estimates, as this informs us of sources of uncertainty within the Fst comparisons made. this is one of the key advantages of FastClines over Fst scans: To calculate Fst as described here one has to make a decision about what ‘populations’ to compare… especially in a hybrid zone, those decisions are both arbitrary and consequential].

We agree that more information about the nature of the Fst estimates and fundamental difference with cline analysis helps to contextualise the results. We have added several sentences to improve this

L398 “Comparing all nine pair-wise populations of yellow and magenta pools…”
NO the pools ARE the ‘populations’ analysed with Fst.
-> “Considering all nine pair-wise Fst comparisons of yellow and magenta pools…”

Done

L398-400 “…yielded 481 to 694, 10kb windows with excess FST, of which zero to three windows overlapped with colour genes.”
-> “…yielded 481 to 694, 10kb windows with excess FST.”

Done

[(1) an overlap of zero to three is meaningless without context. The text goes on to describe the actual pattern.

Agreed that this is not very meaningful, so we have just deleted this part of the sentence

(2) remind the reader how ‘excess FST’ is defined]

This is given just a few sentences above, so we have not added this definition again, but we have taken it out of parentheses and expanded the definition to make it much clearer.

L405,418 “Some windows of excess FST were identified even between comparisons of two pools from the same variety”
NO. Fst is not measure between comparisons….it IS the comparison.
-> “Some windows of excess FST were identified even when comparing two pools from the same variety”

Done

L419-421 “Comparing with more relaxed outlier cut-off for filtering 10kb windows with excess FST with 95th% quantile, improved the number of outlier windows in each colour gene region (S14 Fig)”

(1) The reader has not been reminded what the other cutoff was, so reporting this one is of low information. (2) The number of outlier windows is increased, not improved. (3) There will be a trade-off with relaxation of the (arbitrary) choice of outlier threshold – this appears unreported, but is why ‘improvement’ is not the appropriate word – the trade-off will not be an improvement.

Yes, the original phrasing is quite sloppy and we have re-written it completely. On point 1, we have indicated what the criteria was relaxed from and to.

L424 “many FST outlier windows contain no clines.” A flat line is a cline of large width. As above, such statements make no sense unless the criterion for a pattern of variation to be described as ‘is/is not a cline’ is made explicit.

We have clarified this in the text and in previous sections by defining clinal outliers.

L434-435 “We interrogated a set of clines from Chr5CC showing the steepest widths in the geographic region left of the core around 9-10km centre position with FastClines (Fig 3)” Widths are not steep, they are narrow. Clines can be steep. I think the authors want to say something like: “Using FastClines we interrogated a subset of the steepest clines from Chr5CC centered around 9-10km ie left of the core geographic region (Fig 3)” Then, again, clarity highlights a further issue: The geographic region is a new one: 9-10km versus 8-11km mentioned earlier.

We have revised this whole section to improve accuracy and clarity.

L473-474 “As in b, the flowers were ranked” -> “As in (B), the flowers were ranked”

Done

L489 “predicts that RUB genotype” -> “predicts that the RUB genotype”

Done

L494 “over the pass” [Remind the reader of the geographic interval of the pass]

Done

L574-575 “heterozygosity is accumulated in a small geographic region towards the sampling edge, generating what resembles narrow clines.”

[While it is nice to see some explanation of this effect, this explanation likely leaves the reader more, rather than less, confused. What biological phenomenon would leave heterozygosity accumulated at the edges?]

We have explained that, in our case, this was routinely caused by the western-most pool showing a large allele frequency difference from the other 5 sampled pools, likely owing to the mountain pass which acts as a geographic barrier.

L580-582 “inspection of individual allele frequencies also show many of these loci exhibit sharp allele frequency step changes between pool YP4 and YP2 rather than a sigmoid-like change over geographic space.”

[A step between two discrete localities is consistent with an (intermediates unobserved) steep sigmoid change. As before, theory would predict a tension zone would become trapped at such a density trough/barrier to gene flow – why no Discussion of this?].

Density traps now explicitly mentioned.

L593 “(i.e. >100kb away from known genes)” -> “(i.e. >100kb away from known colour genes)”

colour

L607-608 “Considering the age of the hybrid zone is at least 100 generations old [27], even the most weakly selected colour loci should have reached equilibrium.”

Not local linkage equilibrium. That has half life around 10,000 generations at 10kb distance. See eg the authors’ own text at LL621-622.

We have clarified that it is the cline width that should have reached equilibrium

L622-623 “For a barrier locus, lower recombination rates increase the extent of linked selection on neutral loci,”

As one of the current authors previously made very clear (Stankowski, Chase et al. 2019), calling indirect selection ‘linked selection’ is unnecessarily confusing, especially in a hybrid zone where admixture LD facilitating indirect selection is generated genome wide… and so has nothing to do with loci being linked. See also (Baird & Daley).

Changed to ‘indirect selection’ as used by Stankowski et al.

L626-629 “Theory predicts the effects of linkage around a single site to be more localised than for a region that contains two or more tightly linked barrier loci, as multiple loci generate a stronger barrier [27] and hence influence the number of clinal loci surrounding the causal loci.”

NO. Linkage is not what generates multilocus barriers. In the absence of LD, linkage has no effect whatsoever.

Theory predicts the effects of indirect selection around a single site to be more localised than for a region that contains two or more tightly linked barrier loci, because recombination will decay admixture LD less effectively in the multilocus barrier region. Then, indirect selection will lead to a stronger barrier in the multilocus case, affecting more of the genome.

This is an important change and has been implemented

L646-647 “This is one limitation of the FastClines method as it assumes a polymorphic yet symmetric sigmoid cline,”

Different levels of fixation (ILS) are allowed in the tails…this is cline asymmetry.

We have changed this to say that fastclines assumes that clines have sigmoid rather than stepped shapes.

L749 “descriptive clines” this despite authors’ response ‘Removed the term descriptive throughout manuscript’

Done

L763 “gaussian cline” [Check this?… I think the result is for a logistic cline. Yes, its definitely logistic, see LL800-801]

Done

L859 “Prior to commencing cline fitting algorithm” -> “Prior to commencing the cline fitting algorithm”

Done

L865-866 “We begin the algorithm with a random set of parameters which are changed randomly…” [repetition]

Done

L960-961 “The location of all known genes known to influence flower colour” [repetition]

Done

Reviewer #2: I reviewed the manuscript by Field et al. exploring the indirect signature of reproductive isolation on genetic diversity using an exhaustive genome scan of allele frequency clines in the classical snapdragon color-morph hybrid zone. The study demonstrates that using a relatively small number of pool-seq samples and a novel procedure for allele frequency cline fitting based on expected total heterozygosity efficiently captures the main reproductive isolation (RI) loci in the species. The authors also describe a new color locus affecting the intensity of magenta pigmentation. The robustness of the clines fitting and the new RI locus detected was further validated by looking at some KAPS sequencing over a large number of samples.

First, I must apologize for the delay. When I first downloaded the manuscript, I apparently received a previous version (e.g., some characters were overlapping in Figure 2, and I had no access to the previous reviews from the PLoS Biology submission). Consequently, I commented on issues that I later noticed had already been addressed in the current submission (such as the benchmarking of the method). Overall, I find the manuscript clear and the method sound, and I have only very limited comments, which I list below. I believe this manuscript will make a valuable contribution to PLoS Genetics.

Minor comments:
L86: "most still use specific loci of interest or sparse reduced representation genomic data" This sentence is somewhat misleading because the present manuscript also applies a stringent threshold (Dfreq >0.9) prior to performing cline fitting, which similarly reduces the genomic representation being analyzed.

We can see why the reviews was bothered by this. Weve simply removed this sentence, an the text now reads:

There is a long history of fitting geographic clines to small numbers of loci to estimate selection in hybrid zones [10,13] with increasing numbers of loci available as sequencing technologies improved [e.g. 14,15]. ~~Although a few studies have begun scaling up to many thousands of loci, most still use specific loci of interest or sparse reduced representation genomic data~~ [16,17]. As the field moves toward whole genome resequencing data [18], the increasing computational burden of fitting complex cline models to large numbers of loci requires the development of new tools for efficient genome wide cline estimation.

Fig3: When examining the frequency variation of Chr5CC shown in Fig. 3B, the most differentiated loci appear to be very close to the Dfreq threshold of 0.9, yet this region shows extreme outlier behavior in terms of cline width.
I wonder how much the arbitrary threshold of 0.9 affects the power to detect regions under selection? In addition, I would like the authors to justify a bit more on the choice of the DFREQ of 0.9, which I find quite stringent. Others sometime use Dfreq > 0.3.

Specific comment/
L115: I would recommend providing some information here about what this new method entails and how it differs from existing approaches.

We have added one extra sentence to the abstract to help forshadow this.

I suggest providing a brief explanation of total heterozygosity and how it relates to the 4pq formula.

We have expanded this sentence to give a clearer explanation of the relationship.

L209: Same line: maybe the bracket could mention the benchmark, and it could be interesting to mention how much faster does the cline fit procedure is at this stage.

Weve not added information on benchmarking here, but we now explicitly direct the reader to the methods section for these details.

L392-426: I am not fully convinced of the added value of comparing window-based FST with SNP-based cline analyses. Moreover, I think a comparison between the ARG-based inferences/blocks inferred from the ARG (recently published in Molecular Ecology by the same authors) and the cline results would have been more interesting.

We think the Fst comparison is important, because Fst remains the bedrock of most speciation genomic studies. People have a good intuition for Fst, and we think that most people would want to see this comparison, which also helps to highlight the strengthts of geographic cline analysis.

It would be cool to compare the clines to inference from ARGs, we can’t do this very well in the current manuscript, because we can’t infer ARGs from poolseq data.

Fig 5: It is not immediately clear to me why the x-axis labels in the first two boxplots differ from those in the second panel, nor why flanking regions are not shown for all genes. Does this relate to the deltaP > 0.9 threshold, where sometimes no clinal markers were found to the right or left of the selected gene? Ultimately, the figure is somewhat difficult to read, primarily because some categories are missing and the reason for their absence is not obvious. A brief explanation in the figure legend clarifying which categories are excluded and why would improve readability.

We have added a small explanation to the caption.

L501: Should this be "RUBPS" like in L511 rather than "RUBP"?

We have changed RUB^p to RUB^ps, which is correct

L511 : This sentence seems oddly placed given that the two alleles have already been presented above. Should it appear earlier in the text?

Yes, this sentence makes more sense in the paragraph above for we use the superscripts to denote the pseudomajus and striatum alleles.

Reviewer #3: The authors have addressed the major comments from the previous submission, and this has shaped up to be a solid contribution.

As a reviewer, not a co-author, I cannot force the authors to write a different paper. But I still believe that t the biological advance here is modest, and that a stronger contribution would have been a more weighty methodological contribution. it seems like the authors decided against that. Because this paper is so polished, and because this is the paper the authors are going to write, it is hard to know what to do with this, but I am find myself suggesting acceptance at PLoS Genetics. However, I would understand if other reviewers or the AE suggest PLoS one (although this is way more substantive than a typical PLoS One paper) or G3 (which seems like a more natural home).

We have decided to keep the emphasis on the biological story and following the advice of the editor we increased the discussion of our findings in this respect.

**Have all data underlying the figures and results presented in the manuscript been provided?**
Large-scale datasets should be made available via a public repository as described in the *PLOS Genetics* [data availability policy](https://url.au.m.mimecastprotect.com/s/FX0iC0YKgRsGyPnxxc2iwF9kTn5?domain=track.editorialmanager.com), and numerical data that underlies graphs or summary statistics should be provided in spreadsheet form as supporting information.

Reviewer #1: Yes

Reviewer #2: Yes

Reviewer #3: Yes

PLOS authors have the option to publish the peer review history of their article ([what does this mean?](https://url.au.m.mimecastprotect.com/s/7KROCgZ05JfABQv33h3syF4twZP?domain=track.editorialmanager.com)). If published, this will include your full peer review and any attached files.


If you choose “no”, your identity will remain anonymous but your review may still be made public.


**Do you want your identity to be public for this peer review?** For information about this choice, including consent withdrawal, please see our [Privacy Policy](https://url.au.m.mimecastprotect.com/s/YjkeCjZ12RfnONzrrI1tqFmiCDo?domain=track.editorialmanager.com).

Reviewer #1: **Yes:** Stuart J.E. Baird

Reviewer #2: No

Reviewer #3: No
