# Supplementary material for: Genome-wide cline analysis identifies new locus contributing to a barrier to gene flow across an Antirrhinum hybrid zone
Source: PLoS Genet. 2026 Jul 13;22(7):e1012173. doi: 10.1371/journal.pgen.1012173 (PMC13387609; doi:10.1371/journal.pgen.1012173)
Supplement: Attachment — Submitted filename: Prior Peer Review and Response letter FieldEtal 21_10_2025.docx [file pgen.1012173.s032.docx]

Dear David,

Thank you for your patience while your manuscript "­­­Genome-wide cline analysis identifies new locus contributing to a barrier to gene flow across an Antirrhinum hybrid zone" was peer-reviewed by PLOS Biology. Your work was assessed and discussed by the PLOS Biology editorial team, an Academic Editor with relevant expertise, and by four independent reviewers. Based on the reviews, which you will find at the end of this email, I regret that we will not be pursuing your manuscript for publication in the journal.

I'd like to apologise for the extraordinary time that it's taken us to secure this decision; the process has been delayed at multiple stages, and although this was usually for reasons outside our control, I wish that we had been able to deliver an outcome in a more timely fashion, especially as it's not the answer that you would've hoped for.

You'll see that reviewer #1 thinks that the methodological advance is modest, as the computational burden of current methods is not substantial. S/he also thinks that you ignore some of the prior literature, and questions the assumption that clines should always entail barriers to gene flow. Reviewer #2 is also negative; like rev #1, this is on the grounds of advance, as s/he doesn’t think that the approach is of broad interest; to improve it methodologically, you would need additional benchmarking against existing methods. Reviewer #3 is very positive; his/her major points seem to be semantic, followed by a long list of minor issues. Reviewer #4’s review is very positive, with no real requests.

Clearly there is a split among the reviews, which remained even after cross-commenting between the reviewers, so we had an extensive discussion with the Academic Editor. In the end, both we and the AE were persuaded by the more negative assessments of reviewers #1 and #2.

I'm sorry that we cannot be more positive on this occasion and hope the reviewer reports will help you in preparing your manuscript for submission elsewhere.

While we cannot consider your manuscript for publication in PLOS Biology, we suggest that you consider transferring your manuscript to PLOS Genetics. However, please note that the PLOS journals are editorially independent, so we cannot guarantee it will be reviewed there.

If you would like to submit your work to PLOS Genetics, please click the following link:

[https://www.editorialmanager.com/pbiology/l.asp?i=1171869&l=O3EVJ33I](https://url.au.m.mimecastprotect.com/s/JFOHC91W8rCkZ0VyOSQIKiqoNhc?domain=editorialmanager.com)

If you do not wish to submit your work to PLOS Genetics, please click this link to decline:

[https://www.editorialmanager.com/pbiology/l.asp?i=1171870&l=3ROQNJBE](https://url.au.m.mimecastprotect.com/s/aJxzC0YKgRsGEx0OytpSAi9pNfi?domain=editorialmanager.com)

Please note, you can log into the submission sites with the same login that you used to submit to this journal.

Should you choose to transfer your submission, you will receive a confirmation email within 24-48 hours after accepting the transfer. If you have any questions, please feel free to contact the journal at plosbiology@plos.org.

I hope you understand the reasons for this decision and that you will consider publishing your work in PLOS in future. Thank you for your support of PLOS and of Open Access publishing.

Sincerely,

Roli


Roland Roberts, PhD
Senior Editor
PLOS Biology
rroberts@plos.org


------------------------------------------------------------------------

REVIEWERS' COMMENTS:


Reviewer #1:

Review of "Genome-wide cline analysis identifies new locus contributing to a barrier to gene flow across an Antirrhinum hybrid zone"

This is a "Methods and Resources" submission to the journal, but I struggled to find a contribution here that rises to "significantly outperform the state-of-the-art methods or that show the potential to address, for the first time, a pressing biological question". While I also had biological and scholarship concerns, I will begin with the computational issues first.

1. Is there really a computational burden to current methods for fitting cline models, as claimed? Computers are quite fast, and importantly no analysis of runtime or memory requirements are made here. It could be that "FastClines" is needed to analyse whole-genome datasets, but no evidence of this is provided. In fact, <1% of SNPs are even analyzed in this paper, which must make the problem much simpler.

RESPONSE:

Computational burden:

- There is indeed a major computational burden for fitting cline analyses. To understand this, we first need to clarify two different schools of cline methods.
- The first main class of method is geographic clines. We run this analysis in the traditional way (e.g. Syzmura and Barton (1986)) as implemented our MLE approach, with 4 parameters (centre, width, p0, p1) plus Fst.
- The second main approach are the non-geographic or ‘genomic clines’ , which compare genome wide ancestry (Hybrid index) against genotype frequencies at individual loci. First proposed by Syzmura and Barton (1986) and further developed by several papers (e.g.). The latest version of this approach is bgchm (Gompert, DeRaad et al. 2024).
- **Benchmarking**: We now outline in the manuscript some of the benchmark tests in the Materials and Methods line 937
  - “To demonstrate the speed of this new approach, we ran some benchmark tests to compare *FastClines* to MLE cline fitting, running both approaches on a MacBook Pro (2023 model) with 12 core Apple M2 Max chip with 64GB RAM. Running *FastClines* on the *Antirrhinum* poolSeq data with 6 localities used in this manuscript, when searching through whole genome sync files, filtering and calculating cline width and centre, it took ~0.0004 seconds per site (locus), and ~7 minutes for 1 Million sites. In comparison. using our MLE approach (R script) used in the manuscript takes ~25 seconds per locus (for modest number of iterations = 20000 and burn-in = 5000), therefore if we had more KASP loci available (to make more comparable to our poolSeq data), this would be ~ 34 hours for 5,000 loci and ~6800 hours (=283 days) for 1 million SNP loci.”
  - Obviously, MLE cline fitting could be optimised with a faster scripting language and running on a cluster in parallel. Yet, MLE approaches typically involve exploring several alternative model fits (e.g. asymmetric 3-part clines) which means we are also underestimating the time required to run all MLE cline fits. The *FastClines* method circumvents this computational burden, with an alternative estimate of cline parameters that requires no optimisation step to iterate over a likelihood surface like with MLE approaches. This makes it orders of magnitude faster and allows it to run on a standard laptop.
- Now lets compare to non-geographic methods. Although the bgchm method is reported to be appropriate for next generation data, it is not suited to this scale of analyses for true genome wide studies but instead more suitable to a few thousand SNPs. This is already outlined by Gompert, DeRaad et al. 2024, it can in principle be run on more SNPs, however they mention “and we have successfully used this approach to fit clines for >1 million SNPs in about 2 days on a single compute node with 48 CPUs.”. In contrast, *FastClines* takes 7 minutes to compute 1 Million loci, which is ~400 times faster) can be run on a standard laptop (no cluster required), and its also measuring something quite different (geographic genome-wide cline estimates).
- In summary, given the length of time to run two alternative methods (MLE geographic and non-geographic clines bgchm), which can take days to run on a HPC, this in our opinion is a major computational burden. *FastClines* overcomes this computational burden as demonstrated with the runtime benchmarks now provided.

The only small comparison that is provided seems to demonstrate that FastClines significantly underperforms against the authors' implementation of current models (more on this below). So, while I believe the authors have made some contribution with their faster method, there is no support for any claims that it outperforms existing methods, or allows for new insights.

RESPONSE:

- Regarding the claim that we demonstrate FastClines underperforms compared with traditional geographic MLE cline fitting. We compare the MLE likelihood maximisation approach to the FastClines approach which requires no maximisation step.
- This comparison was intended to show the qualitative (not quantitative) gain in speed. Although we now also show the speed difference (See above)
- This is because making comparisons between methods with same dataset is difficult as MLE cline fitting doesn’t work with such low numbers of demes as FastClines (our poolSeq empirical data was 6 demes vs. KASP SNP genotypes has 100s of demes). If we tried to make a fair comparison of the methods, fitting MLE clines only to a trimmed down 6 demes dataset like the poolSeq data we used for FastClines, the traditional MLE method completely fails to converge and cannot estimate meaningful cline parameters whereas FastClines works with as little as 6 demes. This demonstrates another useful feature of the method, reasonable approximations of geographic cline parameters at genome wide scale with limited spatial resolution (i.e. 6 sampling localities). FastClines essentially outperforms the traditional methods when few demes/localities are available.
- In summary, FastClines solves a major computational burden by eliminating the search step for computation whilst allowing rapid estimates of 4 geographic cline parameters. This makes it more suitable for rapid truly genome scale analyses compared to either school of analyses. As we demonstrate, it also allows for estimates of geographic cline parameters with only 6 localities of data. Both of these aspects have been reemphasised together with the computational speed in the manuscript on line 937. However, we still view this as a complimentary to available methods, and not a replacement, a already stated in the discussion. More precise estimates of geographic clines at select loci can be examined if required and can be estimated using MLE or non-geographic cline methods

A few related, smaller issues: I didn't understand why it is implied that Metropolis-Hastings and simulated annealing are the same thing (line 827). My reading of the methods suggests that the authors have implemented a standard M-H algorithm. Maybe the acceptance ratio changes over time (which would make it similar, but not the same as, simulated annealing), but this is not described anywhere.

RESPONSE:

- The details of the method are already outlined in the manuscript in paragraph starting on line 988, and more specifics here on L994 "the jump size for the next set of parameters are adjusted by a factor of 1.05 when accepted (accept scale) and by (1/1.05) when parameters are rejected (reject scale). "

In addition, the M-H algorithm is a Markov chain Monte Carlo approach. Normally, such approaches provide a maximum a posteriori (MAP) estimate, not a maximum likelihood estimate (MLE), though this doesn't always need to be the case. The authors should clarify throughout what kinds of estimates they are making, and what kinds are being made by existing software.

RESPONSE:

- The methods normally applied in geographic clines analyses are Maximum Likelihood Estimation (MLE) and described clearly on line 988. These MLE approaches have been widely used for over 30 years (since Syzmura and Barton 1986) and are not MAP estimates. The method is already clarified is on line 988 and in supplementary S2, and is the same method described in detail in our previous publications Tavares et al. 2018 PNAS and Bradley et al., 2017 Science.

2. Scholarship-wise, it was a bit surprising that the paper referred to clinal analyses of hybrid zones as "promising" with the "move towards" genomics. There are in fact quite a large number of studies already doing genome-scale analyses using the classic approach to clines used here. While some (but not all) use ddRAD sequencing approaches, they still have many thousands of markers.

A non-exhaustive list of references:
[https://doi.org/10.1073/pnas.2015005118](https://url.au.m.mimecastprotect.com/s/ai6RCgZ05JfAg35XBHxT8i4FZ6W?domain=doi.org)
[https://doi.org/10.1371/journal.pgen.1011655](https://url.au.m.mimecastprotect.com/s/ToC2CjZ12Rfn6r1LOCJU9imSQr6?domain=doi.org)
[https://doi.org/10.1111/evo.14001](https://url.au.m.mimecastprotect.com/s/poA4Ck815RCOLE3VwUzcxiG4Oww?domain=doi.org)
[https://doi.org/10.1111/evo.13675](https://url.au.m.mimecastprotect.com/s/Y0MiClx1OYU29rqJMt4f3izm0n0?domain=doi.org)
[https://doi.org/10.1111/mec.14236](https://url.au.m.mimecastprotect.com/s/BMv9CmO5wZsj0xLwMhlhAiR8Myx?domain=doi.org)
[https://doi.org/10.1038/s41437-023-00617-6](https://url.au.m.mimecastprotect.com/s/qrz6Cnx1Z5U7yEp0Wtri2iJA7QL?domain=doi.org)

RESPONSE:

Scholarship:

- We completely disagree on this criticism of scholarship and claims of a “There are in fact quite a large number of studies already doing genome-scale analyses using the classic approach to clines”. The studies they provide do not support their claims, every example provided are either (i) not genome-scale analyses (ii) nor using classic geographic approach to clines
- Of the six examples provided, the only study which infers parameters for many clines is a 2 parameter NON-geographic analysis with a maximum 1141 RAD SNPs, which is hardly genome wide.
- The studies provided are by no means genome wide, and none contain geographic clines which is the relevant comparison to FastClines presented here.
- Here is the comprehensive detail of each paper listed

1. <https://doi.org/10.1073/pnas.2015005118>

- A two parameter analysis (non-geographic) analysis of just a few clines:  see section "Cline Analysis" : "For each trait (wing color pattern) or locus, changes in allele frequencies across the cline were analyzed to determine the best-fitting cline model, typically with two parameters, cline centers and widths"

2. <https://doi.org/10.1371/journal.pgen.1011655>

- This paper does look at millions of SNVs but no cline fitting at all was performed in the study!

3. <https://doi.org/10.1111/evo.14001>

- This one does analyse classic geographic clines, but only 2 clines fitted (not 1000s or millions) see "The hybrid indices for each transect against their point-to-point distance were fitted to a series of equilibrium geographic cline models using the Metropolis-Hastings Markov chain Monte Carlo (MCMC) algorithm employed in the R package HZAR"

4. <https://doi.org/10.1111/evo.13675>

- A two parameter non-geographic **and** only one cline fitted! See this section of study "using the Metropolis–Hastings Markov chain Monte Carlo algorithm in the R package HZAR (Derryberry et al. 2014). To evaluate the genetic center and width (1/maximum slope) of the hybrid zone, we estimated clinal transitions of Q from STRUCTURE for *K* = 2 for all genetic samples collected from 2010 to 2014.

5. <https://doi.org/10.1111/mec.14236>

- This is a non-geographic analyses, and only 230 clines fitted (not thousands or millions). Look at this section: “We evaluated coincidence (amount of congruence among estimates for cline centre for each SNP) and concordance (amount of congruence among estimates for cline width for each SNP) using the RAD2 data set for ddRADseq loci identified as outliers that were in HWE in each location and filtered to 1 SNP per 1 kb (228 SNPs), as well as mtDNA (COI) and Z-linked (Ldh) loci.”

6. <https://doi.org/10.1038/s41437-023-00617-6>

- This is the only one of these examples where many clines are fitted although still modest in number. It is non-geographic approach, using BGC (2012 forerunner to  bgchm).
- The number of clines fitted is not stated clearly, but sites are clearly heavily filtered down from a maximum of 1141 RAD SNPs.

3. As a general biological comment, I did not always understand the framing of clinal analyses as finding "barriers to gene flow" and reproductive isolating loci. The two color-morphs studied here (which are two varieties within a sub-species) don't have any obvious reproductive isolation, just geographic separation. The study therefore seems more akin to a case-control study for a trait that is not randomly spread across the environment. This doesn't mean the method doesn't find loci underlying the trait, only that the interpretation of cline width and slope might mean something quite different from a true hybrid zone between species.

RESPONSE

- This is an interesting comment, geographic clines in hybrid zones are often considered to arise due to a balance between dispersal and selection, and therefore loci linked to traits under selection due to involvement with reproductive isolation would represent barriers to gene flow (whereas other regions not closely linked to such loci homogenise). Perhaps the reviewer is considering the possibility of a transient cline upon secondary contact, no selection required (as per the paper they raise Jofre & Rosenthal 2021). There is two lines of evidence why this can be refuted: first, very non-homegenous genomic patterns - just a few steep clines linked to colour traits, while most of the genome is homogenised.

If we consider a cline linked to a trait under selection, any cline maintained by selection must reduce gene flow at linked loci (see multilocus cline theory from Barton 1983). Regarding the geographic separation and varieties that hybridize, this does demand explanation and there are two explanations:

(a) The two varieties met recently and this is why they haven’t homogenised
(b) Homogenisation is selected against (barriers to gene flow)

The first one is unlikely given the homogenisation (panmixis) over most of the genome (see also Tavares et al., 2018), which implies they met a long time ago and intermingled their genomes. A few regions with barriers and linked regions around them remain.

Regarding the idea this is a case control study – we are agnostic to what level of taxonomic classification one would like to attribute to these varieties. Some call them subspecies, others varieties. We have evidence of lower fitness of hybrid phenotypes (not presented here), and the fact that these colour traits are determined by the loci with the steep clines, that are maintained (cant penetrated far into alternative background) after rest of genome has homogenised is supportive of selection at these genomic regions.

In addition, the authors should read and possibly cite Jofre and Rosenthal (2021). There are many caveats to interpreting clinal analyses in general that are not addressed here.

- We thank the reviewer for raising this paper. We have now cited it in the challenges for genome wide cline estimations across genome data outlined in the introduction here:
  - L111: “However, the distribution of genome wide cline properties will depend on several parameters that may be difficult to measure in nature, including: the time since secondary contact, neighbourhood size and drift, genome wide barriers, epistasis and local recombination rate”

4. I did not understand how loci with steep clines don't show high Fst. Wasn't the first step in the clinal analysis to find loci with large differences in allele frequency? Shouldn't that have led to the ascertainment of loci with high Fst necessarily? I must be missing something.

RESPONSE:

- It has been widely accepted in speciation genomics that high Fst (i.e. genomic islands of differentiation) is a poor indicator of geneflow (and barriers) because it confounds gene flow with diversity within populations. Hence, drops in diversity due to historical taxa specific sweeps, linked selection in low recombining genomic regions can all produce high Fst. This has been widely published and discussed, dating back to Charlesworth in 1998 and comprehensively dealt with by Cruikshank and Hahn in 2014.
- A few loci (with strong allele frequency differences) are not necessarily sufficient to raise Fst, as the Fst data is whole genome here and metrics in sliding windows of 10kb, whereas clines are estimated at individual loci (sites). In addition, there are other regions of the genome with high Fst yet no clines (e.g. centromeric), driven by low diversity in both populations but not high divergence (Dxy). Given high Fst can arise from low diversity alone (see first point), clines and Fst are not the same measures and we don’t expect them to align either.
- Lastly, genomic islands of differentiation (i.e. Fst islands) do not have an explicit geographic component. One can show pair-wise Fst but many pairwise comparisons do not provide the same explicit geographic estimate as do clines across a transect.

Also, you may not want to call clinal analyses "more robust" than Fst analyses (line 32) without further evidence or explanation.

RESPONSE:

- See above comment. There is plenty of evidence presented in this manuscript and number other studies showing the flaws in using Fst to detect barriers, and how more robust clines are to Fst.
- We have added an addition sentence in the introduce to highlight the re-analysis paper of Cruikshank and Hahn, which provided a broad set of comparative analysis evidence that Fst is not robust measure for detecting barriers to gene flow: “Lower diversity within populations due to species specific sweeps, and linked selection in low recombining genomic regions can produce excess *F_ST_* without any role of differentiation gene flow”

5. Minor comments:
-Refs 7 and 37 are the same.
-I don't think ref 38 is the correct reference, given the context within which it is cited in the main text.

- Corrected, removed duplicate copy of Cruikshank and Hahn reference
- Ref 38 – this is the correct reference for eggNog

Reviewer #2:

Review of "­­­Genome-wide cline analysis identifies new locus contributing to a barrier to gene flow across an Antirrhinum hybrid zone"

This paper is remarkably thorough and well considered and well executed. I have no methodological critiques or real misgivings a bout anything accomplished here. My biggest concern / critique of this fine manuscript is that it wasn't clear what the major contribution was. I have a few ideas about what it may have been, but I'm not sure if any individual make this a paper of broad interest. Another way to think about this is "why would someone cite this paper?" Here are some potential reasons

1. High quality analyses. The work is notable for being remarkably well executed. Sample sizes are large, sampling is solid (although I imagine finer-grained sampling could have helped more), analyses are sophisticated. This is just well done. However, it is not clear that such solid science, moves a paper to PLoS Biology rather than e.g. G3

RESPONSE:

- We appreciate the reviewers positive comment on the quality of the work.

2. Methodological advance. The authors adapted geographic cline fitting algorithms to deal with the scale of modern data. This is a nice contribution. But I would imagine a more worked up bioinformatics note with stats about performance, speed, an easy to use package etc, and other standard benchmarks would be necessary for this to be the major contribution.

RESPONSE:

- We appreciate the reviewers positive comment regarding the nice contribution of the FastClines method. We have followed the reviewers suggestion and now include some standard benchmarks on line 988. The package is at this stage in a very useable Python script with instructions and guides on Github. Although we appreciate the comment about having an easy to use package, this is a beyond the scope of the current paper and we will develop the package further with new features for future versions and possible bioinformatics note. At this stage we want to publish the theory and the biology so its also usable now. Its common for pop genomic analyses to run through script format and run through command line as it’s the format to run analyses on clusters and HPC. Moreover, because this method is a qualitative change from traditional MLE clines, exhaustive benchmarking is not particularly insightful (i.e. FastClines has no optimisation routine and is a very different approximation method). In other words, it’s a little like comparing very different methods approaching the problem (estimating clines) in such a different way that we expect dramatic differences in run time.

3. Validation of previous results for the system. This is reassuring, but again does not seem like a result that should attract a general interest.

RESPONSE:

- We appreciate the reviewers positive comment on the validation is reassuring. We think that this should be of general interest. Validating the approach in other study systems was simply out of scope of this manuscript. This comment does somewhat contract the next one below, where reviewer #2 says discovering a novel locus is exciting and general. We consider validating other known genes be of general interest to other authors seeking to find barrier loci and genes in other study systems with this method should be of general interest.

4. Identification of a novel locus. This is the most exciting and general result. But Fine mapping a gene of interest in an ecological system might not be enough for a general contribution.

RESPONSE:

- We appreciate the reviewers find the identification of a novel gene exciting and produces a nice general result. The extent of generality may be up for debate, but we consider finding a novel gene under selection in a hybrid zone to be an important general finding for hybrid zone and speciation research and is rarely achieved in the literature.

In sum, based on the biological contribution, I think this paper is ready to published without hesitation in a solid science journal. But needs to make a much stronger case for a clear biological advance to be of interest to the broad readership of PLoS biology.

Alternatively I noticed that this is submitted as a "Methods and Resources" articles. (Methodological advance). From the PLoS Bio website "Methods and Resources are a broad article type with 2 main subtypes. The first of these presents methodological or technological advances; such methods should be novel and show the potential to experimentally address a previously inaccessible biological question. Alternatively, the methods presented may demonstrate substantial improvements to currently used methodologies. They would need to significantly outperform their predecessors by precision, resolution, speed, accessibility, and/or cost." This may fly into type two, but again (as stated above) if the advance is methodological I would expect much more time devoted to the method, a slick software presentation (rather than a somewhat hidden GitHub link), validation of performance and standard comparisons of spaces / performance and comparison to other approaches.

RESPONSE:

See comments above which address this. In brief, the reviewer agrees the article is ready to be published in a solid science journal and we believe PloS Genetics as an alternative would therefore be suitable.

Reviewer #3:
[identifies himself as Stuart J.E. Baird]

Overview

This is an excellent study applying new methodology to, and reporting new biology of, the Antirrhinum system. The planning, layout and argumentation are lucid. The FastClines approach elegantly plugs an important computational hole in genomic studies of hybridisation. The follow-up experiments are excellent. The new results regarding a magenta enhancer gene, and a set of displaced clines, are convincing. I have no hesitation in recommending this for publication.

That being said…some classical issues on how to describe concepts in this field are not dealt with well. Further, there is one aspect of the new approach which would benefit from clearer explanation. Dealiing with the classical issues first.

(1) Demes.
Antirrhinum is not a demic hybrid zone in the sense of eg a string of ponds. The current Wikipedia definition of deme suggests no change in deme definition since the 1970s, when simulating a (long) chain of demes became the go-to approach for verifying and extending analytical results. Choosing to call sampling localities along a non-demic hybrid zone "demes" therefore risks many levels of unnecessary confusion. For example "simulations of clines with six demes" at L206 presumably are not at all "simulations of clines with six demes", but rather simulations of the effects of sampling a 1D clinal system (perhaps well approximated by a chain of many demes) at only six localities along its length. L846-857 clarifies that this is exactly what is going on. For a further confusion example see L236 "The MLE cline parameter estimates at the four representative loci from the same regions using dense geographic sampling (>100 demes)". If the number of things is about the density of
geographic sampling, then these are sampling localities, not demes. If the 'demes' are actually defined by drawing loops around convenient lumps in the existing map of sampling, and discarding the sampling outside of these loops (as suggested at L715-716) , then just call them 'locales'…you need a vanilla word, not one with 80 years of baggage.

RESPONSE:

- This is a very valuable comment, and we appreciate the reviewers detailed dive into the historical and common use definitions of term ‘demes’.
- Regarding the simulations (L206), indeed this is looking at effects of sub-sampling at locales along an established cline with a continuous distribution, rather than simulating only 6 connected demes. In the simulation approach, as sampling density is increases, it approaches a more deme sampling design.
- We agree with the reviewer definition and replace all mention of ‘demes’ with the term ‘locales’ or ‘localities’ throughout manuscript. Given the number of instances of the use of ‘demes’ in the previous manuscript, we cannot list every change in this response.
- See further response below

(2) Loci.
This word is used very flexibly in population genetics, and I am all for that. But even this heavy lifter can be overloaded. Originally loci were often treated as independent and indivisible, which would make the word largely unusable for admixture genomics, but the latin meaning of loci is just places…places in the genome. The manuscript involves several types of place in the genome: variant sites in poolSeq (L183), KASP markers (L309) KASP SNPs (L314) both of which are just assays of variant sites, and annotation intervals labelled as genes. I think all three are used simultaneously here:
L320-322 "The exception was the SULF locus which was considerably shallower for the locus with MLE estimates compared to the majority of clines from poolSeq with FastClines."
Do the authors think the casual reader would be able to spot which are which? My guess for unloading the overload is:

"The exception was the SULF gene (interval/region) which was considerably wider for the KASP site(s?) (with MLE w estimates) compared to the majority of poolSeq variant sites (with FastClines w estimates)."

Help the reader by unloading some of the load placed on the word "loci'.

RESPONSE:

- We appreciate this comment and follow the reviewer guide for clearing up the descriptors of genomic regions/loci. We followed their suggestion on line 390 with some further modifications:
  - " The exception was the SULF gene interval (on Chr 4) which contained loci considerably steeper for the KASP site (with MLE width estimates) compared to the majority of poolSeq sites (with FastClines width estimates).."
- and also in some other locations in the manuscript, points and responses are below

(3) Clines.
Several things are going on in the previous quote. Cline models are used for both the KASP sites and the poolSeq sites, yet it seems that throughout the results section the word 'cline' is, as above, used to refer only to the poolSeq variant sites.

Unpacking this sentence:

L248-253 "The steepest clines overall were observed for loci tightly linked to ROS and EL, the two major effect loci important for regulating magenta pigmentation (Fig 3; Fig 4). For clinal loci centred closer to the core region near the phenotypic centre for colour (between 11 and 14 km),"

First, for the two major effects, "loci "is I think being used in yet another way: literally as place, ie not connected to any particular site or gene. Second, here "clinal loci" I think refers to poolSeq variant sites, but only those with 'clinal' pattern. In that case: where in the text is the distinction between clinal and non-clinal variant sites defined? Third, the word region has already been used for regions the genome (eg 7 times in the abstract), but here "core region" is used for a geographic region. Unload that to eg "core geographic region". Fourth, this section of the results refers to distances from an arbitrary geographic endpoint. It is more normal in clinal analyses to link estimates of cline centres to either some central geographic feature or some consensus estimate of zone centre. Fig 1 has locality labels beginning MP, and the discussion refers to the Mountain Pass, but the reader does not seem to have the kms interval for the mountain pass, in order to
connect to results such as "between 11 and 14 km" to the "Mountain pass" of the discussion.

RESPONSE:

- Firstly, indeed there is some confusion with this sentence on L248-253. We are talking about steep geographic clines (i.e. width estimates) together with cline centre geographic positioning of these clines in space (either near phenotypic centre or displaced). To clarify we have modified the sentence to:
  - "The steepest clines overall were observed for loci tightly linked to ROS and EL, the two major effect loci important for regulating magenta pigmentation (Fig 3; Fig 4). For clinal loci centred geographically closer to the core region near the phenotypic centre for colour (between 11 and 14 km),"
- Regarding the second question, yes this is only with regard to ‘clinal’ loci. We do go through how many of these and how they were ‘filtered’ (based on allele frequency differences between outer most population localities) earlier on in the manuscript on L197 and in methods on L924. We do actually feed all loci through FastClines, it does the filtering and only calculates cline parameters for a delta p we provide (it was 0.8, but we only show 0.9). However for highly polymorphic loci between the two variants we don’t get any meaningful estimates of cline parameters. In MLE, it would also be typical to filter out loci which do not show some kind of strong allele frequency differences. Another factor with FastClines, if we were to include everything, allele frequency reversals (i.e. highly polymorphic frequencies in space) can give strange artefacts and narrow cline widths. With more pools (locales) these artefacts do diminish (see supporting Info simulations).
- Regarding third comment, nice observation on the term regions. We follow this advice throughout and use the terminology ‘geographic regions’ and ‘genomic regions’ to clarify the differences throughout the new version of the manuscript.
- Regarding fourth comment, this is a good point. We clarify that the cline transect is located around a central geographic feature as defined by the centre of the phenotype cline (see L355: (~1.5 km West of phenotype centre at 12.5km). We also mention the mountain pass km interval in several locations in the new version L185 and L350 and in methods (L829)

When is a cline steep?
Further information is necessary to answer this question, yet in many places where 'steep' is used (noted in the line-by-line below) that further information is absent. The impression given is that the authors really wish the reader to believe in the strength of the cline. But calling it steep in isolation is not the way forward. A cline can have a steep central portion - but this is in comparison to its tails. Selection can produce steep clines - but that is in comparison to the neutral case. A cline can be steep in comparison to the per generation scale of dispersal - but no estimate of that is mentioned in the manuscript, and even the concept is not mentioned other than at line 76, where it appears without explanation. The issue is not just aesthetic. As above, where in the text is the distinction between clinal and non-clinal variant sites defined? Is that by chance decided by how steep they are estimated to be relative to each other?

RESPONSE:

- This is a good point. For this system, the vast majority of geographic clines are found around each of the main colour genes (in tight linkage), and the rest of the genome appears homogeneous with few clines. There are some exceptions, loci with steep shifts in allele frequency over the mountain pass, likely reflecting a physical/geographic barrier to gene flow between yellow locales/populations either side of this physical barrier. This further supports that the yellow and magenta on the same side of mountain are homogenised (except at colour genes acting as barriers in small genomic regions). The change in allele frequency over the mountain pass is more of a sharp ‘cliff’ (pun intended), rather than a sigmoid like cline expected due to balance between dispersal and selection.
- We think some of what the reviewer is seeking ‘distinction between clinal and non-clinal and how steepness is defined relative to each other’ is contained in Figure 3 which shows the relative steepness of thousands of clinal loci. Here we can see the distribution of cline widths with FastClines (curves in the plot margins) and with MLE (for a select set of loci linked to colour genes available). Although markers not showing strong allele frequency differences between outer parental locales were excluded in this (i.e. deltaP <0.9 in Fig3), there are still thousands of ‘clinal’ loci with width estimates, some of which tightly linked to colour genes and other which are not. This provides a distribution of cline widths as seen in the curves on side of Figure 3a, which range from steep to shallow simply based on ranked order. Many of the colour linked loci are situated towards the steep end of the distribution of cline widths, whereas those unlinked to colour genes tend to be shallower. We believe this is showing the information regarding how steep clines are relative to each other.
- As we mention above, we cannot fit clines with FastClines for all polymorphic loci, and for KASP loci with MLE fits, we only designed primers for loci linked to colour and a few others identified from FastClines as amongst the steepest.

Finally on the topic of clines "wide" and "shallow" are uses interchangeably, and this might be fine if there were no cline fits with {p0,p1} free to vary. BUT there are such fits, and a fit with p0=0.49, p1=0.51 could reasonably be called shallow irrespective of its width estimate. I suggest use only wide.

RESPONSE:

- This is a good point. We reword to only use the term ‘wide’ rather than shallow throughout manuscript.

These classical issues on how to describe concepts in this field naturally permeate the whole manuscript, leading to a very very long line-by-line breakdown below. The length of this list, in itself, should not be disheartening. In almost all cases I have made simple constructive suggestions that could be easily implemented.

I am left with the opinion however, that not all the authors read all of the sections of this R1 version of the manuscript, perhaps preferring to leave a level of proofing to the review process. This is not the role of reviewers, and is also a risky strategy. Not all reviewers can see through the typos , misued and simply missing words to the brilliant science underneath.

Finally finally, I mentioned one aspect of the new approach which would benefit from clearer explanation: The bias of the FastClines width estimator when the centre estimate approaches the limits of the sampling localities. The estimators are so clearly laid out and explained in figure 2, and in the methods, it is really surprising their properties cannot be described analytically. Certainly the current description of 'centre and width becoming confounded' (L555-561) can be massively improved.

RESPONSE:

- This is a good point. We do explain how the method was derived and its original form in some level of detail from Polechova & Barton 2011, but believe further analytical origins of this method can be read in that paper.
- Regarding the confounding nature of cline and width towards the edge, we have improved the explanation of this phenomena on L652-656, also see this again in response to similar point below
- “This is likely explained by the coarse spatial sampling (i.e. six pools), where width and centre can be confounded, resulting in narrowing of clines due to step changes in frequency between the outer two sampling locations. In other words, heterozygosity is accumulated in a small geographic region towards the sampling edge, generating what resembles narrow clines.

Line-by-line typos comments and suggestions
[Comments are in square brackets]
{Comments after completing the overview are in curly brackets}

L7 Double comma between authors.

- Corrected

L41 "on magenta side" -> "on the magenta side"

- Corrected

L71 "steep geographic clines" [The reader asks: steep compared to what? One answer is Sigma, but Sigma is not introduced until line 76. Drop the word steep or end line 71 with a colon to let the reader know an explanation of 'steep' is on the way - steep compared to clines for neutral loci (L79). See also comment for L167.

- Corrected, removed “steep”

[Is a 1km wide cline steep… for a landsnail? For an albatross? Sigma changes the answer]

L188 "the interaction of a few, large effect loci," ->
"the interaction of a few large effect loci,"

- Corrected

L130-131 "steep clines […], is consistent" -> "steep clines in colour phenotype and allele frequencies at ROS [23], EL [24] and SULF [25], are consistent"

- Corrected

L135-136 "How clines are distributed [...], would be useful" ->
"Knowing how clines are distributed and their properties in relation to these features, would be useful"

- Corrected

L167 "the steep transition" Again a declaration that a transition is steep, without any comparator. What might make sense here is "the steeper part of the transition" ie steeper than the tails. See comment for L71.

- Modified to “…to capture the allele frequency change either side of the transition”

L180-182 "the median of 79.7% of sites within the 10,000 bp sliding windows was
covered with 46,100 windows (93.7%) remaining with the minimum of 10% window
coverage"

[Clarify. Slow down. How this is done affects inference.

(1) A decision to use sliding windows, rather than a tiling, was taken and justified?
(2) A decision about the appropriate window scale was taken and justified?
(3) "the median of 79.7% of sites within [decided] windows was
covered with 46,100 windows remaining"…remains very difficult to parse… e.g. how many windows were there in the first place?
]

- For clarity we have modified on L188 to “…to capture the allele frequency change either side of the

“A sliding window approach (10kbp windows) provided a suitable span to identifying differences in diversity and divergence (see Tavares et al., 2018). After filtering (low sequencing depth, singletons and non-biallelic SNPs), a median of 79.7% sites remained within the 10,000 bp sliding windows. This produced 46,100 windows (93.7% of genome), with a minimum of 10% window coverage”

L185 [The paragraph starting] "Allele frequencies were generally similar between the two variants of A. m. majus" . For the new methods described in the manuscript to be generally applicable across hybrid systems, the authors must discuss how the steps in this paragraph should be replicated for systems without a binary phenotypic flag allowing researchers to divide their own data into appropriate categories]. {This is indeed discussed}.

- As pointed out by reviewer, this is covered elsewhere in manuscript

L187-188 "2,319 sites (0.011% of variant sites) exhibiting fixed differences (∆ = 1)" [What is the probability P of the data falling by chance into the two categories described above, as a fixed difference, or does the pooled nature of the data completely prevent this otherwise simple calculation? Of course the probability P will be low, given the quality of data for this study, but the reader would benefit with some idea how low. If, for example, the two fixed differences on chr2 (L191) are going to be ignored from this point onwards, if P is low the reader might question that decision ]

- The probability of site being found as fixed when not, and vice versa would be difficult to calculate from the poolSeq data. We did perform Fischer exact tests, to test significance based on read counts for small numbers. This provides a p-value of the allele frequency difference, but considering we already filtered low depth sites and wanted to retain as many of the sites we decided to go with these numbers. We took the philosophy of lowering false negatives and accepting some false positives, on the premise that its clusters of loci showing differentiation and clinal loci that are most important.

L201 Fig. 2: the Figure is of such low resolution that the sub-indices in the formulae are impossible to read, even zooming in.

- Not sure what has happened here as our version looks very high resolution. We will rebuild the pdf in new submission and double check this.

L201-205 "We use FastClines at 3,847 highly divergent loci (∆ ≥ 0.90 between most distant pools YP4 and MP11) and use cline width to estimate the steepness of the transition and centre to estimate cline position along the transect."

[Rephrase. Cline width has been defined as the inverse of the steepness of the transition for decades. Cline centre has been how a cline position has been measured for decades. Variation in position indicates asymmetric geneflow across the hybrid zone, not along it. The sentence should be something like this "We use FastClines at 3,847 highly divergent loci (∆ ≥ 0.90 between most distant pools YP4 and MP11) to estimate cline widths and centre positions across the transect."

- We have modified as suggested by reviewer

L205-207 "This narrowing of cline widths towards the edges is consistent with simulations of clines with six demes and reflects a limitation of small numbers of demes (S1 Fig)."

[Clarify and rework.

A real hybrid zone exists. This has been sampled in 6 localities. This does not mean the real hybrid zone consists of six demes.

{See first, overview point (1)}: The authors need to explain the relevance to the real hybrid zone, of a simulation of 6 demes. The authors hint at relevance when they state L213 "power increasing with the number of [simulated] demes" this suggests to the reader that the real hybrid zone is best approximated by a chain of many demes.

Then, the appropriate simulation is of a chain of many demes, sampled at 6 places along the chain. It is possible this is what the authors are doing, but it is not what they describe. Lines 206-216 need rewritten, and potentially the underlying simulation reworked.
]

- As we have pointed out above in the earlier response to point 1, this last point is what the simulations are doing. It’s a chain of many demes, sub sampled to ‘locales’ of different numbers to examine the effects of sampling density on cline estimations.
- We have modified as suggested by reviewer on L223: “This narrowing of cline widths towards the edges is consistent with simulations of sub-sampling along a cline (S1 Fig). More specifically, sub-sampling of six locales along a chain of demes produces this effect which diminishes with 12 or 24 locales sampled (see S1 Fig).)”

L213-216 "Cline properties can be distinguished as loci become less differentiated on either side of the cline although decreasing sequencing depth, variance in allele frequencies makes these estimates coarser (S2 Fig)."

[Clarify. The factors which make estimates coarser include "loci [becoming] less differentiated on either side of the cline". Yet this factor is contrasted with the others by the word "although"]

- We clarified this in an updated section here, dropping the ‘although’:
- Despite this artefact, comparisons of simulations of steeper and shallower clines showed quite distinct distributions of width estimates, even with sparse local sub sampling of six or eight locales (S1 Fig). Cline estimates become more difficult to distinguish as the underlying cline properties become more similar, loci become less differentiated on either side of the cline (S1 Fig and S2 Fig), whereas decreasing sequencing depth and variance in allele frequencies makes these estimates coarser.

L230 "loci linked (<100kb)" -> "loci closely linked (<100kb)"
[linked means on the same chromosome, YES/NO. It does not mean statistically associated. See L232 "unlinked" == NO]

- Thanks for noticing this omission. We modified as suggested by reviewer to call this “loci closely linked” or “tightly linked to..” and “not tightly linked to..” throughout manuscript

L229 "traditional Maximum Likelihood Estimates (MLE)" ['Traditional" is doing a lot of heavy lifting here. Is it traditional to assume hybrid zones have symmetric change? Also, only very few analyses allow for the effects of Fst]

- Thanks for noticing this overuse of the ‘traditional’ term. We reduce this down to one usage early on the manuscript. Our fit of a polymorphic sigmoid cline is “traditional” in the sense that it is one class of cline model typically tested in geographic cline studies. We realise that cline models with asymmetrical tails should also be tested. However, we chose to limit this to symmetrical models, because (1) this is more closely aligned with FastClines assumptions, (2) more detailed “traditional” fitting to several alternative cline models (symmetric, asymmetric) is a major undertaking and dealt with in another manuscript currently on BioArxv (Surendranadh et al in prep) and mentioned in this manuscript, where we also discuss the limitation of FastClines (see next point)
- “Spatially dense sampling and testing of alternative cline models indicate that the clines at these colour loci are mostly coincident yet display stepped clines with different asymmetrical tails (Surendranadh et al in prep). Assuming a symmetric sigmoid cline model when asymmetries either side of clines exist, are known to incorrectly push cline centre estimates apart [32]. This is one limitation of the *FastClines* method as it assumes a polymorphic yet symmetric sigmoid cline, suggesting some of the spread of cline centres observed may be driven by these asymmetries.”

L248-253 "The steepest clines overall were observed for loci tightly linked to ROS and EL, the two major effect loci important for regulating magenta pigmentation (Fig 3; Fig 4). […] the steepest clines were for loci tightly linked to ROS and EL, the two major effect loci important for regulating magenta pigmentation (Fig 3b)."

[Repetition. ?Hangover from a previous revision?]

- Thanks for noticing this, we removed offending repetitive sentence.

L254 "Of the clines around ROS and EL, clines were steepest within coding sequences for the MYB genes with mean cline width of 2.2km (±1.2km SD) at ROS1, 1.3km (±0.3km 255 SD) and 3.5km (±2.3km SD) at EL (Fig 4A)"

[Clarify.
The only mention of MYB up to this point is L120-122 "The magenta colour is
primarily controlled by two tightly linked MYB-like transcription factors on chromosome
6, ROSEA (ROS) [22,23] and ELUTA (EL) [24]" … making "MYB genes" both redundant and slightly inaccurate at L254. Further, some readers will wonder how one gene can have a mean width. I think you want to say something like:
"for the variant sites around and within ROS and EL, clines were steepest within coding sequences for the genes, with mean cline width of 2.2km (±1.2km SD) within ROS1, 1.3km (±0.3km 255 SD) and 3.5km (±2.3km SD) within EL (Fig 4A)"

I am aware that MYB is also mentioned in the caption of Fig 4… but the same issue arises there, if you wish to count that as the first mention after L120-122.

RE: "around and within ROS and EL" see also overview point (2)
]

- We have modified the text to follow the reviewers suggestion “For the variant sites around and within *ROS* and *EL*, clines were steepest within coding sequences for the genes, with mean cline width of 2.2km (±1.2km SD) within ROS1, 1.3km (±0.3km 255 SD) and 3.5km (±2.3km SD) within EL (Fig 4A)”

L255-256 "In this region, clinal loci tended to become wider as loci were located further away from the coding sequences of these two genes. " ->
"In this region, variant site clines tended to become wider the further they were from the coding sequences of these two genes. "
[Avoiding confusion arising from overloading of meaning onto the word "as" ->{while,because}]

- Followed the reviewer suggestion to tighten up the wording here “In this region, variant site clines tended to become wider the further they were from the coding sequences of these two genes”

L270-271 "The ability of steep clines using FastClines to detect other major colour loci depended on where the cline centre was located."
[Wow. I think you want something like: "The utility of FastClines width estimates to detect other major colour loci depended on where the cline centre was located."]

- Thanks for catching this one. We modified that offending sentence to “The utility of FastClines width estimates to detect other major colour loci depended on where the cline centre was located”

L270-273 "Cline centre displayed a range of positions either side of the main phenotypic transition. A large number of loci (n = 1843) displayed a cline centre to the east of the phenotype centre (magenta side)."
[Loci do not display things to us. I think wording would be easier if it drew a distinction between estimates and reality. For example, here: "Cline centre estimates were spread on either side of the main phenotypic transition, with a bias toward the eastern (Magenta) side (n=1834 vs n=XXXX)."]

- Thanks for comments, we’ve improved clarity along the lines of the suggested wording here “Cline centre estimates were spread on either side of the main phenotypic transition, with a bias toward the eastern (Magenta) side (n=1834 vs n=391 near phenotypic centre)”

L275 "(95%) with cline centre >14 km,"
[14 km is not the measure of interest. The reader wants to know how many kms east of the phenotypic transition (actually, they want to know how many Sigma, but kms will do).]

- We now clarify the distance from the phenotype centre with “…..(95%) with cline centre between 14.5 to 15.km (~2 to 3 km East of phenotypic centre at 12.5km)”

L277 " a change in clines centre < 13 km"
[See above… if @L275 >14 means as extreme as, or more extreme, and @L277 <13 means as extreme as, or more extreme, then we can work out the authors expect 13>centres<14. But it is not for the reader to work this stuff out. Just state the centering being used, or even better, give the readers the distances with respect to the centering used, but not mentioned, in this paragraph.

- As in above point, we clarify more clearly how far in km the cline centre is shifted relative to phenotype centre “In contrast, clinal loci within the coding sequence of *FLA* and upstream show clines centres < 13 km (~1.5 km West of phenotype centre at 12.5km; see *FLA2* for upstream sites on Fig3A; see S5 Fig).”

L289-290 "Next we focus on loci with clines positioned closer to the phenotype transition between 8-16km."
[This directly follows a paragraph entirely focussed on 13 and 14 kms, both of which lie within the range 8-16kms. Is it possible 8-11kms is intended here, as found later on L299?]

- The 8-16km range is correct, we are just excluding the tails (<8km centres and >16km centres) due to this artefact of narrow clines as centres approach the boundaries. The reference to 8-11km was intended to focus on yellow centred clines.

L305 "substantially narrower than the other clines in this region 10.24km (±0.91km SD)" -> "substantially narrower than the average of other clines in this region at 10.24km (±0.91km SD)"

- Corrected to “substantially narrower than the average of other clines in this region at 10.24km (±0.91km SD)"

L306-307 "The remaining known loci, AUN and SULF showed no clines with the FastClines estimates. For the latter, the SULF colour locus was not well covered by poolSeq data…"
[The impression given is that FastClines has failed, but this is not the case for SULF; coverage was the issue. Also: explain the issue for AUN.]

- We also have no clines for AUN overall. Non detected, no significant sequence divergence either. It may be that it’s a smaller effect variant without steep clines. This is now explained in the text “The AUN genomic region doesnt exhibit any steep clines or strongly differentiated loci.”

L317 "linked to colour genes ROS, EL, SULF, recently identified genes FLA, CRE, and Chr5CC." -> "linked to colour genes ROS, EL, SULF, recently identified genes FLA and CRE, and the Chr5CC identified in this study."
[At minimum an "and' is missing from the original sentence. But there is more going on. The impression given is that Chr5CC (a term coined 16 lines previously) is being classified as recently identified, alongside two recently published genes. I am aware this impression is unlikely to be intended, but not all readers are aware of the significance of the Oxford comma].

- Correct to include that missing ‘and’ - “were compared at loci linked to colour genes *ROS*, *EL*, *SULF*, and recently identified genes *FLA*, *CRE*, and Chr5CC”

L320-322 "The exception was the SULF locus which was considerably shallower for the locus with MLE estimates compared to the majority of clines from poolSeq with FastClines."
[Clarify. Loci vs variant sites]{See overview comment (2)}

- We appreciate this comment and follow the reveiwer guide for clearing up the descriptors of genomic regions/loci. We followed their suggestion on pg xx with some further modifications:
  - "The exception was the SULF gene (interval/region) which was considerably wider for the KASP site (with MLE width estimates) compared to the majority of poolSeq sites (with FastClines width estimates)."

L324-326 "In comparison to cline widths, the range of cline centres from FastClines were overestimated compared with MLE cline fits. The latter method of cline fitting showing a much tighter grouping of centres much closer towards the phenotypic centre (Fig 3)."
[This is a key statement, which should tie in to the discussion. {It does}. It also has some minor grammar stuff going on.
"the range" is singular, so it "was overestimated", not "were overestimated"]

- We appreciate this comment and follow the reviewer guide and improved clarity to “In comparison to cline widths, the range of cline centres from *FastClines* **was** overestimated compared with MLE cline fits”

L332-334 "Divergent loci (∆ ≥ 0.9) with steep clines were found on all chromosomes (Fig 5). Cline width ranged from steep to shallow across all chromosomes (Fig 5A), whereas cline centre tended to display different values across chromosomes (Fig 5B)."
These statements contain no information other than a definition of divergent, and that estimates vary. See previous comments on 'steep' being comparative, and cline centering being relative.

- We appreciate the thought that not much information is contained in these two sentences. However, it still needs to be stated somewhere that steep clines are found on all chromosomes (rather that some missing clines).

L339-340 "we divided each chromosome into 100kb and 10 kb blocks and counted the number of loci with allele frequency differences"
[Here a tiling, not a sliding window, is the decision. Why? See also L180-182]

- We have improved clarity and now mention why tiling was used. Its because we later use this for permutations and cant use overlapping sliding windows: “…100kb and 10 kb blocks (non-overlapping for permutations, see below)”

L363-365 "Clinal loci were significantly more clustered in the genome together compared to expectations based on random positions across the genome on chromosomes 2 and 6, but not significantly clustered on the remaining chromosomes"
[Where is the distinction between clinal and non-clinal defined? I guess this is a threshold on the estimated width? Also: "clustered in the genome together" -> "clustered together in the genome"]

- Defined more clearly on L443“Divergent loci exhibiting clinal properties (centres 8 - 16km, excluding step changes over mountain pass) were significantly more clustered”
- Also corrected “….more clustered together in the genome…” on L444

L365-367 "This suggests that some chromosomes exhibit tight concentrations of clines in localized areas of the genome, whereas others exhibit scattered clines along the entire chromosome"

[L363-365 does more than suggest this pattern, it describes this pattern. This is also the place to tell the reader the relationship between the sites with fixed differences between phenotype categories and these regions where sharp clines cluster]

- Modified wording to on L447 to: “This **indicates** that some chromosomes exhibit….”

371-373 "Dense clusters of clines in small genomic regions on each chromosome tagged ROS, 372 EL, and SULF, and recently discovered CREMOSA (CRE) [27], FLA [26] and a gene located at Chr5CC (see below)."

[If "Dense clusters of clines in small genomic regions on each chromosome" are the subjects of this sentence, what is the verb? Is it true that dense clusters are found on each chromosome?... it is implied. Perhaps the intended meaning is this:
"Dense clusters of clines are found in small genomic regions tagged by genes ROS, 372 EL, and SULF, and recently discovered CREMOSA (CRE) [27], FLA [26] and a gene located at Chr5CC (see below)."]

- Corrected the sentence on L452 to: “Dense clusters of clines are found in small genomic regions tagged *ROS*, *EL*, and *SULF*, and recently discovered *CREMOSA (CRE)* [27], *FLA* [26] and…..”

L423-424 "This region, was the top ranked cluster of clines on chromosome 5, with"
[Here and throughout: subdivision of sentences with commas differs between grammars, but in English it can make sentences very dis-jointed. The first comma here is a good example]

- Corrected and removed the superfluous comma

L541-542 "using simulated annealing procedure [e.g. 20]." -> "using a simulated annealing procedure [e.g. 20]." OR "using simulated annealing procedures [e.g. 20]."

- Corrected and inserted the ‘a’ to: “…using a simulated annealing…

L543-544 "estimates of cline properties with as few as six demes along a transect through a hybrid zone."
[Deme is not the correct word here. The zone being studied is not demic in the sense of being best approximated by a series of distinct demes with migration vectors between them. It is certainly not best approximated by six demes. If that were the case a model of discrete steps between six demes would be best, not models of continuous change. What there are six of is: sampling localities].

- As mentioned above to main point #1, we have modified the manuscript throughout to replace the term ‘deme’ with ‘sampling localities’

L545 "several caveats can be distinguished." Whether they can be distinguished is not the issue. -> "several caveats should be noted."

- Good spotting, we have switched this to : “…several caveats should be noted.”

L555-561 "For the Antirrhinum hybrid zone, our comparison of cline fitting methods indicated that FastClines produced a narrowing of clines widths when cline position (centre) moves towards the edges of the transect. This is likely explained by the coarse spatial sampling (i.e. six pools), where width and centre can be confounded, resulting in narrowing of clines due to step changes in frequency between the outer two demes. Simulations of FastClines estimates generated this same artefact, but only with small numbers of pools (i.e. ≤8 pools)."
[Why precisely are cline width estimates reduced when cline centre estimates approach edge samples?
Stating this is _likely_ due to something (anything) implies the precise reason has not been identified, yet the nature of the c and w estimates is clear and straightforward.
Stating width and centre can be confounded is highly non-intuitive and so does not help the reader understand the source of this bias in the method.]

- We specify in more detail the reasoning behind cline width being confounded with centre towards the edges on L652 “This is likely explained by the coarse spatial sampling (i.e. six pools), where width and centre can be confounded, resulting in narrowing of clines due to step changes in frequency between the outer two sampling locations. In other words, when heterozygosity is accumulated in a small geographic region towards the sampling edge, this generates narrower clines.

L564-566 "For Antirrhinum, inspection of individual loci frequencies also show many of these loci exhibit sharp allele frequency step changes between pool YP4 and YP2 rather than a sigmoid-like change over geographic space."
[Loci vs variant sites issue]

- Individual “loci frequencies” corrected to “allele frequencies”

L569 "outer populations on yellow side" -> "outer populations on the yellow side"

- Corrected

L583-587 "The pattern at many background loci (unlinked to colour loci) exhibiting a sharp allele frequency step over the mountain pass (chromosomes 3, 7, 8), may be a signal of an introgressive sweep of magenta alleles into yellow flanking populations that has been blocked at the mountain pass."
[Another key passage, but difficult to relate to the results section, which focuses on distances eg 8-11kms, whereas the mountain pass is never explicitly connected a distance or range].

- We now explicitly mention in several sections in the manuscript text early on L185 and L 350:” Thus, a break of ~4 kilometres in the distribution of *Antirrhinum* plants coincides with a mountain pass (~3 to 7km along transect)” and remind in the results and figure caption 1

615-617 "will require careful consideration initial divergence ( ∆ ) local recombination landscape and the genetic architecture of barriers." -> "… consideration of initial divergence …"

- Modified as suggested

624-625 "the range of cline centres were likely overestimated"
"the range" is singular, so it "was likely overestimated", not "were likely overestimated".
See also L324-326]

- Modified as suggested

L629-630 "Assuming a symmetric sigmoid cline model when asymmetries either side of clines exist, are known to incorrectly push cline centre estimates apart [32]." -> "Assuming…is known"

- Modified as suggested, swapped “are” with “is” in : “Assuming a symmetric sigmoid cline model when asymmetries either side of clines exist, **is** known to incorrectly push cline centre estimates apart”

L644-646 "This could be due to the RUB allele from A. m. m var. pseudomajus having limited phenotypic effect on predominantly A. m. m var. striatum (yellow) parental genotypes and thus is unseen by selection" -> "This could be due to the RUB allele from A. m. m var. pseudomajus having limited phenotypic effect on predominantly A. m. m var. striatum (yellow) parental genotypes and thus being unseen by selection"

- Shortened as suggested to “This could be due to the *RUB* allele from *A. m. m var. pseudomajus* having limited phenotypic effect on predominantly *A. m. m var. striatum* (yellow) parental genotypes and thus being unseen by selection”

LL690-693 "more clines may be expected in areas with low diversity and lower rates of recombination and these features need to be considered when interpreting the density of clines along chromosomes."
[High divergence should be added to this list]

- Added divergence “Regardless of the evolutionary forces responsible for the signal, more clines may be expected in areas with high sequence divergence, low diversity and lower rates of recombination and these features need to be considered when interpreting the density of clines along chromosomes”

L701-703 [Not demes, sampling localities - or just locales]

- Updated as mentioned above, to sampling locations or localities

L715-716 "Each of the six demes included a random sub-set of n = 50 individuals within a 50 metre radius from a larger sample."
[Clarify. Perhaps the meaning is this: "Within the bounding polygon of the georeferences of all available tissues, six points were chosen with appropriate spacing, and such that 50 individuals could be subsampled within 50 meters of each point.]

- We appreciate the wording provided here and follow the suggestion with “Within the bounding polygon of the georeferences of all available tissues, six points were chosen with appropriate spacing, and such that 50 individuals could be subsampled within 50 meters of each point”

Materials and Methods
L731 "and to fit descriptive clines" -> "and to fit maximum likelihood clines" [See also comment for L800-802]

- We have modified this to MLE or maximum likelihood clines. Removed the term descriptive throughout manuscript

L731 "and to verify FastClines estimates from poolSeq" [Is there an issue for users of FastClines who do not have such a verifitcation database?]

- No issue, like with any method looking for a signal, its nice to verify with another approach

L740 "the most outer populations" -> "the outermost populations"

- Modified as suggested

L744 "They showed that the width of a cline can" -> "They showed that the width of a single locus gaussian cline can"
[Though the Barton and Polechová results are very general, they are not general over all dispersal kernels (nor, I believe, over multilocus clines where D distorts the shape). This appears confirmed at L778-783]

- Modified as suggested, to more clearly described the previous theory

L800-802 "Descriptive cline fitting" Make the name chosen for this cline fitting approach consistent between the methods and the main text. "Descriptive" is perhaps not the best choice, as both types of cline fitting, MLE and FastClines are descriptive. They are just different approximations to a {c,w} description.

- Agreed, throughout manuscript we have removed “descriptive” and gone with MLE to describe the previous method

L821-823 "For the parameter, we fitted a beta-binomial error term to account for the variance in allele frequencies across demes and to controlfor population structure along the cline."
[What is missing here is how this estimate affects the fit of the model]

- We believe this is already explained in that sentence. This accounts for variation in allele frequencies (Fst) and thus control for pop structure. For better clarity we modify the sentence to mention it provides an improved model fit “For the $F_{ST}$ parameter, we fitted a beta-binomial error term to account for the variance in allele frequencies across sampling locations and to control for population structure along the cline and provide an improved model fit”

L823-825 "Prior to commencing cline fitting algorithm, the larger data set was randomly thinned within each deme to reduce the computational time, resulting in 10,000 individual genotypes across ~120 demes."
[Clarify. Is the larger dataset the same lrager dataset mentioned aealier in the methods - ie all samples available. It seems not: thinning is within locales… but how this leads to 120 locales is unclear.]

- Clarified the details further: “Prior to commencing cline fitting algorithm, the larger data set was randomly thinned within each locality to reduce the computational time, and filtered to remove localities with less than 10 individuals, resulting in 10,000 individual genotypes across ~120 localities.”

L871 "sampling from field sites as is each deme were" -> "sampling from field sites as if each locale were"

- Changed deme to “locality”

L891 "test how cline width is improved with density of geographic sampling" -> "test how cline width estimation is improved with density of geographic sampling"

- Changed deme to include ”estimation” in "test how cline width estimation is improved with density of geographic sampling"

L959 "Lastly, to test whether RUBIA on yellow backgrounds," [Words missing]

- Included missing words: “whether *RUBIA* **effects magenta intensity**”

L959-961 "we genotyped and isolated parental genotypes of A. m. m. striatum (rosst

Elst/rosst Elst) and segregating for RUB alleles" [Words missing]

- Fixed sentence to: “….on yellow backgrounds, we genotyped and isolated parental genotypes of *A. m. m. striatum* (*ros^st^ El^st^/ros^st^ El^st^*) that were segregating for *RUB* alleles (n = 105 individuals) and similarly ranked flowers for magenta intensity within each multi-locus genotype class.”

L967 "clinal loci" see comments on how clinal loci are defined.

- Corrected as mentioned previously

Reviewer #4:

This is an extremely well-written paper that introduces a cline-fitting method for genome-scale data. This method is desperately needed for the advancement of hybrid zone research. I predict that this new method and program will be widely adopted and that this paper will be widely cited. PLoS Biology makes a lot of sense for this manuscript, because the journal is read by evolutionary biologists working across biological diversity.

- We appreciate the positive reviewer comments

I really do not have any comments. I found one typo on line 813, but that's it.

- Corrected typo “Meters”

Nice job.

Robb Brumfield
