# Supplementary material for: Genome-wide cline analysis identifies new locus contributing to a barrier to gene flow across an Antirrhinum hybrid zone
Source: PLoS Genet. 2026 Jul 13;22(7):e1012173. doi: 10.1371/journal.pgen.1012173 (PMC13387609; doi:10.1371/journal.pgen.1012173)
Supplement: Peer review history

# Author comment: Author response to Decision Letter 0

**DOI:** 10.1371/journal.pgen.1012173.r001

**Transfer Alert**

This paper was transferred from another journal. As a result, its full editorial history (including decision letters, peer reviews and author responses) may not be present.

22 Oct 2025

Attachment Submitted filename: Prior Peer Review and Response letter FieldEtal 21_10_2025.docx

# Peer review: Decision Letter 0

**DOI:** 10.1371/journal.pgen.1012173.r002

**Authors:** Justin Fay, Nicolas Bierne

23 Dec 2025

PGENETICS-D-25-00935

Genome-wide cline analysis identifies new locus contributing to a barrier to gene flow across an Antirrhinum hybrid zone

PLOS Genetics

Dear Dr. Field,

Thank you for submitting your manuscript to PLOS Genetics. After careful consideration, we feel that it has merit but does not fully meet PLOS Genetics's publication criteria as it currently stands. Therefore, we invite you to submit a revised version of the manuscript that addresses the points raised during the review process.

Please submit your revised manuscript within by Jan 22 2026 11:59PM. If you will need more time than this to complete your revisions, please reply to this message or contact the journal office at plosgenetics@plos.org. When you're ready to submit your revision, log on to https://www.editorialmanager.com/pgenetics/ and select the 'Submissions Needing Revision' folder to locate your manuscript file.

Please include the following items when submitting your revised manuscript:

* A letter that responds to each point raised by the editor and reviewer(s). You should upload this letter as a separate file labeled 'Response to Reviewers'. This file does not need to include responses to formatting updates and technical items listed in the 'Journal Requirements' section below.

* A marked-up copy of your manuscript that highlights changes made to the original version. You should upload this as a separate file labeled 'Revised Manuscript with Track Changes'.

* An unmarked version of your revised paper without tracked changes. You should upload this as a separate file labeled 'Manuscript'.

If you would like to make changes to your financial disclosure, competing interests statement, or data availability statement, please make these updates within the submission form at the time of resubmission. Guidelines for resubmitting your figure files are available below the reviewer comments at the end of this letter.

We look forward to receiving your revised manuscript.

Kind regards,

Nicolas Bierne

Academic Editor

PLOS Genetics

Justin Fay

Section Editor

PLOS Genetics

Aimée Dudley

Editor-in-Chief

PLOS Genetics

Anne Goriely

Editor-in-Chief

PLOS Genetics

**Additional Editor Comments:**

Dear Dr Field,

I am pleased to inform you that I have obtained three reviews of your article, 'Genome-wide cline analysis identifies new locus contributing to a barrier to gene flow across an Antirrhinum hybrid zone', which has been transferred from PloS Biology to PloS Genetics. Two of the reviewers are the same as in the previous review round and one is new. Despite the delay in obtaining all the reports, you will see that only a short list of minor issues needs to be addressed to ensure this manuscript is accepted. I am delighted that this study on the genomic analysis of geographic clines is to be published in PLOS Genetics. I agree with reviewers 1 and 2 that you should discuss the differences between YP4 and YP2 in more detail, as well as the effect of a barrier to dispersal at the mountain, and you should also engage in a more thorough cross-discussion with Pal et al.'s article in Molecular Ecology. It seems to me that a multilocus barrier could indeed be trapped at the barrier to dispersal at the mountain, with colour loci clines having escaped this barrier and positioned themselves between YP1 and MP2. However, having read the two articles, I get the impression that you favour the hypothesis of local introgression (genome swamping) in YP1 and YP2. Consequently, the differences between YP2 and YP4 would be solely due to geographical isolation rather than genetic barriers. All of this needs to be clarified and better discussed. This would also shift the focus of the discussion away from the method and the detection of colour loci (which have been detected regardless of the approach used) towards discussing the potential of genome-wide cline analysis to interpret genetic barriers (I mean that if a multilocus genetic barrier is caught on a mountain, we can't expect to fit clines too well, and that the approach works for rather soft barriers and in areas without too many obstacles to dispersal).

Thank you for submitting your very interesting study to PloS Genet. I look forward to reading the revised version for upcoming publication.

Best regards,

Nicolas Bierne

**Journal Requirements:**

1) Please provide an Author Summary. This should appear in your manuscript between the Abstract (if applicable) and the Introduction, and should be 150-200 words long. The aim should be to make your findings accessible to a wide audience that includes both scientists and non-scientists. Sample summaries can be found on our website under Submission Guidelines:

https://journals.plos.org/plosgenetics/s/submission-guidelines#loc-parts-of-a-submission

2) Some material included in your submission may be copyrighted. According to PLOSu2019s copyright policy, authors who use figures or other material (e.g., graphics, clipart, maps) from another author or copyright holder must demonstrate or obtain permission to publish this material under the Creative Commons Attribution 4.0 International (CC BY 4.0) License used by PLOS journals. Please closely review the details of PLOSu2019s copyright requirements here: PLOS Licenses and Copyright. If you need to request permissions from a copyright holder, you may use PLOS's Copyright Content Permission form.

Please respond directly to this email and provide any known details concerning your material's license terms and permissions required for reuse, even if you have not yet obtained copyright permissions or are unsure of your material's copyright compatibility. Once you have responded and addressed all other outstanding technical requirements, you may resubmit your manuscript within Editorial Manager.

Potential Copyright Issues:

- Please confirm (a) that you are the photographer of Figures 7, 8, and Supporting Information file., or (b) provide written permission from the photographer to publish the photo(s) under our CC BY 4.0 license.

3) Please amend your detailed Financial Disclosure statement. This is published with the article. It must therefore be completed in full sentences and contain the exact wording you wish to be published.

- State the initials, alongside each funding source, of each author to receive each grant. For example: "This work was supported by the National Institutes of Health (####### to AM; ###### to CJ) and the National Science Foundation (###### to AM)."

- State what role the funders took in the study. If the funders had no role in your study, please state: "The funders had no role in study design, data collection and analysis, decision to publish, or preparation of the manuscript.".

If you did not receive any funding for this study, please simply state: u201cThe authors received no specific funding for this work.u201d

**Reviewers' comments:**

Reviewer's Responses to Questions

**Comments to the Authors:**

**Please note here if the review is uploaded as an attachment.**

Reviewer #1: Overview

I stand by my original review of this work for PLoS Biology:

“This is an excellent study applying new methodology to, and reporting new biology of, the Antirrhinum system. The planning, layout and argumentation are lucid. The FastClines approach elegantly plugs an important computational hole in genomic studies of hybridisation. The follow-up experiments are excellent. The new results regarding a magenta enhancer gene, and a set of displaced clines, are convincing. I have no hesitation in recommending this for publication.

That being said...some classical issues on how to describe concepts in this field are not dealt with well. Further, there is one aspect of the new approach which would benefit from clearer explanation...”

I find the author’s responses to my review and three others calm and well justified. I am shocked that the PLoS Biology decision claimed an equal split in the reviewer’s opinions, given that Reviewer1, point2 (scholarship!) demonstrated Reviewer1’s opinion on this topic is of little or no relevance due to their lack of grasp of what a genome-scale cline fitting study is. As an aside, I also (independently of the authors) checked Reviewer1’s point2 ‘supporting’ references and found them entirely inappropriate, and possibly hallucinatory.

There IS a computational problem with maximum likelihood cline fitting. Software that fits only two cline parameters (eg genomic clines) is already error prone because it is under- parameterized (Baird and Daley). Increasing the number of parameters to 4 to reduce such errors will make such software un-usable at genome scale, because it is already almost unusable with 2 parameters, and convergence times explode with further parameters. In contrast, the authors’ work allows for 4 parameters without an optimization step. That resolves a huge computational issue by a qualitative step forward. Reviewer 4 and I agree on these facts. It is no coincidence that we both have hands-on experience with cline fitting.

The authors responded very positively to my suggestions for further clarification of the manuscript. This involved tiny changes at many places – fiddly work mostly well implemented. There remains work to be done, however, as the agreed changes have not been implemented in a quite large section of the manuscript (roughly L225-435).

Typos/suggestions

Online Abstract:

“Clines at RUBIA approached towards fixation on magenta side of the hybrid zone, whilst remaining polymorphic on the yellow side” -> “Clines at RUBIA approached towards fixation on THE magenta side of the hybrid zone, whilst remaining polymorphic on the yellow side”

[The manuscript contains a second copy of the abstract which has this correction]

Manuscript:

L211 “…to estimates and centre positions across the transect.” -> L211 “to estimate widths and centre positions across the transect.”

L211-213 “Cline widths ranged from ~12.5km to 0.8km, narrowing as the cline centres moved either side of the hybrid zone (< 8km and >15km) (Fig 3).” ->

“Cline width estimates ranged from ~12.5km to 0.8km, narrowing as the cline centres moved either side of the hybrid zone (< 8km and >15km) (Fig 3).”

[The author’s state these changes are artifactual, ie it is not the width per se which is suggested to change, but rather the FastCline estimate of the width]

L221-225 “Cline estimates become more difficult to distinguish as the underlying cline properties become more similar, loci become less differentiated on either side of the cline (S1 Fig and S2 Fig), whereas decreasing sequencing depth and variance in allele frequencies makes these estimates coarser.”

A contrast ‘whereas’ is drawn between aspects of similar effect. First, to say that more similar clines are more difficult to distinguish is a tautology. Second, less differentiation and decreased sequencing depth both make cline more difficult to distinguish. Finally: local variance in allele frequencies increases with cline width, and so is not independent of the other factors being discussed.

L243-245 “FLA1 refers to the cluster of loci on one side of the gene situated > 13km centre positions, and FLA2 refers to the cluster of loci on the other side of the gene with < 13km centre positions.”

->

“FLA1 refers to the cluster of loci on one side of the gene having centre positions > 13km, and FLA2 refers to the cluster of loci on the other side of the gene with centre positions < 13km.”

L258-259 “For 391 clinal loci centred closer to the core region near the phenotypic centre”

[core geographic region, as previously discussed]

L263-264 “In this region”

[genome region, as previously discussed]

L278-280 “The ability of steep clines using FastClines to detect other major colour loci depended on where the cline centre was located. Cline centre displayed a range of positions either side of the main phenotypic transition. Cline centre estimates were spread on either side of the main phenotypic transition, with a bias toward the eastern (Magenta) side (n=1834 vs n=391 near phenotypic centre).”

[This section is a hangover from the previous round of review. The first sentence does not parse. The remainder is repetitive. I think the authors want to say something like:

“The ability of FastClines to detect other major colour loci depended on their cline centre estimates, which showed a range of positions either side of the main phenotypic transition, with a bias toward the eastern (Magenta) side (n=1834 vs n=391 near phenotypic centre).”]

L300 “Given the tendency for widths to narrow”

->

“Given the tendency for width estimates to narrow” [See passim]

L314 “Similarly, in the core region” -> “Similarly, in the core geographic region”

[core geographic region, as previously discussed]

L308-316 “For example, filtering only for loci with centres located in the yellow flanks of the hybrid zone between 8-11km, the steepest clines in this region were all clustered around a narrow genomic region on chromosome 5, which we term Chr5CC (Chromosome 5 Cline Cluster). Clines within this region displayed an average width of 7.52km (±1.04km SD), substantially narrower than the average of other clines in this region at 10.24km (±0.91km SD)". Similarly, in the core region, the CRE locus involved in yellow pigmentation displayed an average cline width of 7.52km (±1.04km SD), substantially narrower than the other clines in this region 10.24km (±0.91km SD) (Fig 4B).”

The confusion over “regions” highlighted in the previous round of review has not at all been addressed here despite author response “We follow this advice throughout and use the terminology ‘geographic regions’ and ‘genomic regions’ to clarify the differences throughout the new version of the manuscript.”

Above, the first sentence describes a focus on those clines with centre estimates falling in a geographic region (8-11km). The first use of “region” refers in fact to this focal set of clines. The next two “regions” refer to genomic region Chr5CC. One sentence later we have “Similarly, in the core region…” but this refers to a geographic region, the “core geographic region” defined (L258-269) as 11-14kms. In between we have this sentence

“Clines within this region displayed an average width of [X], substantially narrower than the average of other clines in this region [Y]”

What is the distinction between the two sets of clines ‘within this region’ which makes their difference in width interesting to the reader?

The confusion is heightened because clines are referred to as “in” the core (geographic) region. They are not. Their centre estimates fall in the core geographic region. A cline can be described as “in” a genomic region, if that is where the SNP showing the cline is located. A cline cannot be described as “in” a geographic region because by definition clines span all the geography. I think the authors want to say something like:

“For example, considering only loci with centre estimates located in the yellow flanks of the hybrid zone between 8-11km, the steepest clines were all clustered within a narrow genomic region on chromosome 5, which we term Chr5CC (Chromosome 5 Cline Cluster).

Clines within this genomic region displayed an average width of 7.52km (±1.04km SD), substantially narrower than 10.24km (±0.91km SD), the average width of other clines with 8-11km centre estimates.

Similarly, in the core geographic region (11-14kms), the CRE locus involved in yellow pigmentation displayed an average cline width of 7.52km (±1.04km SD), substantially narrower than 10.24km (±0.91km SD), the average width of other clines centered in this geographic region (Fig 4B).”

However, once the results are clearly labelled (like this, or any other clarification), we see a further problem: The average cline widths are copy-pastes of each other! ie 7.52km vs 10.24km for two different genome regions (Chr5CC vs CRE) and two different cline subsets: those with centres in 8-11km vs centred in 11-14km.

This is a key passage of the results. Ignoring requests for clarification here is pure self-harm.

L317 “doesnt” -> “doesn’t”

L332-335 “The exception was the SULF gene interval (on Chr 4) which contained loci considerably steeper for the KASP site (with MLE width estimates) compared to the majority of poolSeq sites (with FastClines width estimates).”

Loci are not steep. The gene interval contains KASP SNPs with steeper clines.

L336 “from SULF region” -> “from the SULF region”

L336-341 “In comparison to cline widths, the range of cline centres from FastClines was overestimated compared with MLE cline fits. The latter method of cline fitting showing a much tighter grouping of centres much closer towards the phenotypic centre (Fig 3). However, the step change in cline centre positions either side of the FLA gene were consistent between both cline fitting approaches (FLA1 locus and FLA2 locus on Fig 3).”

The first sentence is not relative to cline widths, it is a comparison of centre estimate ranges. I think the authors want to say something like:

“FastClines centre estimates had a wider range than those from MLE cline fitting. MLE cline fitting showed a much tighter grouping of cline centre estimates around the phenotypic centre (Fig 3). However, the step change in cline centre positions either side of the FLA gene were consistent between both cline fitting approaches (FLA1 locus and FLA2 locus on Fig 3).”

Again this is a crucial result for those interested in sources of error when estimating cline properties. Clarity is important.

L345 “Divergent loci (∆ ≥ 0.9)”

[Does the ‘divergence’ of a SNP depend on its allele frequency? I think not. I think what is being referred to is lineage sorting and/or diagnosticity]

L345-346 “Cline width ranged from steep to shallow”

[This despite author response “We reword to only use the term ‘wide’ rather than shallow throughout manuscript.”]

L345-347 “Cline width ranged from steep to [wide] across all chromosomes (Fig 5A), whereas cline centre tended to display different values across chromosomes (Fig 5B).”

Again ‘whereas’ is being used to highlight a non-existent contrast: The text states ‘widths were variable whereas centres were variable’ (across all chromosomes).

L358 “clines are often localized” -> “clines are often clustered”

[They are always localized – their positions are known]

L376-377 “Divergent loci exhibiting clinal properties (centres 8 - 16km, excluding step changes over mountain pass)”

[Explain to the reader why clines centered on the mountain pass were excluded, considering we expect HZs to be centered exactly on such barriers/density troughs… without explanation this decision just looks very weird]

L382-385 “Geographic clines, irrespective of properties, were largely clustered in close proximity to the six known genes involved in regulating flower colour. The majority, n = 2918 (76.2%) of divergent loci with clines were located within 300kb of known colour genes (Fig 5).”

[Again an issue from previous review unresolved. The text makes a distinction between ‘divergent loci’ with versus without clines, but the criteria for this distinction are nowhere described. A flat line is simply a cline of great width. Unless the criterion is clarified, the following text is wierded, for example (L383) “Dense clusters of clines are found in small genomic regions” should be ‘dense clusters of CRITERION=STEEP clines…”].

L394 “To understand how pair-wise relative differentiation (FST) associates with colour loci…”

[Tell the reader what the pairs are. In this study the pairs are tables of gene frequency estimates taken from the sets of arbitrary ‘locales’. The reader should be made aware that different (arbitrary) choices of locale boundaries will give different Fst estimates, as this informs us of sources of uncertainty within the Fst comparisons made.

This is one of the key advantages of FastClines over Fst scans: To calculate Fst as described here one has to make a decision about what ‘populations’ to compare… especially in a hybrid zone, those decisions are both arbitrary and consequential].

L398 “Comparing all nine pair-wise populations of yellow and magenta pools…”

NO the pools ARE the ‘populations’ analysed with Fst.

-> “Considering all nine pair-wise Fst comparisons of yellow and magenta pools…”

L398-400 “…yielded 481 to 694, 10kb windows with excess FST, of which zero to three windows overlapped with colour genes.”

-> “…yielded 481 to 694, 10kb windows with excess FST.”

[(1) an overlap of zero to three is meaningless without context. The text goes on to describe the actual pattern.

(2) remind the reader how ‘excess FST’ is defined]

L405,418 “Some windows of excess FST were identified even between comparisons of two pools from the same variety”

NO. Fst is not measure between comparisons….it IS the comparison.

-> “Some windows of excess FST were identified even when comparing two pools from the same variety”

L419-421 “Comparing with more relaxed outlier cut-off for filtering 10kb windows with excess FST with 95th% quantile, improved the number of outlier windows in each colour gene region (S14 Fig)”

(1) The reader has not been reminded what the other cutoff was, so reporting this one is of low information. (2) The number of outlier windows is increased, not improved. (3) There will be a trade-off with relaxation of the (arbitrary) choice of outlier threshold – this appears unreported, but is why ‘improvement’ is not the appropriate word – the trade-off will not be an improvement.

L424 “many FST outlier windows contain no clines.” A flat line is a cline of large width. As above, such statements make no sense unless the criterion for a pattern of variation to be described as ‘is/is not a cline’ is made explicit.

L434-435 “We interrogated a set of clines from Chr5CC showing the steepest widths in the geographic region left of the core around 9-10km centre position with FastClines (Fig 3)”

Widths are not steep, they are narrow. Clines can be steep. I think the authors want to say something like:

“Using FastClines we interrogated a subset of the steepest clines from Chr5CC centered around 9-10km ie left of the core geographic region (Fig 3)”

Then, again, clarity highlights a further issue: The geographic region is a new one: 9-10km versus 8-11km mentioned earlier.

L473-474 “As in b, the flowers were ranked” -> “As in (B), the flowers were ranked”

L489 “predicts that RUB genotype” -> “predicts that the RUB genotype”

L494 “over the pass” [Remind the reader of the geographic interval of the pass]

L574-575 “heterozygosity is accumulated in a small geographic region towards the sampling edge, generating what resembles narrow clines.”

[While it is nice to see some explanation of this effect, this explanation likely leaves the reader more, rather than less, confused. What biological phenomenon would leave heterozygosity accumulated at the edges?]

L580-582 “inspection of individual allele frequencies also show many of these loci exhibit sharp allele frequency step changes between pool YP4 and YP2 rather than a sigmoid-like change over geographic space.”

[A step between two discrete localities is consistent with an (intermediates unobserved) steep sigmoid change. As before, theory would predict a tension zone would become trapped at such a density trough/barrier to gene flow – why no Discussion of this?].

L593 “(i.e. >100kb away from known genes)” -> “(i.e. >100kb away from known colour genes)”

L607-608 “Considering the age of the hybrid zone is at least 100 generations old [27], even the most weakly selected colour loci should have reached equilibrium.”

Not local linkage equilibrium. That has half life around 10,000 generations at 10kb distance. See eg the authors’ own text at LL621-622.

L622-623 “For a barrier locus, lower recombination rates increase the extent of linked selection on neutral loci,”

As one of the current authors previously made very clear (Stankowski, Chase et al. 2019), calling indirect selection ‘linked selection’ is unnecessarily confusing, especially in a hybrid zone where admixture LD facilitating indirect selection is generated genome wide… and so has nothing to do with loci being linked. See also (Baird & Daley).

L626-629 “Theory predicts the effects of linkage around a single site to be more localised than for a region that contains two or more tightly linked barrier loci, as multiple loci generate a stronger barrier [27] and hence influence the number of clinal loci surrounding the causal loci.”

NO. Linkage is not what generates multilocus barriers. In the absence of LD, linkage has no effect whatsoever.

Theory predicts the effects of indirect selection around a single site to be more localised than for a region that contains two or more tightly linked barrier loci, because recombination will decay admixture LD less effectively in the multilocus barrier region. Then, indirect selection will lead to a stronger barrier in the multilocus case, affecting more of the genome.

L646-647 “This is one limitation of the FastClines method as it assumes a polymorphic yet symmetric sigmoid cline,”

Different levels of fixation (ILS) are allowed in the tails…this is cline asymmetry.

L749 “descriptive clines” this despite authors’ response ‘Removed the term descriptive throughout manuscript’

L763 “gaussian cline” [Check this?… I think the result is for a logistic cline. Yes, its definitely logistic, see LL800-801]

L859 “Prior to commencing cline fitting algorithm” -> “Prior to commencing the cline fitting algorithm”

L865-866 “We begin the algorithm with a random set of parameters which are changed randomly…” [repetition]

L960-961 “The location of all known genes known to influence flower colour” [repetition]

References

Baird, S. J. E. and N. Daley "The Shapes of Clines and Wavefronts." Molecular Ecology n/a(n/a): e70109.

Stankowski, S., M. A. Chase, A. M. Fuiten, M. F. Rodrigues, P. L. Ralph and M. A. Streisfeld (2019). "Widespread selection and gene flow shape the genomic landscape during a radiation of monkeyflowers." PLoS biology 17(7): e3000391.

Reviewer #2: I reviewed the manuscript by Field et al. exploring the indirect signature of reproductive isolation on genetic diversity using an exhaustive genome scan of allele frequency clines in the classical snapdragon color-morph hybrid zone. The study demonstrates that using a relatively small number of pool-seq samples and a novel procedure for allele frequency cline fitting based on expected total heterozygosity efficiently captures the main reproductive isolation (RI) loci in the species. The authors also describe a new color locus affecting the intensity of magenta pigmentation. The robustness of the clines fitting and the new RI locus detected was further validated by looking at some KAPS sequencing over a large number of samples.

First, I must apologize for the delay. When I first downloaded the manuscript, I apparently received a previous version (e.g., some characters were overlapping in Figure 2, and I had no access to the previous reviews from the PLoS Biology submission). Consequently, I commented on issues that I later noticed had already been addressed in the current submission (such as the benchmarking of the method). Overall, I find the manuscript clear and the method sound, and I have only very limited comments, which I list below. I believe this manuscript will make a valuable contribution to PLoS Genetics.

Minor comments:

L86: "most still use specific loci of interest or sparse reduced representation genomic data" This sentence is somewhat misleading because the present manuscript also applies a stringent threshold (Dfreq >0.9) prior to performing cline fitting, which similarly reduces the genomic representation being analyzed.

Fig3: When examining the frequency variation of Chr5CC shown in Fig. 3B, the most differentiated loci appear to be very close to the Dfreq threshold of 0.9, yet this region shows extreme outlier behavior in terms of cline width.

I wonder how much the arbitrary threshold of 0.9 affects the power to detect regions under selection? In addition, I would like the authors to justify a bit more on the choice of the DFREQ of 0.9, which I find quite stringent. Others sometime use Dfreq > 0.3.

Specific comment/

L115: I would recommend providing some information here about what this new method entails and how it differs from existing approaches.

I suggest providing a brief explanation of total heterozygosity and how it relates to the 4pq formula.

L209: Same line: maybe the bracket could mention the benchmark, and it could be interesting to mention how much faster does the cline fit procedure is at this stage.

L392-426: I am not fully convinced of the added value of comparing window-based FST with SNP-based cline analyses. Moreover, I think a comparison between the ARG-based inferences/blocks inferred from the ARG (recently published in Molecular Ecology by the same authors) and the cline results would have been more interesting.

Fig 5: It is not immediately clear to me why the x-axis labels in the first two boxplots differ from those in the second panel, nor why flanking regions are not shown for all genes. Does this relate to the deltaP > 0.9 threshold, where sometimes no clinal markers were found to the right or left of the selected gene? Ultimately, the figure is somewhat difficult to read, primarily because some categories are missing and the reason for their absence is not obvious. A brief explanation in the figure legend clarifying which categories are excluded and why would improve readability.

Fig. 5: Why is the YP1-MP2 comparison not shown?

L501: Should this be "RUBPS" like in L511 rather than "RUBP"?

L511 : This sentence seems oddly placed given that the two alleles have already been presented above. Should it appear earlier in the text?

Reviewer #3: The authors have addressed the major comments from the previous submission, and this has shaped up to be a solid contribution.

As a reviewer, not a co-author, I cannot force the authors to write a different paper. But I still believe that t the biological advance here is modest, and that a stronger contribution would have been a more weighty methodological contribution. it seems like the authors decided against that. Because this paper is so polished, and because this is the paper the authors are going to write, it is hard to know what to do with this, but I am find myself suggesting acceptance at PLoS Genetics. However, I would understand if other reviewers or the AE suggest PLoS one (although this is way more substantive than a typical PLoS One paper) or G3 (which seems like a more natural home).

**********

**Have all data underlying the figures and results presented in the manuscript been provided?**

Large-scale datasets should be made available via a public repository as described in the *PLOS Genetics*  data availability policy, and numerical data that underlies graphs or summary statistics should be provided in spreadsheet form as supporting information.

Reviewer #1: Yes

Reviewer #2: Yes

Reviewer #3: Yes

**********

PLOS authors have the option to publish the peer review history of their article (what does this mean?). If published, this will include your full peer review and any attached files.

If you choose “no”, your identity will remain anonymous but your review may still be made public.

**Do you want your identity to be public for this peer review?**  For information about this choice, including consent withdrawal, please see our Privacy Policy.

Reviewer #1: **Yes:** Stuart J.E. Baird

Reviewer #2: No

Reviewer #3: No

[NOTE: If reviewer comments were submitted as an attachment file, they will be attached to this email and accessible via the submission site. Please log into your account, locate the manuscript record, and check for the action link "View Attachments". If this link does not appear, there are no attachment files.]

**Figure resubmission:**

While revising your submission, we strongly recommend that you use PLOS’s NAAS tool (https://ngplosjournals.pagemajik.ai/artanalysis) to test your figure files. NAAS can convert your figure files to the TIFF file type and meet basic requirements (such as print size, resolution), or provide you with a report on issues that do not meet our requirements and that NAAS cannot fix.

-->

After uploading your figures to PLOS’s NAAS tool - https://ngplosjournals.pagemajik.ai/artanalysis, NAAS will process the files provided and display the results in the "Uploaded Files" section of the page as the processing is complete. If the uploaded figures meet our requirements (or NAAS is able to fix the files to meet our requirements), the figure will be marked as "fixed" above. If NAAS is unable to fix the files, a red "failed" label will appear above. When NAAS has confirmed that the figure files meet our requirements, please download the file via the download option, and include these NAAS processed figure files when submitting your revised manuscript.-->

**Reproducibility:**

To enhance the reproducibility of your results, we recommend that authors deposit laboratory protocols in protocols.io, where a protocol can be assigned its own identifier (DOI) such that it can be cited independently in the future. Additionally, PLOS ONE offers an option to publish peer-reviewed clinical study protocols. Read more information on sharing protocols at https://plos.org/protocols?utm_medium=editorial-email&utm_source=authorletters&utm_campaign=protocols

# Author comment: Author response to Decision Letter 1

**DOI:** 10.1371/journal.pgen.1012173.r003

6 May 2026

Attachment Submitted filename: Field_WGS_PloSGen_Response_to_reviewers.docx

# Editor report: Decision Letter 1

**DOI:** 10.1371/journal.pgen.1012173.r004

**Authors:** Justin Fay, Nicolas Bierne

21 May 2026

Dear Dr Field,

We are pleased to inform you that your manuscript entitled "Genome-wide cline analysis identifies new locus contributing to a barrier to gene flow across an Antirrhinum hybrid zone" has been editorially accepted for publication in PLOS Genetics. Congratulations!

Before your submission can be formally accepted and sent to production you will need to complete our formatting changes, which you will receive in a follow up email. Please be aware that it may take several days for you to receive this email; during this time no action is required by you. Please note: the accept date on your published article will reflect the date of this provisional acceptance, but your manuscript will not be scheduled for publication until the required changes have been made.

Once your paper is formally accepted, an uncorrected proof of your manuscript will be published online ahead of the final version, unless you’ve already opted out via the online submission form. If, for any reason, you do not want an earlier version of your manuscript published online or are unsure if you have already indicated as such, please let the journal staff know immediately at plosgenetics@plos.org.

In the meantime, please log into Editorial Manager at https://www.editorialmanager.com/pgenetics/, click the "Update My Information" link at the top of the page, and update your user information to ensure an efficient production and billing process. Note that PLOS requires an ORCID iD for all corresponding authors. Therefore, please ensure that you have an ORCID iD and that it is validated in Editorial Manager. To do this, go to ‘Update my Information’ (in the upper left-hand corner of the main menu), and click on the Fetch/Validate link next to the ORCID field.  This will take you to the ORCID site and allow you to create a new iD or authenticate a pre-existing iD in Editorial Manager.

If you have a press-related query, or would like to know about making your underlying data available (as you will be aware, this is required for publication), please see the end of this email. If your institution or institutions have a press office, please notify them about your upcoming article at this point, to enable them to help maximise its impact. Inform journal staff as soon as possible if you are preparing a press release for your article and need a publication date.

Thank you again for supporting open-access publishing; we are looking forward to publishing your work in PLOS Genetics!

Yours sincerely,

Nicolas Bierne

Academic Editor

PLOS Genetics

Justin Fay

Section Editor

PLOS Genetics

Aimée Dudley

Editor-in-Chief

PLOS Genetics

Anne Goriely

Editor-in-Chief

PLOS Genetics

www.plosgenetics.org

BlueSky: @plos.bsky.social

----------------------------------------------------

Comments from the reviewers (if applicable):

Dear Dr Field,

Please accept my apologies for the delay in responding. I quickly decided that this revised manuscript did not need to be sent back for further review, but I needed time to proofread it myself. After some hesitation over minor points, I concluded that this new version was ready for acceptance. This work will mark a turning point in the study of hybrid zones by opening (finally, I’d like to say) the way for the genomic study of clines.

Best regards,

Nicolas Bierne

----------------------------------------------------

**Data Deposition**

If you have submitted a Research Article or Front Matter that has associated data that are not suitable for deposition in a subject-specific public repository (such as GenBank or ArrayExpress), one way to make that data available is to deposit it in the Dryad Digital Repository. As you may recall, we ask all authors to agree to make data available; this is one way to achieve that. A full list of recommended repositories can be found on our website.

The following link will take you to the Dryad record for your article, so you won't have to re‐enter its bibliographic information, and can upload your files directly:

http://datadryad.org/submit?journalID=pgenetics&manu=PGENETICS-D-25-00935R1

More information about depositing data in Dryad is available at http://www.datadryad.org/depositing. If you experience any difficulties in submitting your data, please contact help@datadryad.org for support.

Additionally, please be aware that our data availability policy requires that all numerical data underlying display items are included with the submission, and you will need to provide this before we can formally accept your manuscript, if not already present.

----------------------------------------------------

**Press Queries**

If you or your institution will be preparing press materials for this manuscript, or if you need to know your paper's publication date for media purposes, please inform the journal staff as soon as possible so that your submission can be scheduled accordingly. Your manuscript will remain under a strict press embargo until the publication date and time. This means an early version of your manuscript will not be published ahead of your final version. PLOS Genetics may also choose to issue a press release for your article. If there's anything the journal should know or you'd like more information, please get in touch via plosgenetics@plos.org.

# Editor report: Acceptance letter

**DOI:** 10.1371/journal.pgen.1012173.r005

**Authors:** Justin Fay, Nicolas Bierne

PGENETICS-D-25-00935R1

Genome-wide cline analysis identifies new locus contributing to a barrier to gene flow across an Antirrhinum hybrid zone

Dear Dr Field,

We are pleased to inform you that your manuscript entitled "

Genome-wide cline analysis identifies new locus contributing to a barrier to gene flow across an Antirrhinum hybrid zone" has been formally accepted for publication in PLOS Genetics! Your manuscript is now with our production department and you will be notified of the publication date in due course.

The corresponding author will soon be receiving a typeset proof for review, to ensure errors have not been introduced during production. Please review the PDF proof of your manuscript carefully, as this is the last chance to correct any errors. Please note that major changes, or those which affect the scientific understanding of the work, will likely cause delays to the publication date of your manuscript.

Soon after your final files are uploaded, unless you have opted out or your manuscript is a front-matter piece, the early version of your manuscript will be published online. The date of the early version will be your article's publication date. The final article will be published to the same URL, and all versions of the paper will be accessible to readers.

For Research Articles, you will receive an invoice from PLOS for your publication fee after your manuscript has reached the completed accept phase. If you receive an email requesting payment before acceptance or for any other service, this may be a phishing scheme. Learn how to identify phishing emails and protect your accounts at https://explore.plos.org/phishing.

Thank you again for supporting PLOS Genetics and open-access publishing. We are looking forward to publishing your work!

With kind regards,

Sharmila Kamatchi

PLOS Genetics

On behalf of:

The PLOS Genetics Team

Carlyle House, Carlyle Road, Cambridge CB4 3DN | United Kingdom

plosgenetics@plos.org | +44 (0) 1223-442823

plosgenetics.org | Twitter: @PLOSGenetics
